# Effect of a photoswitchable rotaxane on membrane permeabilization across lipid compositions

**DOI:** 10.3762/bjoc.21.192

**Published:** 2025-11-11

**Authors:** Udyogi N K Conthagamage, Lilia Lopez, Zuliah A Abdulsalam, Víctor García-López

**Affiliations:** 1 Department of Chemistry, Louisiana State University. Baton Rouge, LA 70803, USA

**Keywords:** lipid membrane, membrane fluidity, membrane permeability, photoswitchable rotaxane

## Abstract

This study investigated a rotaxane featuring azobenzene photoswitches in its macrocycle and its ability to modulate membrane permeability in large unilamellar vesicles (LUVs) of varying lipid compositions. Upon photoisomerization, the rotaxane significantly enhanced the release of the hydrophilic dye sulforhodamine B in vesicles composed of EYPC/Chol 8:2, with release increasing from 29% (non-irradiated) to 59% (irradiated). Moreover, gel-phase DPPC bilayers also exhibited an increase in release from 7% to 14%. On the contrary, highly fluid pure EYPC bilayers showed high baseline release upon rotaxane incorporation (64%), with photoswitching producing only a slight increase (70%), as most dye was released within the first minutes of rotaxane insertion. Likewise, azobenzene photoswitching did not induce permeabilization in the more rigid and thicker EYPC/Chol 6:4 bilayers, which showed minimal release (5%). Furthermore, we discovered that when the unthreaded axle is irradiated with light, an unknown, irreversible photochemical process occurs, which triggers the release from only the vesicles containing EYPC. These findings underscore the significance of both the physical and chemical properties of the bilayer in enabling effective light-triggered cargo release through rotaxane activation.

## Introduction

Lipid membranes play a vital role in biology by acting as protective barriers for cells and organelles while regulating the passage of substances. Additionally, their structure and dynamics influence the activity of membrane proteins that are responsible for essential cellular functions [1]. As a result, the function and properties of lipid membranes are significantly influenced by their lipid composition, which varies across various domains of life, including mammalian, prokaryotic, and plant cells, and even between organelles within the same cell [2].

Therefore, there is a great need to develop molecular tools capable of modulating membrane structure in a controlled manner, either to facilitate cargo transport (such as drug delivery) [3–4], to irreversibly disrupt membranes and induce cell death (e.g., for targeting bacteria or malignant cells like cancer) [5–6], or to influence membrane protein function and thereby control cellular behavior [7–8]. One promising approach is the development of light-activated molecules that can modulate membrane properties upon irradiation, enabling remote activation with high spatiotemporal precision [9].

Rotaxanes have emerged as promising tools for performing various functions in lipid membranes, owing to their multiple sites for derivatization, which allow fine-tuning of their structure and function in membranes. For instance, they have been used to transport ions across lipid bilayers through the shuttling of the macrocycle carrying the ions or through a relay mechanism [10–11]. In one example, the isomerization of an azobenzene photoswitch incorporated into the axle was used to modulate the shuttling rate of the macrocycle and, consequently, the efficiency of potassium ion transport [12]. Our group later demonstrated that non-switchable rotaxanes can transport chloride anions and exhibit bactericidal activity against methicillin-resistant *Staphylococcus aureus* (MRSA) [13]. In a separate study, Smith reported that incorporating non-switchable rotaxanes can induce phospholipid translocation across membranes [14].

Our group recently developed photoswitchable rotaxane **1**, which differs from most reported designs that typically incorporate photoswitchable units into the axle [15]; in our rotaxane **1**, the azobenzene photoswitches are incorporated into the macrocycle instead. We demonstrated that photoisomerization of the azobenzene moiety modulates lipid packing and permeability, leading to reversible changes in membrane structure and vesicle size. However, these initial studies were limited to model lipid bilayers of the same composition.

Given the wide variability in membrane composition and rigidity across different cell types, it is crucial to understand how rotaxane **1** interacts with diverse membrane environments and modulates their properties to guide its future biomedical applications. In particular, we sought to determine whether rotaxane **1** can induce membrane permeabilization in bilayers of varying rigidity and whether membrane phase influences its photoswitching behavior. To address these questions, we investigated the light-induced switching of rotaxane **1** in large unilamellar vesicles (LUVs) composed of lipids representative of distinct membrane phases. Membrane permeabilization was assessed by monitoring the release of encapsulated sulforhodamine B dye, serving as a reporter for membrane perturbation. Specifically, we examined LUVs in the liquid-disordered (L_d_), liquid-ordered (L_o_), and gel (L_β_) phases. As control molecules, we also investigated the membrane perturbation caused by rotaxane **2**, which lacks azobenzene switches, and axle **3**, which lacks the macrocycle. Attempts to study the macrocycle alone without the axle were hindered by its very low solubility in the aqueous buffer.

This study highlights both the potential and the limitations of rotaxane **1** as a membrane-active actuator. It provides key insights for the rational design of next-generation rotaxane-based molecular tools.

## Results and Discussion

### Molecular design

**Figure 1:**
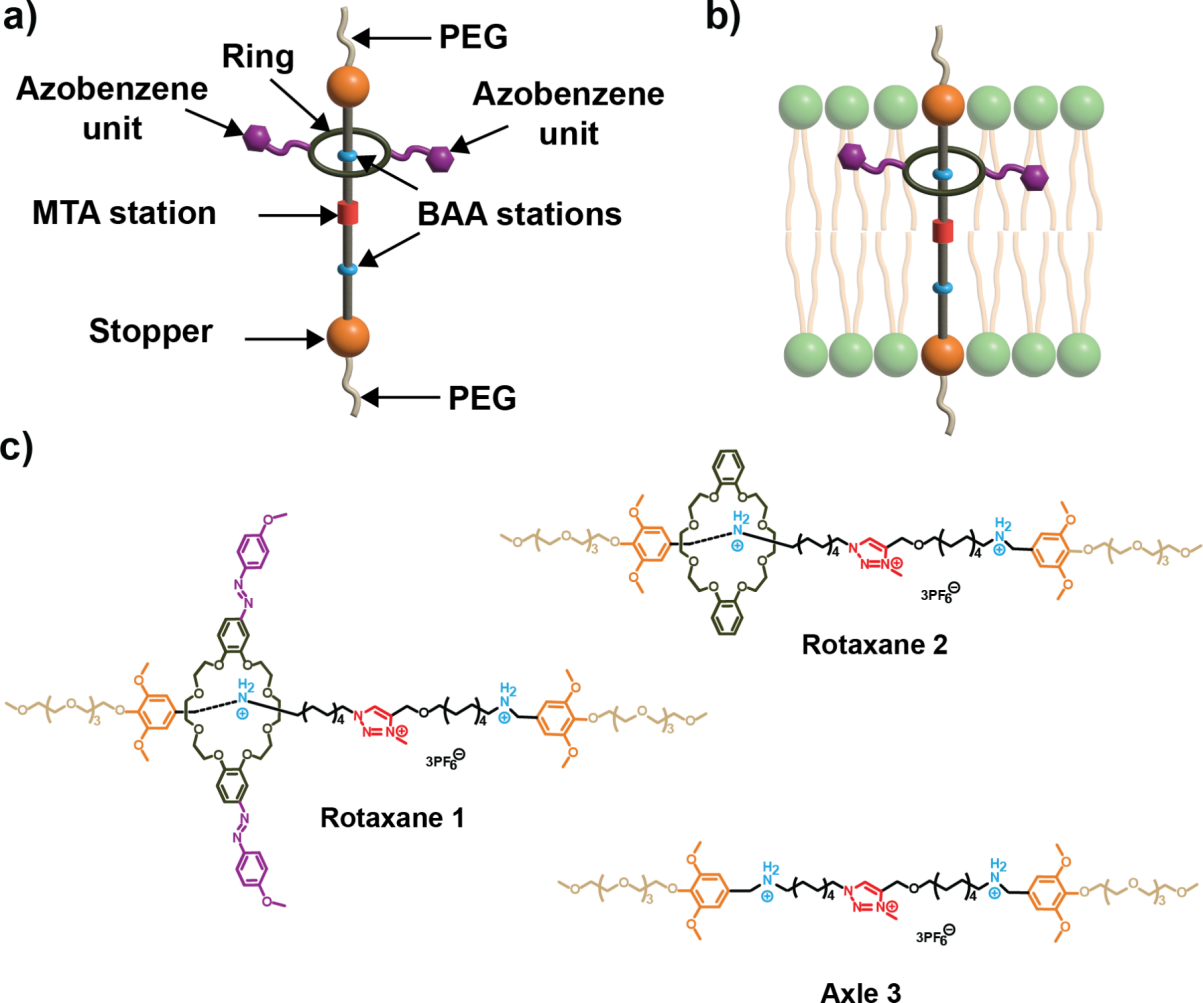
a) Structural components of the rotaxanes (PEG, polyethylene glycol chain; BAA (benzylalkylammonium hexafluorophosphate); MTA (*N*-methyltriazolium hexafluorophosphate). b) Illustration of a rotaxane within a lipid bilayer. c) Chemical structures of rotaxane **1**, rotaxane **2**, and axle **3**.

Rotaxane **1** features a dibenzo 24-crown-8 ring bearing two azobenzene photoswitches, each with methoxy groups in the *para*-position [15]. In contrast, control rotaxane **2** [13] has the same dibenzo 24-crown-8 ring but lacks the azobenzene units. Both rotaxanes use axle **3**, which consists of an amphiphilic chain with three positively charged sites serving as recognition sites for the macrocycle: two benzylalkylammonium groups (BAA) and one *N*-methyltriazolium (MTA) group. The amphiphilic spacer carries an *ortho*-dimethoxybenzene group on each side as stoppers to prevent the macrocycle from slipping off. These stoppers are connected to terminal polyethylene glycol (PEG) chains designed to interact with the membrane–water interface (Figure 1).

### Effect of membrane composition on azobenzene photoswitching

**Figure 2:**
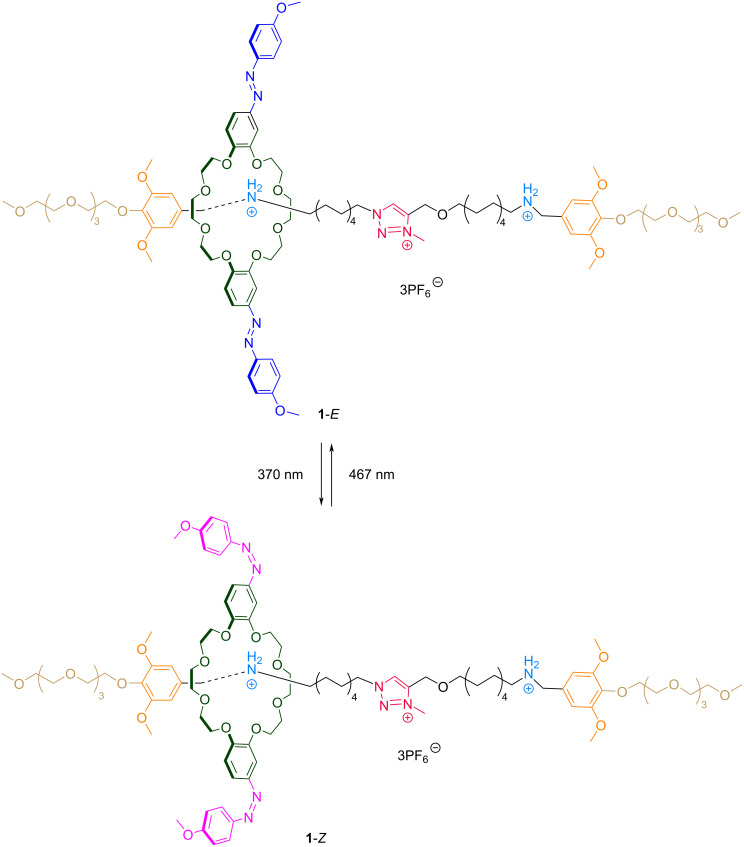
Photoisomerization of rotaxane **1**.

We investigated the photoisomerization behavior of rotaxane **1** (Figure 2) over ten irradiation cycles in large unilamellar vesicles (LUVs) with varying lipid compositions using UV–vis spectroscopy. Specifically, we prepared vesicles composed of pure EYPC (egg yolk phosphatidylcholine), EYPC/cholesterol (Chol) mixtures at 8:2 and 6:4 molar ratios, and pure DPPC (dipalmitoylphosphatidylcholine). The EYPC-only bilayer represents a liquid-disordered (L_d_) phase [16]. Incorporating cholesterol increases membrane rigidity and thickness, promoting the formation of liquid-ordered (L_o_) domains [16]. To further assess the effect of membrane rigidity on rotaxane behavior, we also studied LUVs composed solely of DPPC lipids, which form a highly ordered gel phase (L_β_) at room temperature due to their saturated acyl chains [17].

**Figure 3:**
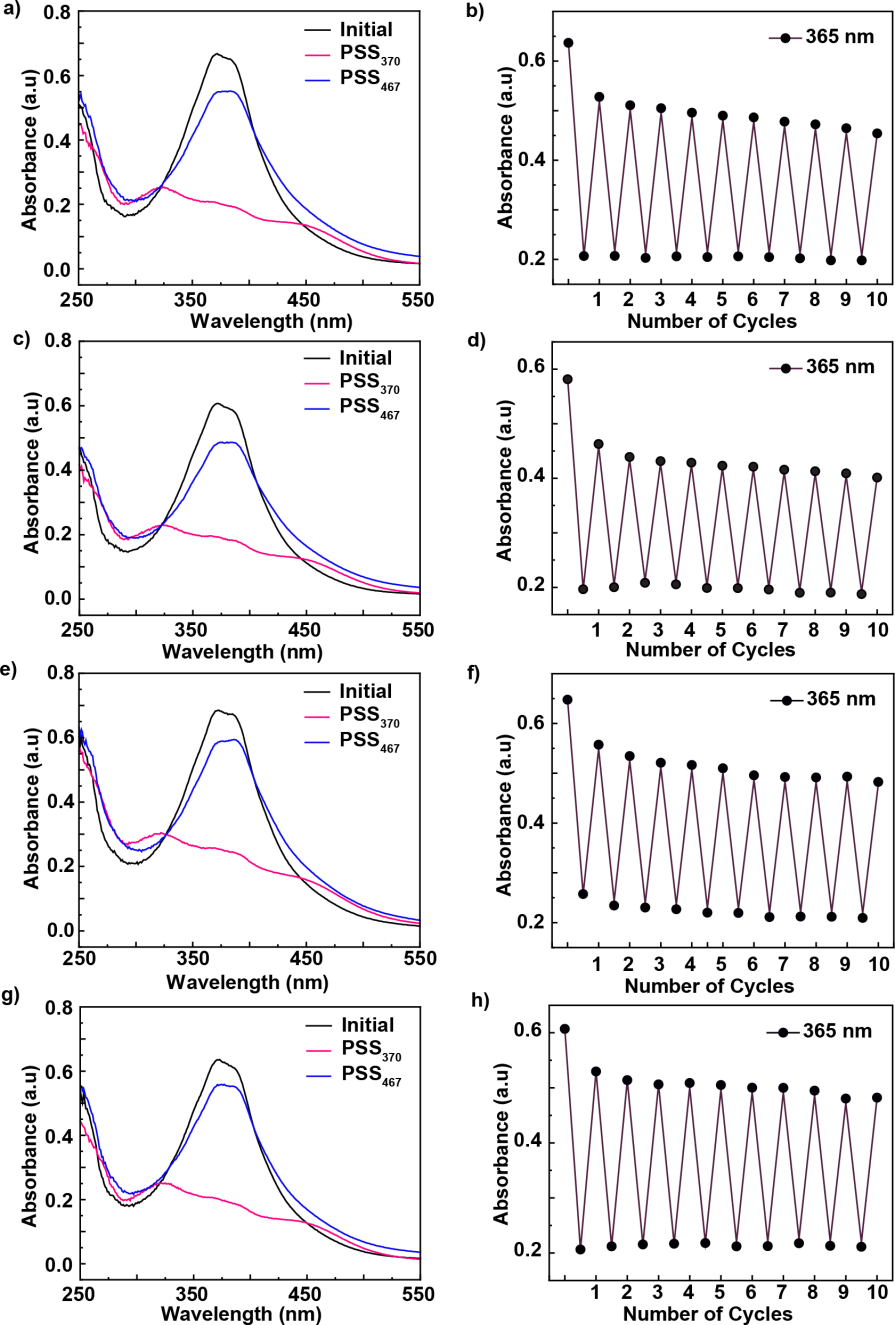
Reversible photoswitching of rotaxane **1** in LUVs with varying lipid compositions. Left column: UV–vis spectral changes of rotaxane **1** after one cycle of photoisomerization – irradiation at 370 nm (PSS_370_) for 1 minute, followed by irradiation at 467 nm (PSS_467_) for 1 minute. Spectra are shown for a) EYPC c) EYPC/Chol 8:2, e) EYPC/Chol 6:4, and g) DPPC. Right column: Absorbance changes at 365 nm over ten consecutive photoisomerization cycles for the same lipid compositions: b) EYPC, d) EYPC/Chol 8:2, f) EYPC/Chol 6:4, and h) DPPC. All experiments were performed at 25 °C using LUVs suspended in HEPES buffer (10 mM, pH 7.2), and the rotaxane (10 mol % with respect to the total lipid concentration) was added dissolved in DMSO.

Rotaxane **1** was incorporated into the LUVs at 10 mol % relative to the total lipid content. UV–vis spectra recorded before irradiation showed an absorption band around 372 nm, corresponding to the π→π* transition of the *E* isomer (Figure 3, left column, black trace). Upon irradiation at 370 nm for 1 minute, two new peaks appeared at approximately 322 nm and 447 nm, attributed to the π→π* and n→π* transitions of the *Z* isomer, respectively (Figure 3, left column, PSS_370_). Subsequent irradiation at 467 nm for 1 minute regenerated most of the *E* isomer, though not to its original quantity (Figure 3, left column, PSS_467_). This incomplete conversion is typical for this type of photoswitches, owing to the spectral overlap between the π→π* and n→π* transitions. Importantly, these spectral signatures were consistent across all lipid compositions, indicating that the different membrane phases did not induce measurable shifts in the absorption of rotaxane **1**.

LUVs containing rotaxane **1** were subjected to ten cycles of alternating irradiation at 370 nm and 467 nm, with UV–vis spectra recorded after each exposure. Figure 3 (right column) shows the changes in absorption at 365 nm, reflecting the photoswitching and photoreversibility of rotaxane **1** across all tested lipid compositions. Overall, rotaxane **1** exhibited efficient and reversible photoswitching over ten cycles with minimal photofatigue, regardless of the membrane environment.

### Sulforhodamine B release from LUVs of varying lipid composition in the absence of light

**Figure 4:**
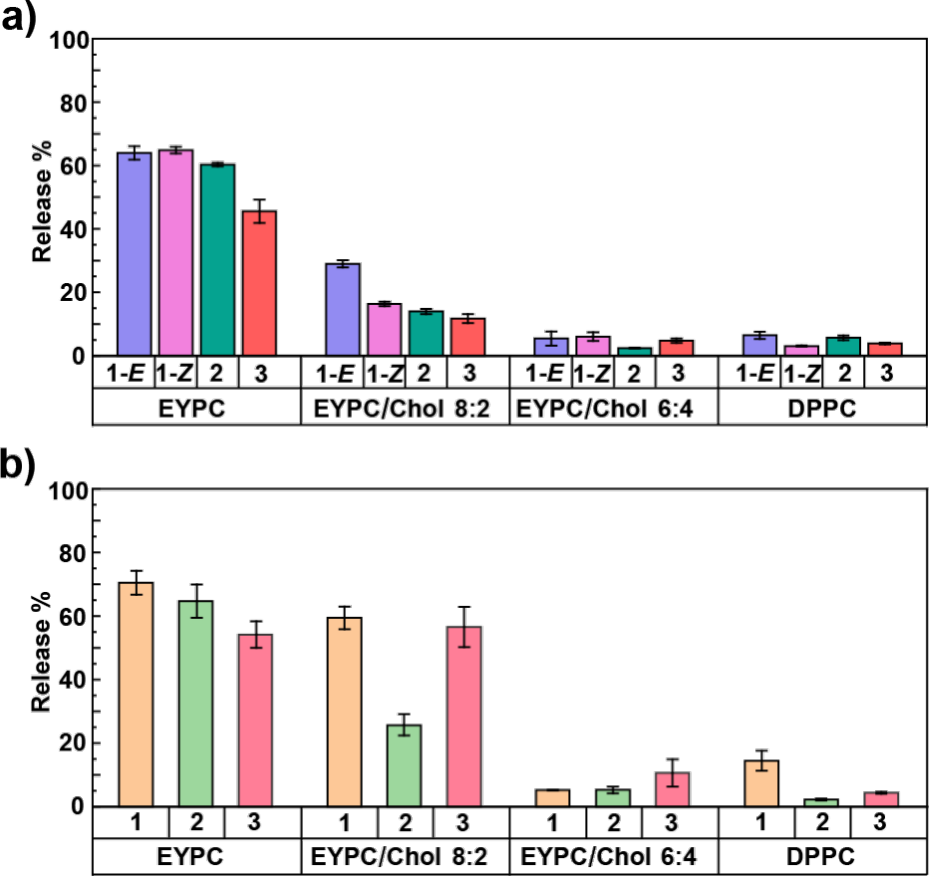
Summary of sulforhodamine B release from LUVs of varying lipid compositions. a) Dye release after 70 minutes following the addition of rotaxanes **1**-*E*, **1**-*Z*, **2**, and axle **3**, in the absence of light irradiation; b) dye release after the addition of rotaxane **1**, **2**, and axle **3** and exposure to five alternating light-irradiation cycles (370 nm and 467 nm).

We assessed the effects of the compounds on membrane permeability over time in the absence of light irradiation, ensuring that no photoswitching occurred. LUVs containing sulforhodamine B were prepared using the lipid compositions described earlier. The release of the dye was monitored over 70 minutes after adding the studied compounds, including rotaxane **1**-*E*, rotaxane **1**-*Z*, rotaxane **2**, and axle **3**. Rotaxane **2** and axle **3** were used as controls to understand the effect of subcomponents of rotaxane **1** on membrane permeabilization and dye release (Table 1, Figure 4a, and Supporting Information File 1, S2.2). In LUVs composed solely of EYPC, both isomers of rotaxane **1** promoted similar levels of dye release (64% for **1**-*E* and 65% for **1**-*Z*) (Figure S1a in Supporting Information File 1). Similarly, control rotaxane **2** caused 60% release, while axle **3** produced a lower, yet still substantial, release of 46% (Table 1, Figure 4a, and Supporting Information File 1, Figure S4). The data also shows that most of the release for rotaxane **1** and **2** occurred immediately upon rotaxane insertion, whereas release caused by axle **3** increased gradually. Bilayers composed solely of EYPC exist in the liquid-disordered (L_d_) phase, characterized by high fluidity and loosely packed lipids, which makes them inherently more permeable [16]. The insertion of rotaxanes or axle further increases this permeability, indicating that all molecules effectively disrupt the bilayer of this lipid composition. However, the rotaxanes with the macrocycle threaded onto the axle caused more membrane perturbation compared to the axle alone, in agreement with our previous molecular dynamics simulations, which highlight the importance of the macrocycle to modulate lipid packing [15].

**Table 1:** Sulforhodamine B release from LUVs of varying lipid composition.^a^

LUVs Composition	Release %						

	no light irradiation (70 minutes)				light irradiation (5 cycles, 68 minutes)		
	**1**-*E*	**1**-*Z*	**2**	**3**	**1** (*E↔ Z*)	**2**	**3**

EYPC	64 (± 2.13)	65 (± 1.04)	60 (± 0.62)	46 (± 3.70)	70 (± 3.76)	65 (± 5.25)	**54 (± 4.15)**
EYPC/Chol 8:2	**29 (± 1.12)**	16 (± 0.68)	14 (± 0.83)	12 (± 1.43)	**59 (± 4.17)**	26 (± 3.32)	**57 (± 6.33)**
EYPC/Chol 6:4	5 (± 2.23)	6 (± 1.37)	2 (± 0.21)	5 (± 0.71)	5 (± 0.17)	5 (± 1.03)	**11 (± 4.29)**
DPPC	7 (± 1.18)	3 (± 0.19)	6 (± 0.71)	4 (± 0.27)	**14 (± 3.17)**	2 (± 0.34)	4 (± 0.33)

^a^The data represent the average of independent experiments conducted in triplicate. Values in bold indicate cases where significant release was achieved.

As cholesterol content increased, sulforhodamine B release progressively decreased, consistent with the transition to a more ordered membrane. EYPC/Chol bilayers form liquid-ordered (L_o_) phases, characterized by increased thickness, rigidity, and reduced water permeability [16]. In EYPC/Chol 8:2 LUVs, rotaxane **1**-*E* induced 29% release, more than rotaxane **1**-*Z***,** which caused only 16% release (Table 1, Figure 4a, and Supporting Information File 1, Figure S1b). Rotaxane **2** and axle **3** resulted in a release of 14% and 12%, respectively, which is comparable to **1**-*Z*. This result indicates that, in this lipid composition, membrane permeabilization increases as the size of the macrocycle increases, as observed in **1**-*E* (Suppporting Information File 1, Figure S6).

In EYPC/Chol 6:4 LUVs, release decreased to 5% for rotaxane **1**-*E*, 6% for rotaxane **1**-*Z*, 2% for rotaxane **2**, and 5% for axle **3**, indicating minimum disruption by all the tested compounds (Table 1, Figure 4a, and Supporting Information File 1, Figures S1c and S8). Similarly, in LUVs composed of pure DPPC, which adopts a highly ordered gel phase (L_β_) at room temperature [17], minimal release was observed: 7% with rotaxane **1**-*E*, 3% with rotaxane **1**-*Z*, 6% with rotaxane **2**, and 4% with axle **3** (Table 1, Figure 4a, and Supporting Information File 1, Figures S1d and S10).

Our previous molecular dynamics simulations of rotaxane **1** in POPC bilayers, a major component of EYPC [16], showed that rotaxane **1** disrupts lipid packing and promotes water accumulation within the bilayer [15]. These effects enhance membrane permeability to hydrophilic molecules such as sulforhodamine B. The simulations also suggest that the macrocycle of rotaxane **1**-*E* tends to reside closer to the lipid–water interface, promoting more water accumulation than **1**-*Z*, whose macrocycle positions deeper within the bilayer. This difference in localization likely arises from the substantial change in size and polarity of the macrocycle, and is consistent with the higher dye release observed for **1**-*E* compared to **1**-*Z* in EYPC/Chol 8:2 LUVs. By contrast, the azobenzene configuration has little effect in the more rigid EYPC/Chol 6:4 bilayers. In highly fluid pure EYPC bilayers, however, the rapid release within the first minutes of rotaxane insertion precludes a meaningful comparison between the two azobenzene configurations.

### Sulforhodamine B release from LUVs of varying lipid composition upon light irradiation

**Figure 5:**
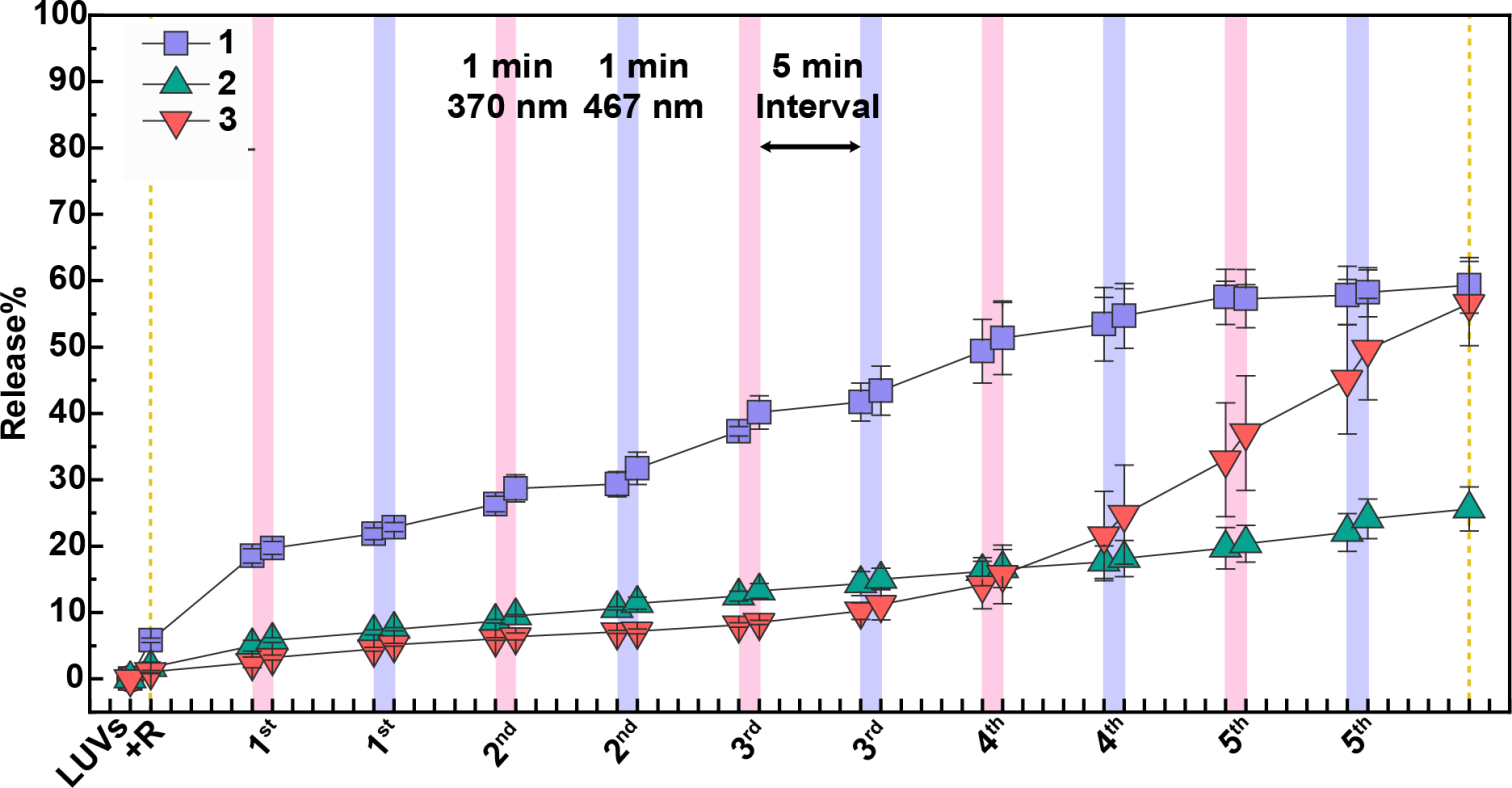
Percentage of sulforhodamine B released from EYPC/Chol 8:2 LUVs upon five irradiation cycles after the addition of rotaxane **1**, rotaxane **2**, and axle **3** in 10 mol % at 25 °C. The fluorescent emission of sulforhodamine B was measured before and after 1 minute irradiation with 370 nm light (pink strips) or 467 nm light (purple strips), and after 5 minutes post-irradiation. The LUVs are suspended in a buffer solution containing HEPES buffer (10 mM, pH 7.2) and NaCl (100 mM). Rotaxanes **1**, **2** and axle **3** (10 mol % with respect to the total lipid concentration) dissolved in DMSO were added to the LUVs. Data represents the average of independent experiments conducted in triplicate.

We then investigated whether repeated cycles of light irradiation, and thus azobenzene photoisomerization, could modulate the release of sulforhodamine B from LUVs. Rotaxane **1**-*E* was added to the vesicles, which were subsequently subjected to alternating 1 minute irradiations at 370 nm and 467 nm. Dye release was measured immediately before and after each irradiation, as well as 5 minutes post-irradiation throughout five switching cycles. The total incubation time of the LUVs with rotaxane **1** was 68 minutes, during which the cumulative irradiation time was 5 minutes at 370 nm and 5 minutes at 467 nm (Table 1, Figure 4b, Figure 5, and Supporting Information File 1, S2.3). This experimental setup enabled a direct comparison between the cumulative dye release under photoirradiation and that observed under non-irradiated conditions (70 minutes). The same irradiation protocol was applied to LUVs containing rotaxane **2** and axle **3** to evaluate the contribution of potential nonspecific effects from light exposure, such as heating, to membrane permeabilization and dye release (Supporting Information File 1, Figures S12 and S13).

In LUVs composed solely of EYPC, azobenzene photoisomerization in rotaxane **1** leads to 70% sulforhodamine B release (Table 1, Figure 4b, and Supporting Information File 1, Figure S14), comparable to the total release observed upon addition of rotaxanes **1***-E* and **1**-*Z* in the absence of light. In these highly disordered and fluid bilayers, incorporation of rotaxane **1** alone is sufficient to induce substantial membrane permeability, depleting the vesicles of most dye within the first minutes. Even minimal disruption of lipid packing appears to promote water accumulation in this system, and these dominant effects preclude assessing additional contributions from azobenzene isomerization. Likewise, upon light irradiation, rotaxane **2** and axle **3** induced dye release levels (65% and 54%, respectively) comparable to those observed in the absence of irradiation (Supporting Information File 1, Figure S14), confirming that their mere presence is sufficient to permeabilize highly fluid EYPC bilayers (Table 1 and Figure 4b).

On the other hand, LUVs composed of EYPC/Chol 8:2 exhibited the highest degree of modulation in membrane permeability and dye release in response to the photoisomerization of rotaxane **1**. After five cycles of photoisomerization, the total dye release was 59%, significantly higher than the release observed when the LUVs were exposed to either **1**-*E* or **1**-*Z* in the absence of light for 70 minutes (16–29% release) (Table 1). Notably, a greater dye release was consistently observed when rotaxane **1**-*E* was enriched than when **1**-*Z* was enriched, as indicated by the steeper slope following 467 nm irradiation cycles compared to 370 nm cycles (Figure 5). In comparison, irradiation of rotaxane **2** resulted in only 26% release, which was slightly higher than in the dark (14%) but still substantially lower than with rotaxane **1**. Nevertheless, the highest release observed with rotaxane **1** confirms that azobenzene photoisomerization is the key factor driving enhanced membrane permeability in this lipid composition. (Table 1 and Figure 4b).

Interestingly, axle **3** caused 57% dye release under light irradiation (Table 1, Figure 4b, and Figure 5), a substantial increase compared to only 12% in the dark. Because axle **3** lacks both the macrocycle and the photoswitches, this points to an alternative light-induced mechanism of membrane perturbation. Notably, the effect did not appear until after the fourth irradiation cycle. It required multiple light irradiation cycles to trigger a sudden spike in dye release, in contrast to the gradual increase observed with rotaxane **1** from the first cycle onward (Figure 5). Since such a level of release was not observed with rotaxane **2** (Figure 5), we hypothesize that this effect is unique to the unthreaded axle and is to some extent suppressed in the presence of the macrocycle. We conducted additional studies to further explore this phenomenon, which are described in a later section.

We then evaluated sulforhodamine B release from LUVs composed of EYPC/Chol 6:4. Azobenzene photoisomerization in rotaxane **1** had minimal impact, triggering only ≈5% release, with no appreciable difference between irradiated and non-irradiated vesicles (Figure 4b, Table 1, and Supporting Information File 1, Figure S17). Although azobenzene photoisomerization occurs in these more ordered bilayers (Figure 3), simulations suggest that membrane permeabilization is driven primarily by photoisomerization-induced changes in macrocycle localization within the membrane, which promote water accumulation, rather than by direct alterations in lipid packing from the azobenzene moiety itself [15]. For instance, in EYPC/Chol 8:2 bilayers, azobenzene switching likely leads to a redistribution of the rotaxane within the membrane, with the macrocycle positioned closer to the lipid–water interface in the *E* isomer and residing deeper within the bilayer in the *Z* isomer. Accordingly, this change in membrane localization influences water accumulation and disrupts lipid packing, thereby modulating membrane permeability. However, even though isomerization still occurs in the more rigid and thicker EYPC/Chol 6:4 bilayers, the macrocycle may be unable to reach the lipid–water interface or reposition effectively within this more viscous bilayer. This could be due to limited diffusion within the tightly packed lipids and/or the increased bilayer thickness, which likely hinders the structural rearrangement required for permeability modulation. Similarly, rotaxane **2** and axle **3** exhibit minimal release upon the five irradiation cycles, resulting in releases of 5% and 11%, respectively (Figure 4b, Table 1, and Supporting Information File 1, Figure S17). The slightly higher release caused by axle **3** may be attributed to the same light-induced phenomenon observed in EYPC/Chol 8:2, which suggests a different mechanism than the disruption caused by azobenzene photoisomerization in rotaxane **1** (Figure 4b and Table 1). Interestingly, membrane rigidity also appears to decrease the effect caused by the light activation of axle **3**.

**Figure 6:**
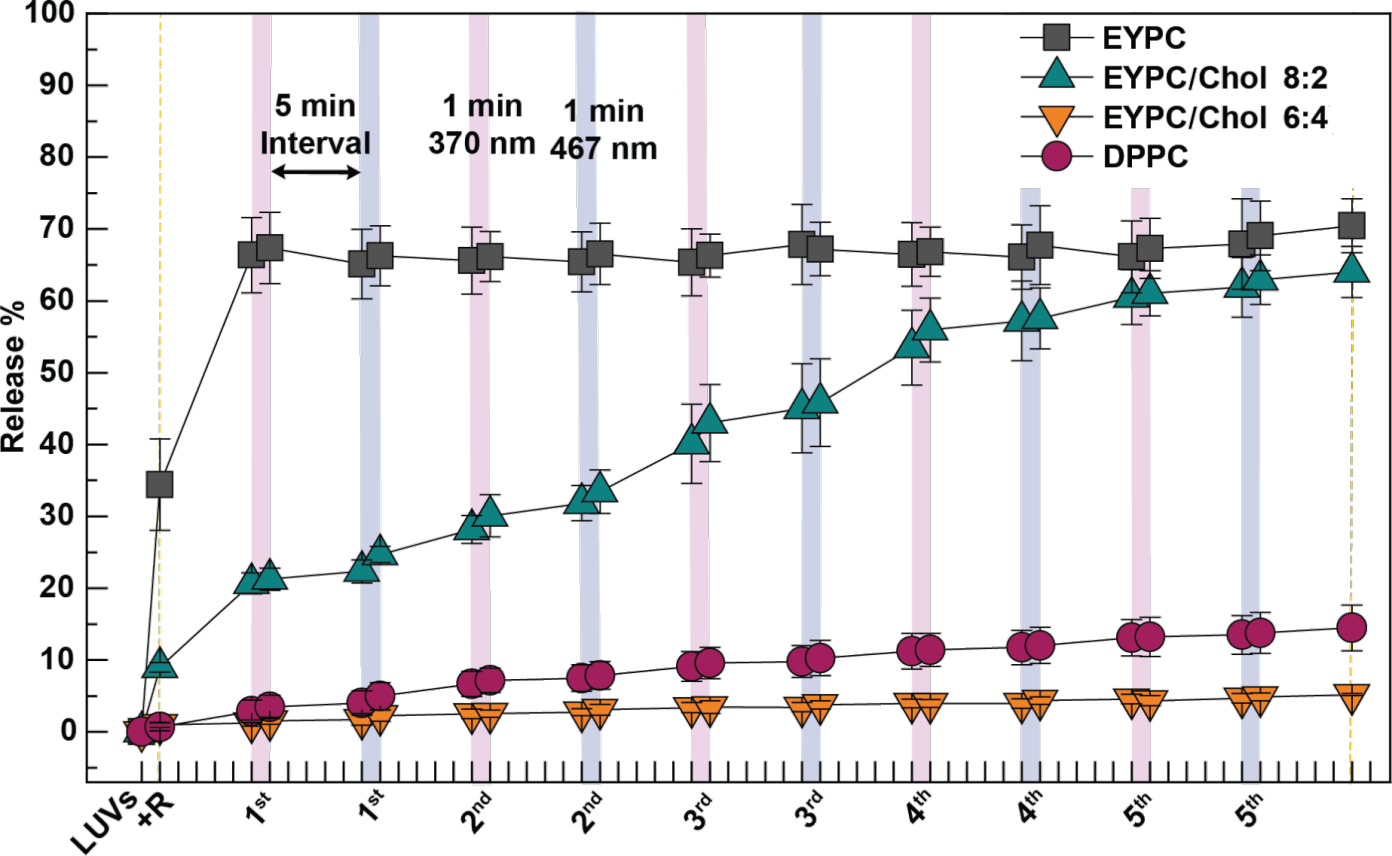
Percentage of sulforhodamine B released from LUVs containing rotaxane **1** upon five alternating light-irradiation cycles. The fluorescent emission of sulforhodamine B was measured before and after 1 minute irradiation with 370 nm light (pink strips) or 467 nm light (purple strips), and after 5 minutes post-irradiation. The sulforhodamine B (10 mM) was encapsulated in LUVs composed of different lipid compositions suspended in a solution of HEPES buffer (10 mM, pH 7.2). Rotaxane **1** (10 mol % with respect to the total lipid concentration) dissolved in DMSO was added to the LUVs. The data represent the average of independent experiments conducted in triplicate.

Lastly, in LUVs composed of DPPC, irradiation of rotaxane **1** resulted in a total sulforhodamine B release of 14%, representing an increase compared to the minimal release observed under non-irradiated conditions (3–7%) (Table 1 and Figure 6). In contrast, irradiation of rotaxane **2** and axle **3** led to only 2% and 4% release, respectively (Figure 4b, Table 1, and Supporting Information File 1, Figure S19), confirming that the enhanced permeability observed with rotaxane **1** arises from azobenzene photoisomerization rather than nonspecific effects of light. These findings indicate that azobenzene-mediated modulation of water permeability can still be achieved in DPPC bilayers. However, the overall extent of release is significantly lower than that observed in more fluid systems such as EYPC/Chol 8:2. Furthermore, the data also indicate that membrane perturbation caused by light activation of axle **3** does not occur in DPPC (Figure 4 and Supporting Information File 1, Figure S19).

Although both EYPC/Chol 6:4 and DPPC form relatively rigid membranes, differences in their chemical composition, including headgroup (such as the presence of the cholesterol OH group), acyl chain structure, and saturation, can result in different membrane mechanics, hydration, thickness, intermolecular spacing, and non-covalent interactions with the rotaxane. For example, EYPC bilayer thickness increases linearly with cholesterol concentration and can induce the formation of nanodomains [16]. Such differences in structure and mechanical properties likely explain the distinct behavior of the rotaxane in these two membranes by influencing its optimal orientation and positioning and, consequently, its ability to modulate the dye release.

Figure 6 summarizes the sulforhodamine B release from LUVs with four different lipid compositions after five cycles of rotaxane **1** photoisomerization, showing that bilayer modulation occurs in EYPC/Chol 8:2 and DPPC, with a higher release observed in the former. The corresponding data for rotaxane **2** and axle **3** are presented in Supporting Information File 1, Figures S12 and S13, respectively.

We investigated whether reducing the concentration of rotaxane **1** from 10 mol % to 5 mol % could provide better control over membrane changes in the more fluid bilayers, specifically in pure EYPC and EYPC/Chol 8:2 LUVs. In pure EYPC, both rotaxanes **1** and **2** induced similar levels of release at 5 mol % and 10 mol %, reflecting the high intrinsic permeability of this membrane and the difficulty in modulating it (Supporting Information File 1, Table S30 and Figure S21a). As expected, total release in EYPC/Chol 8:2 LUVs decreased significantly at the lower concentration of the molecules after five irradiation cycles, dropping from 59% to 28% for rotaxane **1** and from 26% to 16% for rotaxane **2** (Supporting Information File 1, Table S30 and Figure S21b). These results are consistent with our previous studies, which showed that effective membrane perturbation requires at least 10 mol % of rotaxane **1** [15].

We examined whether dye release in DPPC LUVs could be enhanced by increasing the temperature above the lipid’s phase transition temperature (*T*_m_ = 41 °C) (Supporting Information File 1, S2.4). Below *T*_m_, DPPC is in the gel phase (L_β_), where the hydrocarbon chains are highly ordered, forming rigid bilayers that limit dye release. Above *T*_m_, DPPC transitions to the liquid-crystalline phase (L_α_), characterized by more disordered, fluid chains that form thinner and more flexible bilayers [18–20]. To test the effect of phase state on release, we first equilibrated DPPC LUVs at 25 °C (below *T*_m_) and monitored sulforhodamine B leakage without light irradiation. After 10 minutes, release was minimal: 3% with rotaxane **1**-*Z*, 2% with rotaxane **2**, and 1% with axle **3** (Supporting Information File 1, Table S35 and Figure S22). When vesicles were heated to 45 °C (above *T*_m_) for a total of 10 minutes, release increased to 15%, 11%, and 11% for rotaxane **1**-*Z*, rotaxane **2**, and axle **3**, respectively, highlighting the effect of increased membrane fluidity (Supporting Information File 1, Table S35 and Figure S22). We did not extend these experiments to 70 minutes, as in the other studies (Table 1), because rotaxane **1**-*Z* would convert to **1**-*E* over that timescale. Nevertheless, the results clearly show that heating above *T*_m_ enhances release.

We next evaluated the effect of light irradiation on rotaxane **1** in DPPC. At 25 °C, the initial addition of **1**-*Z* resulted in only a 3% release after 5 minutes. Irradiation at 467 nm for one minute converted **1**-*Z* to **1**-*E*, after which release increased slightly to 5% over the next 10 minutes, confirming the minimal impact of one photoisomerization in the gel phase. Consistent with this, the previous study (Table 1) showed 14% release after five photoisomerization cycles below *T*_m_. In contrast, when vesicles were heated to 45 °C and **1**-*Z* was irradiated for one minute to form **1**-*E*, the release increased from 15% to 20% after 9 minutes (to keep the study length to 15 minutes consistent), approximately four times higher than the release after similar irradiation at 25 °C (5%) (Supporting Information File 1, Table S35 and Figure S22). This confirms that **1**-*E* is more effective at permeabilizing the bilayer than **1**-*Z*, and that this effect is further enhanced above *T*_m_, when the bilayer is more fluid.

### Light-induced sulforhodamine B release by axle **3**

**Figure 7:**
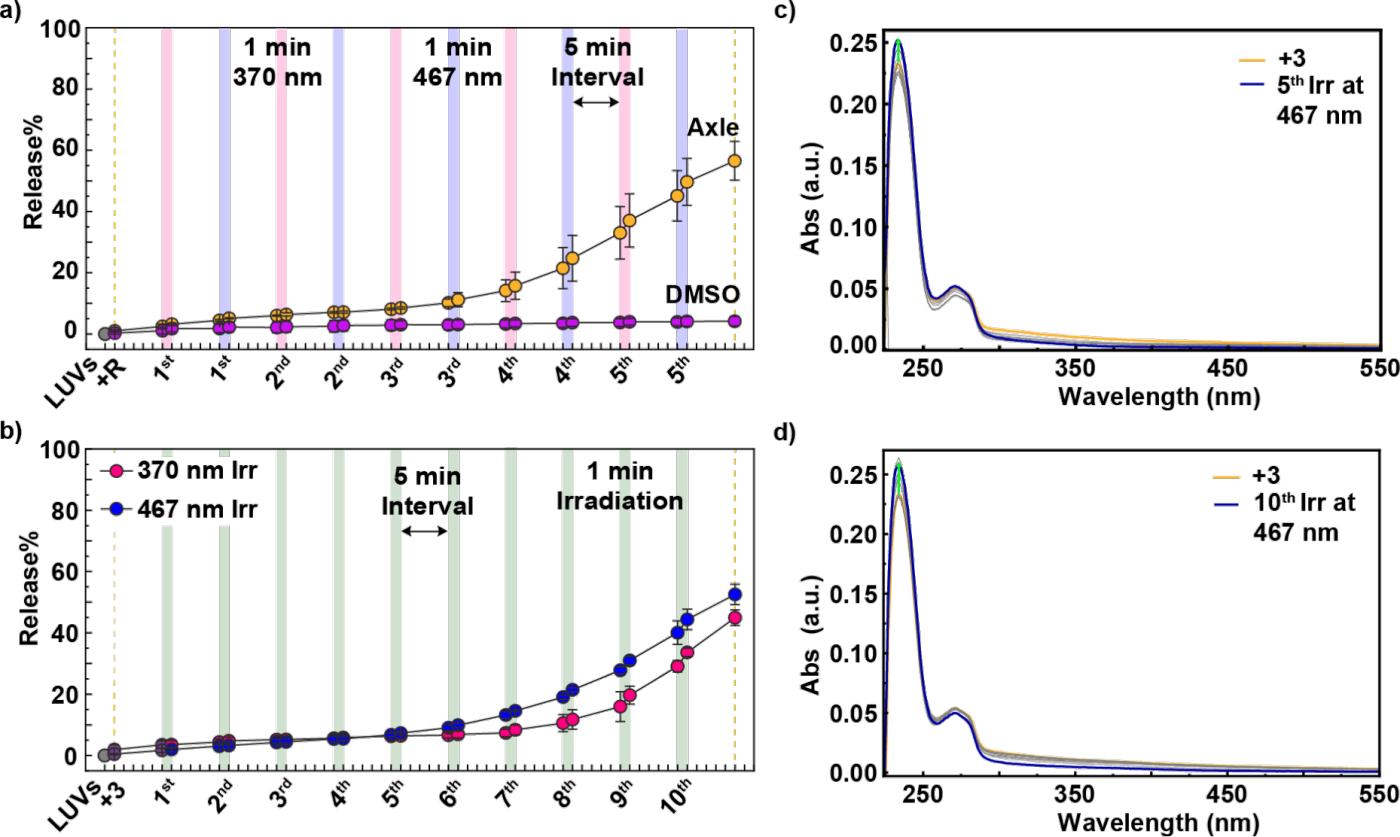
Evaluation of effect of axle **3** upon light exposure. a) Percentage of sulforhodamine B released from EYPC/Chol 8:2 LUVs containing axle **3** or DMSO as a negative control upon five alternating light-irradiation cycles. The fluorescent emission of sulforhodamine B was measured before and after 1 minute irradiation with 370 nm light (pink strips) or 467 nm light (blue strips), and after 5 minutes post-irradiation. b) Percentage of sulforhodamine B released from LUVs containing axle **3** upon ten light irradiations at either 370 nm or 467 nm. Green strips represent one minute of irradiation at either wavelength. c) Changes in absorbance of EYPC/Chol 8:2 LUVs containing axle **3** upon five irradiation cycles. The absorbance was measured before and after 1 minute irradiation at 370 nm light and 467 nm light alternately, where the blue trace corresponds to the absorption after the last irradiation at 467 nm. d) Changes in absorbance of EYPC/Chol 8:2 LUVs containing axle **3** upon ten irradiations at only 467 nm light. The sulforhodamine B (10 mM) was encapsulated in LUVs composed of 8:2 EYPC/Chol ratio suspended in a solution of HEPES buffer (10 mM, pH 7.2). Axle **3** (10 mol % with respect to the total lipid concentration) dissolved in DMSO was added to the LUVs. LUVs without sulforhodamine B were used for UV–vis spectroscopy studies.

The unusual membrane perturbation caused by light irradiation in the presence of axle **3** is puzzling, as it lacks azobenzene photoswitches or other apparent photoactive units with strong light absorption at 370 or 467 nm. To investigate this, we compared dye release from EYPC/Chol 8:2 LUVs containing axle **3** with vesicles treated with the same amount of DMSO but no axle over five irradiation cycles alternating 370 nm and 467 nm light. As seen in Figure 7a, the release occurs only in vesicles containing axle **3**, ruling out nonspecific effects such as membrane degradation from direct light exposure or solvent heating (HEPES buffer and small amounts of DMSO). We also monitored the absorption spectra of LUVs suspended in HEPES buffer in the presence of DMSO and observed no changes (Supporting Information File 1, Figure S23), confirming that the liposomes did not undergo chemical degradation in the absence of axle **3**. In contrast, LUVs containing axle **3** showed minor changes in their absorption spectra: the absorption tail between 300–500 nm decreased, while the band at 240 nm increased (Figure 7c). Notably, these absorption changes were irreversible, unlike those observed in LUVs containing rotaxane **1** (Figure 3) [15], which reflect the reversible photoswitching of the azobenzene. When irradiating axle **3** in solution using DMSO as solvent, we observed similar irreversible changes in its absorption, indicating a degradation of the axle upon light exposure (Supporting Information File 1, Figure S23d).

We then investigated which wavelength was responsible for activating axle **3**. EYPC/Chol 8:2 LUVs containing axle **3** were irradiated ten times with either 370 nm or 467 nm light, applied separately. Each experiment consisted of ten irradiation cycles, with one minute of light exposure followed by a five-minute dark period (Figure 7b). Irradiation at 467 nm resulted in slightly higher dye release compared to 370 nm, accompanied by similar changes in absorption spectra (Figure 7c and 7d).

The most likely photoactive unit in axle **3** is the triazolium group. However, triazoles typically absorb in the 200–220 nm region (π→π* transitions) [21], and only extend into the longer UV and near-visible range when conjugated to chromophores or electron-rich groups [22–24], which is not the case here. Despite the lack of significant absorption at 370–467 nm, dye release was clearly observed, but only in vesicles containing EYPC. Possible explanations include local membrane heating through non-radiative decay of axle **3**, disruption of lipid packing via photolysis of axle **3**, or the formation of reactive oxygen species (ROS) that may react with the unsaturated acyl chains of EYPC lipids; DPPC, by contrast, lacks double bonds. Further studies will be required to clarify this unexpected phenomenon.

Nevertheless, this effect was largely suppressed in rotaxane **2** (Supporting Information File 1, Figure S23e), suggesting that the macrocycle may shield the triazolium or other axle groups, and likely also contributes to the absence of this effect in rotaxane **1**. Furthermore, comparison of the absorption spectra of all compounds at the same concentration shows that **1**-*E* and **1**-*Z* exhibit much stronger absorption at 370 and 467 nm than rotaxane **2** and axle **3**, which absorb almost negligibly (Supporting Information File 1, Figure S23f). Thus, even if some photoactivation of the axle occurs in rotaxane **1**, the dominant absorption of the azobenzene units ensures that most of the light energy is directed toward azobenzene photoisomerization. Additionally, their different release kinetics provide more insights: rotaxane **1** causes gradual, stepwise release beginning from the first irradiation cycle due to efficient azobenzene photoisomerization, whereas axle **3** requires multiple cycles before a sudden release occurs (Figure 7a), indicating a less efficient and irreversible process. Moreover, microscopy imaging shows reversible changes to membrane tension in response to azobenzene photoswitching, which is supported by MD simulations [15]. Therefore, rotaxane **1** and axle **3** permeabilize membranes through distinct mechanisms.

### Effect of macrocycle localization

**Figure 8:**
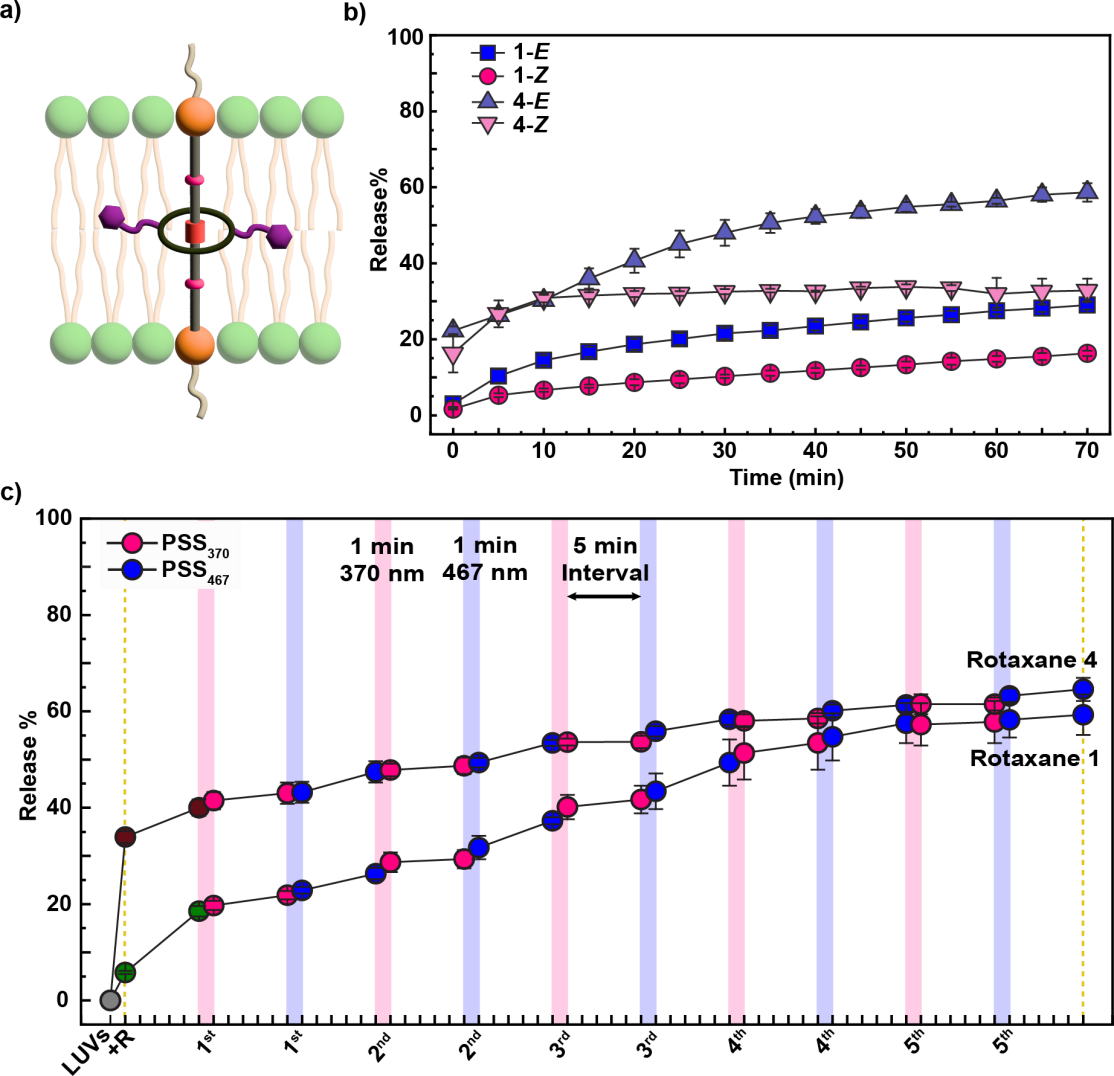
a) Illustration of rotaxane **4** in its preferred orientation within a lipid bilayer; percentage of sulforhodamine B released from LUVs containing rotaxanes **1** and **4**; b) without light irradiation, and c) upon five alternating light-irradiation cycles. The fluorescent emission of sulforhodamine B was measured before and after 1 minute irradiation with 370 nm light (pink strips) or 467 nm light (blue strips), and after 5 minutes post-irradiation. The sulforhodamine B (10 mM) was encapsulated in LUVs composed of 8:2 EYPC/Chol ratio suspended in a solution of HEPES buffer (10 mM, pH 7.2). Rotaxanes **1** and **4** (10 mol % with respect to the total lipid concentration) dissolved in DMSO were added to the LUVs.

The macrocycle with the azobenzene photoswitches in rotaxane **1** can translate and be localized in either of the ammonium groups or in the *N*-methyltriazolium (MTA) unit at a certain point in time; thus, the azobenzene photoisomerization could cause membrane perturbations at different membrane depths. Hence, we investigated whether a more localized macrocycle would have a similar effect on the dye release (Figure 8a). Accordingly, we deprotonated the ammonium sites with sodium hydroxide (Supporting Information File 1, S4), obtaining rotaxane **4**, where the macrocycle exclusively locates at the MTA unit (Supporting Information File 1, Figure S25), so it is expected to be localized at the center of the hydrophobic region of the bilayer. This enables us to investigate how the switching of azobenzene units affects the hydrophobic core of the bilayer.

We confirmed the efficient photoisomerization and photoreversibility of rotaxane **4** in solution over 20 cycles using UV–vis spectroscopy (Supporting Information File 1, Figure S29). Similar to rotaxane **1** [15], the *Z*→*E* thermal isomerization of rotaxane **4** occurs over 85 minutes at 40 °C (Supporting Information File 1, Figure S30). This confirmed that the azobenzene operation is not affected by being located at the MTA unit. We then studied the photoswitching of rotaxane **4** in EYPC/Chol 8:2 LUVs (Supporting Information File 1, Figure S31), observing a similar photoswitching behavior to that of rotaxane **1** (Figure 3), which confirms the photoreversibility of rotaxane **4** in the membranes.

Next, we studied sulforhodamine B release induced by adding either **4**-*E* or **4**-*Z* to EYPC/Chol 8:2 LUVs without light irradiation. Rotaxane **4** caused significantly more release than **1**, with 59% for **4**-*E* versus 29% for **1**-*E*, and 33% for **4**-*Z* versus 16% for **1**-*Z* after 70 minutes of exposure (Figure 8b). Both isomers, **4**-*E* and **4**-*Z*, caused substantial release immediately upon addition. For **4**-*Z*, release occurred mainly within the first 10 minutes and then plateaued for the following hour, indicating that the initial interaction drives most of the perturbation. In contrast, **4**-*E* continued to promote release over time, reflecting larger and sustained membrane disruption. Comparing isomers, the difference in permeabilization is larger for **4**-*E* versus **4**-*Z* than for **1**-*E* versus **1**-*Z*, suggesting that **4** may allow more distinctive control over membrane behavior through its isomer-dependent effects.

The pronounced release upon insertion of **4** can be rationalized by the relative position of the macrocycle along the axle. In **4**, the macrocycle is positioned at the center, so when the molecule inserts into the bilayer, the bulky ring encounters the membrane early. This central placement likely causes greater steric mismatch and local disruption of lipid packing, resulting in higher permeability. By contrast, in **1**, the macrocycle is closer to one end (at an ammonium site), allowing the linear portion of the axle to insert first, causing less immediate perturbation. This smoother initial insertion delays the disruptive impact of the ring, producing lower early-stage release for **1**.

We also evaluated whether rotaxane **4** can modulate the bilayer under light exposure (Figure 8c). Upon addition, **4** caused substantial dye release (34%), reaching 65% after five switching cycles, slightly higher than **1** under the same conditions (59%). Considering the change in release relative to initial incorporation (Δ release), **1** caused more release upon irradiation. Rotaxane **4** starts at 34%, yielding a Δ release of 31% (65–34) due to reversible azobenzene isomerizations, whereas **1** starts at 6%, resulting in a larger Δ release of 53% (59–6). This indicates that while **4** induces a strong initial perturbation due to early membrane rearrangement, the release driven by several cycles of azobenzene photoswitching is higher in **1**, potentially because both isomers, **1**-*E* and **1**-*Z*, can contribute to leakage, whereas in **4**, after initial insertion, mainly **4**-*E* drives permeability changes, and release is minimal in **4**-*Z*. Nevertheless, the precise reasons for the difference between **4**-*E* and **4**-*Z* require follow-up studies at the molecular level to provide further insights into the interactions of rotaxane **4** with the lipid bilayer.

## Conclusion

In this study, we demonstrated that the photoisomerization of rotaxane **1** remains efficient and reversible across multiple membrane environments, including LUVs composed of EYPC, EYPC/Chol mixtures, and DPPC. UV–vis spectroscopy confirmed robust photoswitching over ten irradiation cycles with minimal fatigue, independent of bilayer composition. However, the membrane's physical properties strongly influenced the isomerization effect of the azobenzene in modulating bilayer permeability.

The highest sulforhodamine B release was observed in EYPC membranes. In these highly fluid and loosely packed bilayers, the insertion of rotaxane **1** alone was sufficient to induce substantial dye release within a few minutes, with azobenzene photoisomerization providing no additional release. This behavior aligns with our previous molecular dynamics simulations, which showed that rotaxane **1** disrupts lipid packing and promotes water accumulation within the bilayer [15]. These effects are driven by the position of the macrocycle in the bilayer, which is influenced by azobenzene isomerization. However, in the highly disordered EYPC membranes, the macrocycle appears to retain sufficient mobility regardless of the azobenzene configuration, enabling it to reach the lipid–water interface and trigger membrane permeabilization in both isomeric states.

In contrast, LUVs composed of EYPC/Chol 8:2 exhibited a strong light-dependent response, with dye release increasing from 16–29% in the dark to ≈59% after five photoisomerization cycles. This composition represents a sweet spot in membrane thickness and order, fluid enough to accommodate rotaxane reorganization, yet structured enough to allow modulation via azobenzene switching. Increasing the cholesterol content further (EYPC/Chol 6:4) significantly reduced baseline and light-triggered dye release. This is likely due to increased membrane thickness and rigidity, as well as other changes in the membrane that may hinder the macrocycle's ability to reach the lipid–water interface or reposition effectively within the bilayer upon azobenzene isomerization.

Moreover, azobenzene photoswitching was still capable of enhancing dye release in gel-phase (DPPC) bilayers compared to both the non-irradiated conditions and the control rotaxane lacking azobenzene and unthreaded axle. This suggests that some degree of macrocycle repositioning, and therefore membrane perturbation, is still possible in these highly ordered, rigid membranes.

Surprisingly, irradiation of axle **3** also triggered the release of sulforhodamine B. Initial studies suggest a photochemical reaction of the axle, which is suppressed when the macrocycle is threaded. Furthermore, the distinct release kinetics and absorption profiles of rotaxane **1** and axle **3**, combined with the reversible photoswitching of rotaxane **1** versus the irreversible photochemical process in axle **3**, and the diminished effect in rotaxane **2**, indicate that release in these systems occurs through different mechanisms.

Moreover, our data indicate that the localization of the macrocycle along the axle can lead to variations in release. For rotaxane **4**, where the macrocycle is static at the center of the axle, significant release occurs during the insertion phase, likely due to membrane reorganization within the first minutes, but azobenzene photoswitching results in lower release than in rotaxane **1**, where the macrocycle could move within the axle.

This study offers valuable initial insights into the benefits and potential drawbacks of azobenzene-decorated rotaxanes for modulating membrane structure and dynamics across bilayers with varying compositions for cargo release. These findings inform which types of membranes, and consequently, which biological processes could be most receptive to modulation by such molecular systems. Looking ahead, more detailed molecular-level studies will deepen mechanistic understanding and, together with structural refinement, guide the design of rotaxanes with enhanced membrane localization, isomer-dependent responsiveness, and overall functional efficacy. Such optimized systems have significant potential for various applications, including controlled drug delivery and the targeted modulation of membrane properties in live cells for biotechnological and therapeutic use.

## Experimental

All glassware used in chemical reactions was dried in an oven overnight before use. Reactions were conducted under a nitrogen atmosphere unless otherwise noted in the protocols. ACS-grade dichloromethane was employed for liquid–liquid extractions. Standard buffers, purchased from Sigma-Aldrich, were used for biophysical assays. Liposomes were prepared with egg yolk phosphatidylcholine (EYPC), Dipalmitoylphosphatidylcholine (DPPC), and cholesterol, all obtained from Avanti Lipids. All buffer solutions were prepared using Milli-Q Water from a Barnstead™ Water Purification System. Molecular biology grade dimethyl sulfoxide was used for stock solutions of the compounds, while chloroform was used for cholesterol and lipid stock solutions.

^1^H NMR spectra were recorded using Bruker AV III 400 and Bruker AV III 500 spectrometers. ^13^C NMR was conducted on a Bruker AV III 500 spectrometer. All chemical shifts (δ) are reported in ppm, referencing residual non-deuterated solvent signals (acetonitrile-*d*_3_: δ_H_ = 1.94 ppm, δ_C_ = 1.32 ppm). Coupling constant (*J*) values are in hertz (Hz). Resonance multiplicities are described as s (singlet), app s (apparent singlet), d (doublet), t (triplet), q (quartet), m (multiplet), and br (br). Mass spectrometry (MS) was conducted at the Louisiana State University Mass Spectrometry Facility using the Waters Synapt XS ESI Q-TOF instrument. The main peaks and clusters are reported in *m*/*z* units, with M^+^ representing the molecular ion and the corresponding intensities in percent.

UV–vis studies were carried out using an Agilent Cary 5000 UV–vis–NIR spectrophotometer. Photoswitching was achieved through light irradiation from Kessil’s second-generation 370 nm LED and first-generation 467 nm LED. Fluorescence measurements were taken with an FS5 spectrofluorometer from Edinburgh Instruments.

## Supporting Information

File 1 Additional experimental data.

## Data Availability

All data that supports the findings of this study is available in the published article and/or the supporting information of this article.
