# Supplementary material for: Effect of a photoswitchable rotaxane on membrane permeabilization across lipid compositions
Source: Beilstein J Org Chem. 2025 Nov 11;21:2498–512. doi: 10.3762/bjoc.21.192 (PMC12621636; doi:10.3762/bjoc.21.192)
Supplement: File 1 — Additional experimental data. [file Beilstein_J_Org_Chem-21-2498-s001.pdf]

BEILSTEIN JOURNAL OF ORGANIC CHEMISTRY

Supporting Information

for

Effect of a photoswitchable rotaxane on membrane permeabilization across lipid compositions

Udyogi N. K. Conthagamage, Lilia Lopez, Zuliah A. Abdulsalam and Víctor García-López

Beilstein J. Org. Chem. **2025,** *21,* 2498–2512. doi:10.3762/bjoc.21.192

Additional experimental data

License and Terms: This is a supporting information file under the terms of the Creative Commons Attribution License (https://creativecommons.org/licenses/by/4.0). Please note that the reuse, redistribution and reproduction in particular requires that the author(s) and source are credited and that individual graphics may be subject to special legal provisions.
The license is subject to the *Beilstein Journal of Organic Chemistry* terms and conditions: (https://www.beilstein-journals.org/bjoc/terms)

Table of contents

	List of abbreviations…………………………………………………………		S1
S1.	Photoswitching studies in lipid bilayers of different compositions…………		S2
	S1.1.	Preparation of LUVs……………………………………..…………….	S2
	S1.2.	Photoswitching reversibility in LUVs monitored by UV–vis spectroscopy……………………………………………….……….	S3
S2.	Photoswitching studies in bUsing sulforhodamine B encapsulated LUVs……………………………………………………………………...……		S3
	S2.1.	Preparation of sulforhodamine B encapsulated LUVs………………	S3
	S2.2	Time-dependent release of sulforhodamine B from LUVs without light irradiation………………………………………………………………	S4
		S2.2.1. Dye release studies in EYPC LUVs………………………..	S7
		S2.2.2. Dye release studies in EYPC/Chol 8:2 LUVs……………	S11
		S2.2.3. Dye release studies in EYPC/Chol 6:4 LUVs……………	S15
		S2.2.4. Dye release studies in DPPC LUVs………………………..	S19
	S2.3.	Release of sulforhodamine B from LUVs with light irradiation……..	S23
		S2.3.1. Dye release studies in EYPC LUVs………………………..	S25
		S2.3.2. Dye release studies in EYPC/Chol 8:2 LUVs……………	S30
		S2.3.3. Dye release studies in EYPC/Chol 6:4 LUVs……………	S34
		S2.3.4. Dye release studies in DPPC LUVs………………………..	S39
		S2.3.5. Effect of varying rotaxane **1** concentrations on sulforhodamine B release from LUVs with light irradiation……………………………………………………….	S44
	S2.4.	Temperature-dependent sulforhodamine B release from DPPC LUVs……………………………………………………………..…	S50
		S2.4.1. Sulforhodamine B release from DPPC LUVs at 25 °C without light irradiation……………………………………..	S50
		S2.4.2. Sulforhodamine B release from DPPC LUVs at 25 °C with light irradiation…………………………………………………	S50
		S2.4.3. Sulforhodamine B release from DPPC LUVs at 45 °C without light irradiation………………………………………	S51
		S2.4.4. Sulforhodamine B release from DPPC LUVs at 45 °C with light irradiation…………………………………………………	S51

S3.	Investigation of the behavior of axle **3** in LUVs upon light irradiation………	S56
S3.1.	Sulforhodamine B release from EYPC/Chol 8:2 LUVs with 370 nm and 467 nm irradiations……………………………………………..	S56
S3.2.	Changes in absorbance of axle **3** over light irradiation……………..	S60
S4.	Characterization and investigation of rotaxane **4**……………….……………	S63
S4.1.	Synthetic scheme of rotaxane **4**……………………………………….	S63
S4.2.	Synthetic procedure of rotaxane **4**…………………………………….	S64
S4.3.	NMR and mass spectra of rotaxane **4**………………………………..	S66
S4.4.	Photoswitching studies in solution by UV–vis spectroscopy………..	S67
S4.5.	*Z*→*E* Thermal isomerization in solution………………………………..	S68
S4.6.	Photoswitching reversibility in lipid bilayers monitored by UV–vis spectroscopy…………………………………………………………...…	S69
S4.7.	Time-dependent release of sulforhodamine B from LUVs without light irradiation……………………………………………………………...	S70
S4.8.	Release of sulforhodamine B from LUVs with light irradiation……..	S71
S5.	References ……………………………………………..........	S73

List of abbreviations

Chol	Cholesterol
DMSO	Dimethyl sulfoxide
DPPC	Dipalmitoylphosphatidylcholine
EYPC	Egg yolk phosphatidylcholine
HEPES	Hydroxyethylpiperazine ethane sulfonic acid
LED	Light emitting diodes
LUVs	Large unilamellar vesicles
PSS	Photostationary state
UV–vis	Ultraviolet–visible

S1. Photoswitching studies in lipid bilayers of different compositions

S1.1. Preparation of LUVs

LUVs with different compositions were prepared according to our previously reported procedure [1].The volumes taken from each lipid stock and cholesterol stock are shown in Table S1. First, the respective lipid (50 mg/mL in chloroform) and/ or cholesterol (25 mg/mL in chloroform) were mixed in a 10 mL glass vial to make the respective ratios. This chloroform mixture was dried under vacuum with continuous rotation of the vial to make a transparent thin film of lipids on the wall of the glass vial. The transparent thin film was kept in a high vacuum for an additional 5 h to remove all traces of chloroform. Next, the dry thin film was hydrated with 500 µL HEPES buffer (500 µL,10 mM, pH 7.2). Then, the lipid mixture was vortexed 6–7 times occasionally over 2 h, giving a suspension followed by 12–13 freeze–thaw cycles. The cycles consisted of freezing with liquid nitrogen and melting with water at 80 °C to disintegrate potential multilamellar vesicles. For the preparation of DPPC LUVs, a 50 °C water bath was used during the freeze–thaw process step. After this step, the lipid suspension was extruded 31 times (must be an odd number) using a mini extruder with a polycarbonate membrane (200 nm pore size) from Avanti Polar Lipids. Only to extrude DPPC LUVs, the extruder was heated to 45–50 °C. Then the extruded LUVs mixture was further purified by a size exclusion PD-10 column (pre-packed with Sephadex™ G-25M) to obtain LUVs (the final lipid concentration was 25 mM in HEPES buffer, assuming 100 % lipid regeneration).

Table S1. The volumes taken from different lipid stocks and cholesterol stocks to prepare LUVs with different fluidities

LUV Composition	Volume (µL)	
	**Lipid (50 mg mL^{-1})**	**Cholesterol (25 mg mL^{-1})**
EYPC	192	0
EYPC/Chol 8:2	154	39
EYPC/Chol 6:4	115	77
DPPC	184	0

S1.2. Photoswitching reversibility in LUVs monitored by UV–vis spectroscopy

The photoswitching of rotaxane **1** in large unilamellar vesicles (LUVs) made from different lipid compositions was studied, specifically LUVs of pure EYPC, EYPC/cholesterol at 8:2, and 6:4 ratios, as well as pure DPPC. First, HEPES buffer (2970 µL, 10 mM, pH 7.2) was added to a quartz cuvette, followed by the addition of LUVs (24 µL, with a lipid content of 25 mM) in HEPES buffer. Next, rotaxane **1** in DMSO (6 µL, 10 mM) was added to the cuvette, and the solution was gently mixed. For the blank, a separate cuvette was prepared by mixing DMSO (6 µL), LUVs (24 µL, with a lipid content of 25 mM in HEPES buffer), along with HEPES buffer (2970 µL, 10 mM, pH 7.2). The initial absorption spectrum of each sample (without any light irradiation) was measured at 25 °C. Each sample was then irradiated at 370 nm for 1 minute to reach the photostationary state ($PSS_{370\ nm}$), and the respective absorption spectrum was recorded. Following this, each sample was irradiated at 467 nm for 1 minute to reach the $PSS_{467\ nm}$, and the corresponding absorption spectrum was recorded. The light irradiation was performed using a Kessil LED positioned 8 cm away from the center of the 3.5 mL quartz cuvette. All samples were alternately irradiated at 370 nm and 467 nm for 5 minutes each to complete ten photoswitching cycles. After each irradiation step, the absorption spectrum was recorded, and the photoreversibility graphs were plotted at the absorbance value given at 365 nm for each step.

S2. Photoswitching studies in bilayers using sulforhodamine B encapsulated LUVs

S2.1. Preparation of sulforhodamine B encapsulated LUVs

LUVs with varying lipid compositions were prepared using a modified procedure outlined in Table S1, which details the volumes taken from each lipid and cholesterol stock. Initially, the respective lipids (50 mg/mL in chloroform) and/or cholesterol (25 mg/mL in chloroform) were combined in a 10 mL glass vial to create the desired ratios. This chloroform mixture was then dried under vacuum while continuously rotating the vial to form a transparent thin film of lipids on the wall of the glass vial. To ensure the complete removal of chloroform traces, the transparent thin film was kept in high vacuum for an additional

5 hours. Next, the dry thin film was hydrated with 500 μL of intravascular buffer (10 mM HEPES buffer, 10 mM NaCl, 10 mM sulforhodamine B, pH 7.2). The lipid mixture was vortexed 6 to 7 times occasionally over 2 hours to create a suspension. This step involved undergoing 12 to 13 freeze–thaw cycles, alternating between freezing in liquid nitrogen and thawing in water at 80 °C, to break apart any potential multilamellar vesicles. For the preparation of DPPC LUVs, a water bath maintained at 50 °C was used during the freeze–thaw process. Afterward, the lipid suspension was extruded 31 times (using an odd number, as required) through a mini extruder equipped with a polycarbonate membrane with a 200 nm pore size, sourced from Avanti Polar Lipids. For the extrusion of DPPC LUVs, the extruder was heated to 45–50 °C. Subsequently, size exclusion chromatography was performed to eliminate unencapsulated sulforhodamine B, using a PD-10 column prepacked with Sephadex G-25 M, with a buffer solution (10 mM HEPES, 100 mM NaCl, pH 7.2) as the eluting solution. This process yielded a final concentration of liposomes containing sulforhodamine B at 12.5 mM in 1 mL, assuming 100% lipid recovery.

S2.2. Time-dependent release of sulforhodamine B from LUVs without light irradiation

To evaluate the individual effects of the *E*- and *Z*-configurations of rotaxane **1** on the permeability of large unilamellar vesicles (LUVs), the percentage of sulforhodamine B released at 5-minute intervals over a total duration of 70 minutes was measured. This period is approximately equivalent to the time required to assess the sulforhodamine B release over five cycles of light irradiation. Initially, 2946 μL of an extravesicular buffer (10 mM HEPES, 100 mM NaCl, pH 7.2) with 48 μL of LUVs encapsulating sulforhodamine B (12.5 mM) were combined in a quartz cuvette. The mixture was placed in the fluorometer chamber and stirred at 150 rpm for 2 minutes to ensure homogeneity. We then recorded the emission spectrum from 575 nm to 750 nm, using an excitation wavelength of 565 nm. All fluorescence studies were conducted at 25 °C, with the mixture continuously stirred at 150 rpm during the emission spectrum recordings. Next, 6 μL of a DMSO solution containing 10 mM rotaxane **1**-*E* was added to the cuvette, and the emission spectrum was recorded. After that, the fluorescence was measured every 5 minutes for 70 minutes. Finally, Triton X-100 was added to the cuvette and stirred for

8 minutes to lyse the liposomes, followed by another recording of the emission spectrum. The percentage of sulforhodamine release was calculated according to the following equation,

$$\%\,Sulforhodamine\ B\ release\ =\ 100\ \times\ \frac{(F_t - F_0)}{(F_{max} - F_0)} \qquad \textbf{S1}$$

where F_t is the maximum fluorescence emission after adding the compound, F_0 is the maximum fluorescence emission before adding the compound, and F_{max} is the maximum fluorescence measured after adding Triton X-100. This procedure was followed for **1**-*E* and **1**-*Z* separately with all lipid compositions, and each isomer was tested three different times.

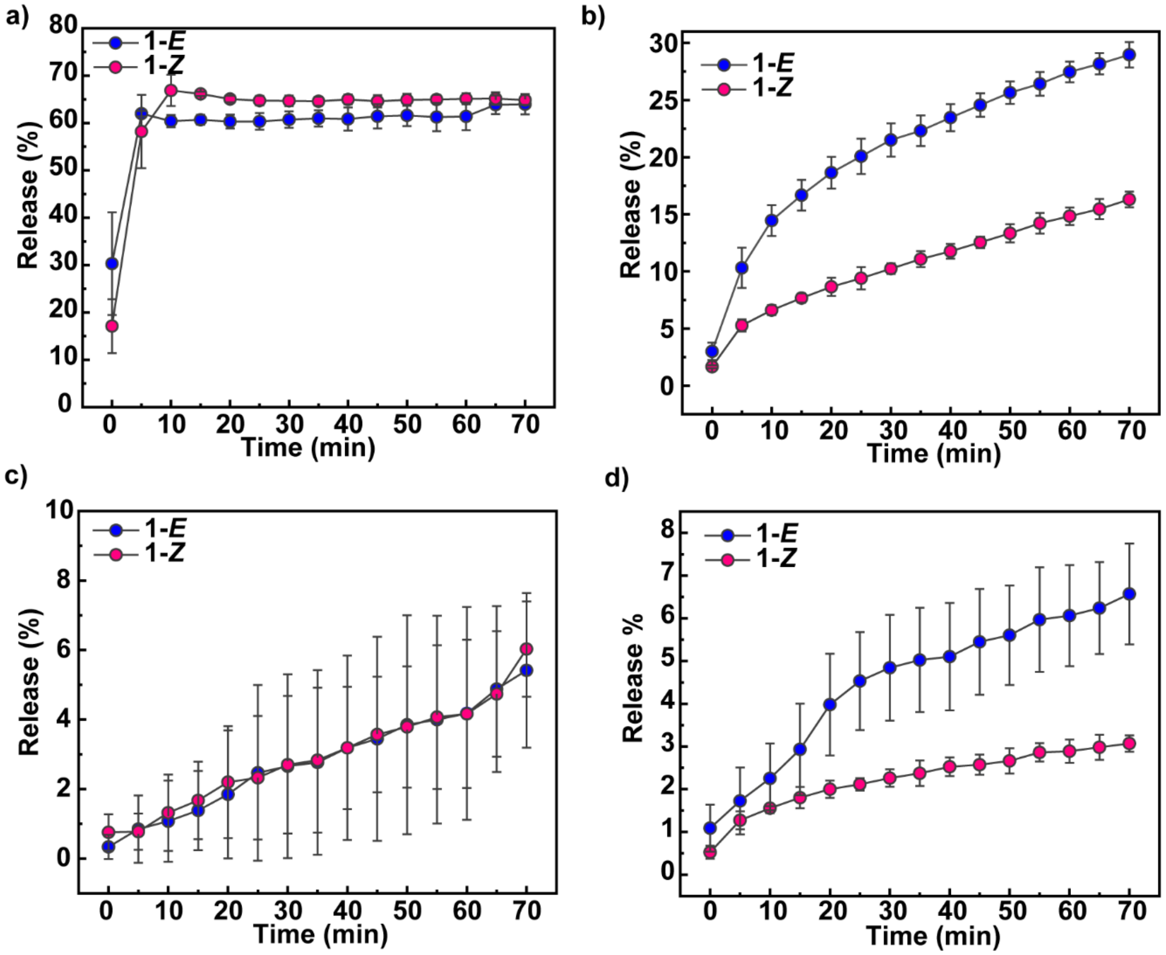

Figure S1: Percentage of sulforhodamine B released from rotaxane **1-***E* and **1-***Z***,** without light irradiation in LUVs composed of **a)** EYPC, **b)** EYPC/Chol 8:2, **c)** EYPC/Chol 6:4, and **d)** DPPC

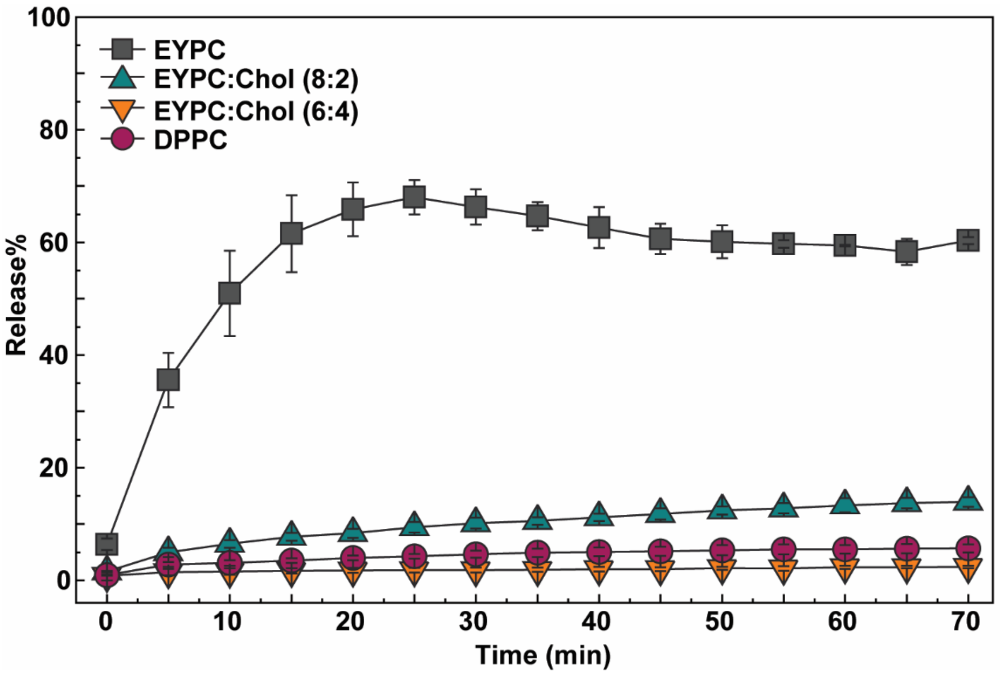

Figure S2: Percentage of sulforhodamine B released from LUVs containing rotaxane **2** without light irradiation. The sulforhodamine B (10 mM) was encapsulated in LUVs composed of different lipid/cholesterol ratios suspended in a solution of HEPES buffer (10 mM, pH 7.2).

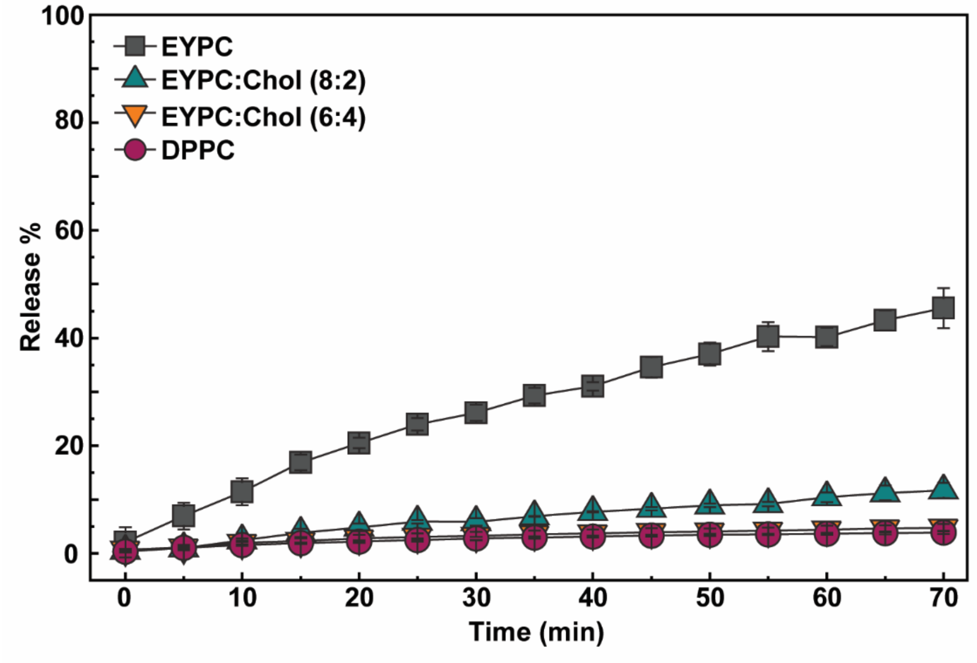

Figure S3: Percentage of sulforhodamine B released from LUVs containing rotaxane axle **3,** without light irradiation. The sulforhodamine B (10 mM) was encapsulated in LUVs composed of different lipid/cholesterol ratios suspended in a solution of HEPES buffer (10 mM, pH 7.2).

S2.2.1. Dye release studies in EYPC LUVs

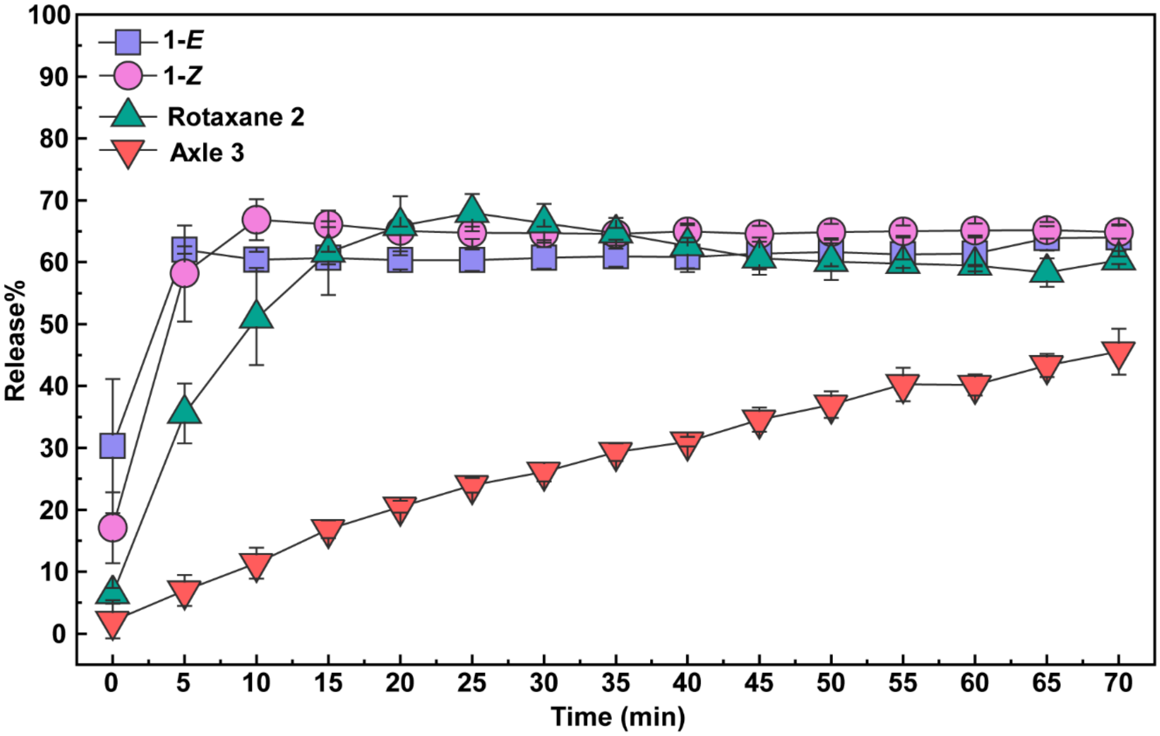

Figure S4. Percentage of sulforhodamine B released over time after adding **1-*E*, 1-*Z*,** rotaxane **2** or axle **3** to EYPC LUVs at 25 °C. The LUVs are suspended in a buffer solution containing HEPES buffer (10 mM, pH 7.2) and NaCl (100 mM).

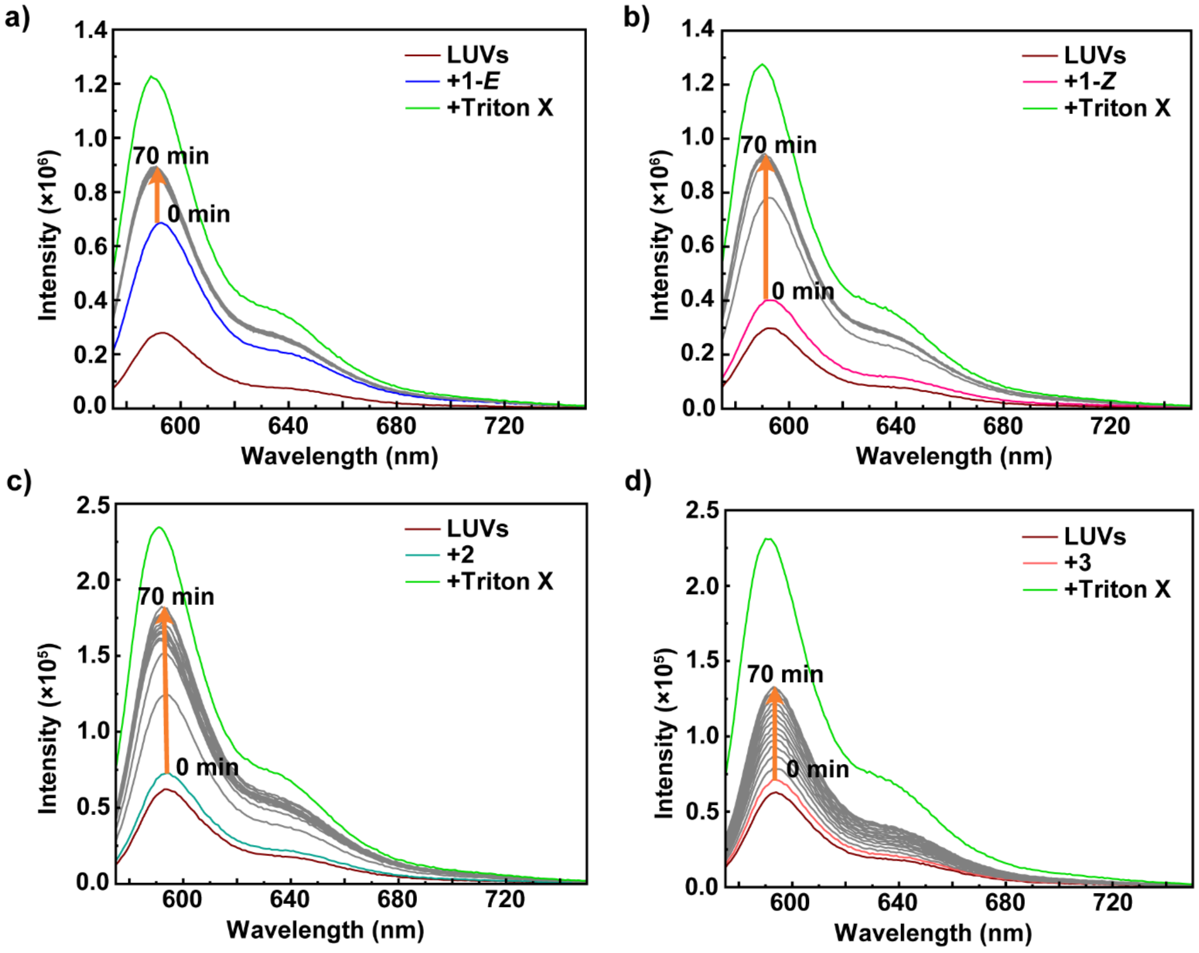

Figure S5. Emission spectra of sulforhodamine B released (trial 1) over time after the addition of **a) 1**-*E* (10 mol %), **b) 1**-*Z* (10 mol %), **c)** rotaxane **2** (10 mol %), and **d)** axle **3** (10 mol %) at 25 ºC. The LUVs are suspended in a buffer solution containing HEPES buffer (10 mM, pH 7.2) and NaCl (100 mM).

Table S2. Maximum fluorescence emission of sulforhodamine B at different time intervals upon addition of **1**-*E* to EYPC LUVs.

Event	Trials (T)			Release %			Mean	Standard Deviation
	T1	T2	T3	T1	T2	T3		
LUVs only	279428	279931.4	279225.8	0	0	0	0	0
+**1**-*E*	520239.7	497159.6	685061.3	24.99927	23.13087	42.79013	30.30676	10.8512
After 5 min	877049.4	856463.7	872224.1	62.0406	61.39028	62.52404	61.98497	0.56892
After 10 min	848919.2	846282.9	864738.9	59.12033	60.3062	61.73482	60.38712	1.30912
After 15 min	858689.9	843591.9	866129.9	60.13466	60.01966	61.88148	60.6786	1.04331
After 20 min	845434.7	848259.5	863732.1	58.7586	60.51668	61.62867	60.30132	1.4471
After 25 min	845529.9	843296.5	869510.3	58.76848	59.98821	62.23791	60.33153	1.76001
After 30 min	848018.3	849134.4	872224.1	59.02681	60.60984	62.52404	60.72023	1.75122
After 35 min	852272.3	849399.3	875090.4	59.46842	60.63805	62.82625	60.97758	1.70447
After 40 min	842643.3	850468.8	879747.9	58.46881	60.75193	63.31733	60.84602	2.42563
After 45 min	848595.1	852612.4	887902.2	59.08669	60.98018	64.1771	61.41465	2.57287
After 50 min	852714.3	855844.9	886058.2	59.51432	61.32439	63.98267	61.60712	2.24755
After 55 min	840038.2	856443.1	887856	58.19838	61.38809	64.17223	61.2529	2.98922
After 60 min	842809.5	856859.6	888910.8	58.48607	61.43244	64.28343	61.40065	2.89881
After 65 min	908743.4	858654.6	892085.6	65.33084	61.62357	64.61818	63.85753	1.9672
After 70 min	910743.4	857941.4	894330.3	65.53846	61.54762	64.85486	63.98031	2.13432
+ Triton-X	1242703	1219058	1227658	100	100	100	100	0.00E+00

Table S3. Maximum fluorescence emission of sulforhodamine B at different time intervals upon addition of **1**-*Z* to EYPC LUVs.

Event	Trials (T)			Release %			Mean	Standard Deviation
	T1	T2	T3	T1	T2	T3		
LUVs only	298393.3	303071.7	301182.7	0	0	0	0	0
+**1**-*Z*	401429.6	502180.5	496750.8	10.54177	20.8864	19.89485	17.10767	5.70781
After 5 min	780864.6	911515.6	904726.1	49.36225	63.82541	61.39755	58.19507	7.74517
After 10 min	916115.1	966350.6	968160.6	63.1999	69.57757	67.85066	66.87604	3.29865
After 15 min	944109.5	938289.1	947485.8	66.06404	66.63395	65.74744	66.14848	0.44924
After 20 min	932475.1	930738.5	934853.6	64.87371	65.84189	64.46239	65.05933	0.70824
After 25 min	926036.9	931014.8	931078.2	64.21501	65.87087	64.07832	64.7214	0.99781
After 30 min	925141.4	931704.9	929655	64.12339	65.94326	63.93354	64.66673	1.10958
After 35 min	928010.8	928690.7	927703.6	64.41696	65.62708	63.73502	64.59302	0.95824
After 40 min	930643.6	933381.2	931432.9	64.68632	66.11911	64.1144	64.97328	1.0327
After 45 min	922118.4	932953.6	928945.6	63.81411	66.07425	63.86137	64.58324	1.29147
After 50 min	926867.6	935611.9	930253.4	64.3	66.35311	63.99441	64.88251	1.28271
After 55 min	930479.6	933034.1	932146.9	64.66955	66.0827	64.18703	64.97976	0.98517
After 60 min	930774.4	936095.4	932673.6	64.69971	66.40382	64.24061	65.11471	1.13976
After 65 min	935035.3	937167.9	928609.2	65.13565	66.51633	63.82715	65.15971	1.34475
After 70 min	929613.9	932435.6	930296.7	64.58098	66.01992	63.99881	64.86657	1.04038
+ Triton-X	1275803	1256366	1284192	100	100	100	100	7.11E-15

Table S4. Maximum fluorescence emission of sulforhodamine B at different time intervals upon addition of rotaxane **2** to EYPC LUVs.

Event	Trials (T)			Release %			Mean	Standard Deviation
	T1	T2	T3	T1	T2	T3		
LUVs only	62155.15	55184.85	59314.59	0	0	0	0	0
+**2**	72947.56	65606.12	72342.63	6.25611	5.4766	7.48613	6.40628	1.01315
After 5 min	124663.1	113081.8	128994.6	36.23443	30.4261	40.0393	35.56661	4.84127
After 10 min	151524.7	136957	160348.7	51.80546	42.973	58.05593	50.9448	7.57821
After 15 min	170334.3	158367	177173.7	62.70897	54.22442	67.72383	61.55241	6.82362
After 20 min	178269.6	170422.2	180788.6	67.30888	60.55969	69.801	65.88986	4.78128
After 25 min	182293.1	177937.4	180973.8	69.64121	64.50906	69.90747	68.01925	3.04282
After 30 min	177541.3	174888.7	179524.5	66.8867	62.90693	69.07465	66.28943	3.12694
After 35 min	171634.5	175086.2	176868.6	63.46269	63.0107	67.54852	64.67397	2.49967
After 40 min	165909.8	171193.3	175573.9	60.14422	60.96489	66.80454	62.63789	3.63169
After 45 min	161584.8	172069.7	168648.2	57.63712	61.42548	62.82493	60.62917	2.68401
After 50 min	160103.1	171575.2	167850.1	56.77818	61.16561	62.36634	60.10338	2.94162
After 55 min	164803.3	170346.2	162406.8	59.5028	60.51974	59.2385	59.75368	0.67646
After 60 min	164829.7	168548.6	162496	59.51809	59.57507	59.28976	59.46097	0.15099
After 65 min	165263.1	168548.6	156169.4	59.76932	59.57507	55.65439	58.33292	2.32171
After 70 min	165942.1	168959	165482.2	60.16291	59.79072	61.00568	60.31977	0.62248
+ Triton-X	234665	245472.1	233343.6	100	1.00E+02	100	100	8.20E-15

Table S5. Maximum fluorescence emission of sulforhodamine B at different time intervals upon addition of axle **3** to EYPC LUVs.

Event	Trials (T)			Release %			Mean	Standard Deviation
	T1	T2	T3	T1	T2	T3		
LUVs only	62998.2	65881.8	67322.73	0	0	0	0	0
+**3**	71888.73	66824.48	67874.5	5.29522	0.54422	0.32999	2.05648	2.80688
After 5 min	79423.64	74911.05	77010.95	9.78303	5.2127	5.79413	6.92995	2.48788
After 10 min	86965.44	83399.91	83738.17	14.27494	10.11342	9.81741	11.40192	2.4925
After 15 min	92985.08	92282.67	96647.25	17.86025	15.24155	17.5378	16.87987	1.42796
After 20 min	97872.2	99516.25	102917.1	20.77103	19.41758	21.28756	20.49205	0.9657
After 25 min	101785.3	106620.7	109623.6	23.10167	23.51908	25.29845	23.97307	1.16664
After 30 min	105792.3	109168	113901.3	25.48828	24.98967	27.85672	26.11155	1.53178
After 35 min	109620.2	116961.8	118575.4	27.76816	29.48911	30.65215	29.30314	1.45096
After 40 min	114732	118449.7	120639.5	30.81279	30.34811	31.88658	31.01583	0.78908
After 45 min	117690.1	125870.4	128333.2	32.5746	34.63217	36.48786	34.56488	1.95749
After 50 min	121630.1	129753.2	132885.9	34.92129	36.87376	39.21064	37.0019	2.14754
After 55 min	125668.8	139683.8	135581.6	37.32677	42.60682	40.82284	40.25214	2.68589
After 60 min	127998.5	134727	137636.7	38.71433	39.7452	42.05191	40.17048	1.70895
After 65 min	132219.7	143459.4	140752.9	41.22847	44.78652	43.9156	43.3102	1.85467
After 70 min	133546.7	151467.3	142984.9	42.01883	49.40957	45.25046	45.55962	3.70505
+ Triton-X	230895.5	239098.3	234530.3	100	100	100	100	0.00E+00

S2.2.2. Dye release studies in EYPC/Chol 8:2 LUVs

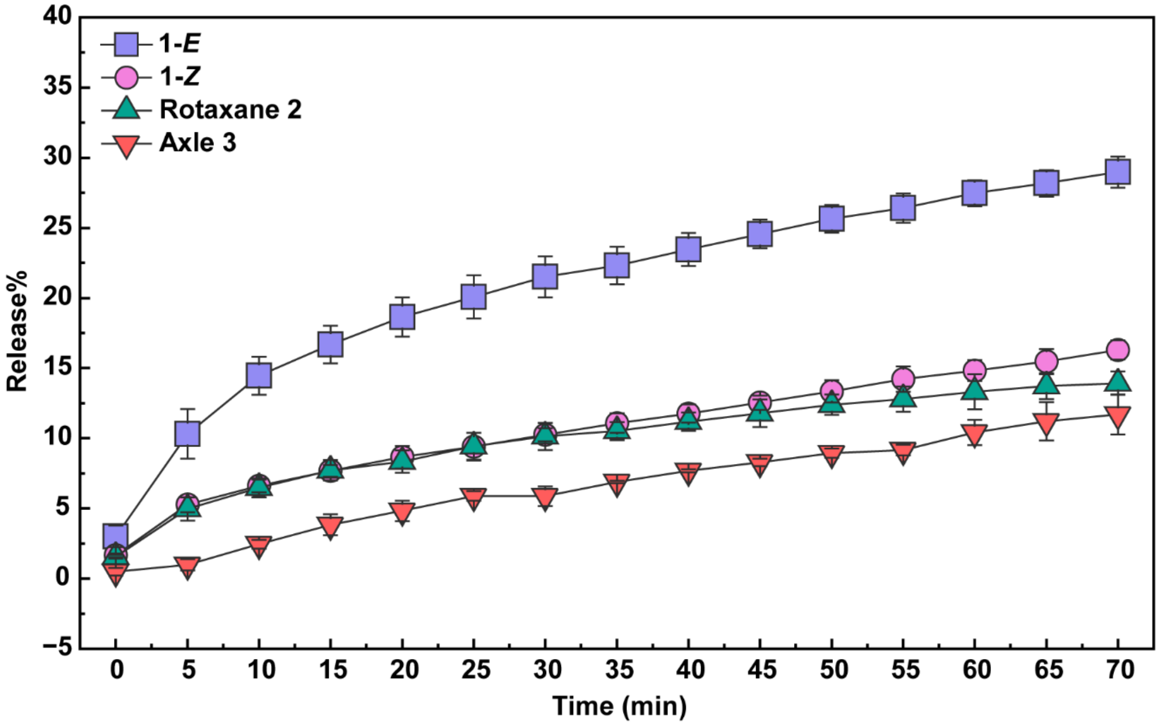

Figure S6. Percentage of sulforhodamine B released over time after adding **1-*E*, 1-*Z*,** rotaxane **2** or axle **3** to EYPC/Chol 8:2 LUVs at 25 °C. The LUVs are suspended in a buffer solution containing HEPES buffer (10 mM, pH 7.2) and NaCl (100 mM).

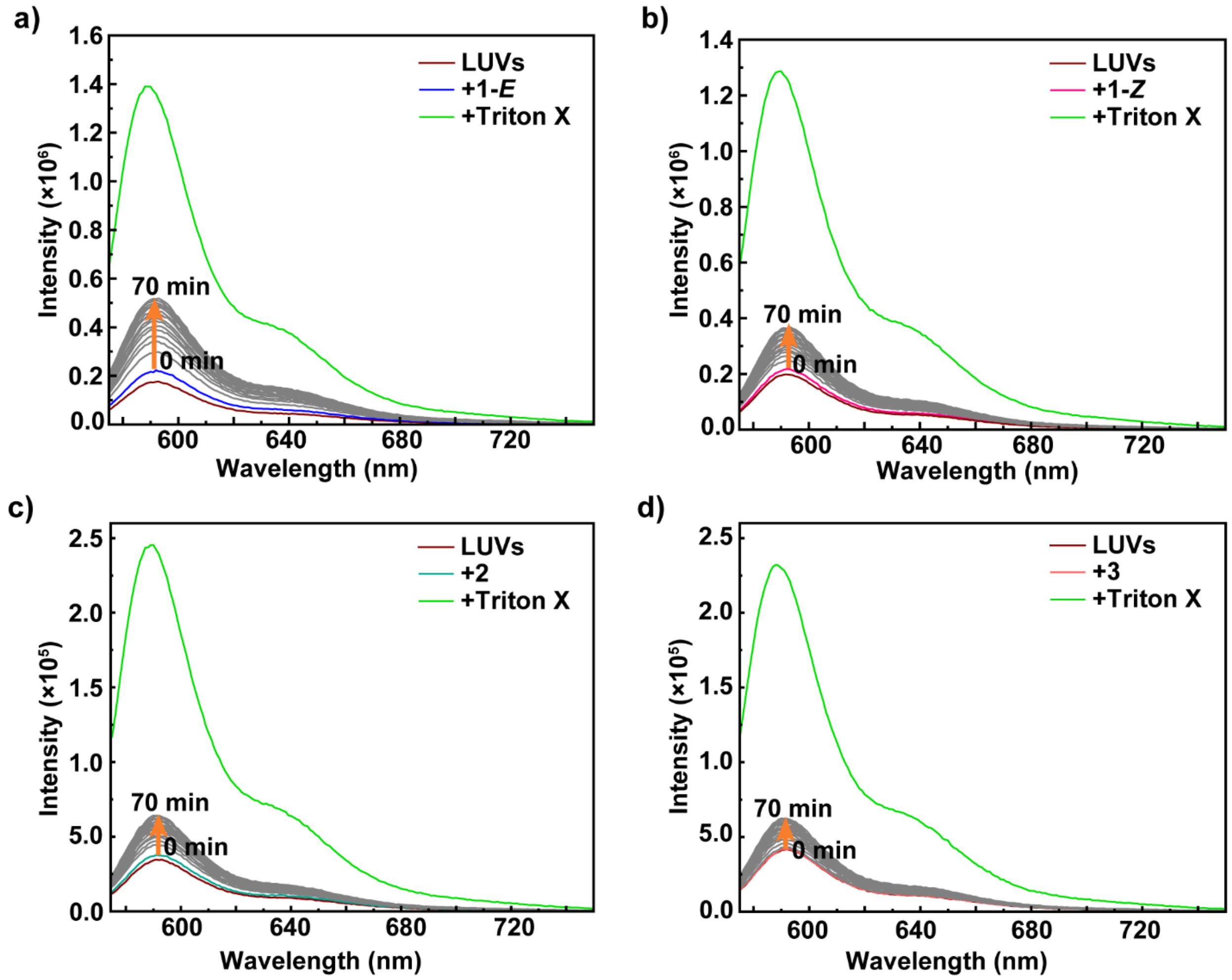

Figure S7. Emission spectra of sulforhodamine B released (trial 1) over time after the addition of **a) 1-***E* (10 mol %), **b) 1-***Z* (10 mol %), **c)** rotaxane **2** (10 mol %), and **d)** axle **3** (10 mol %) to EYPC/Chol 8:2 LUVs at 25 °C. The LUVs are suspended in a buffer solution containing HEPES buffer (10 mM, pH 7.2) and NaCl (100 mM).

Table S6. Maximum fluorescence emission of sulforhodamine B at different time intervals upon addition of **1**-*E* to EYPC/Chol 8:2 LUVs.

Event	Trials (T)			Release %			Mean	Standard Deviation
	T1	T2	T3	T1	T2	T3		
LUVs only	173762.3	196285.4	200328.6	0	0	0	0	0
+**1**-*E*	216800.4	219972.3	240166.1	3.53695	2.1296	3.34026	3.00227	0.76213
After 5 min	293363.4	294752.1	346648.9	9.82905	8.85275	12.26856	10.31678	1.75936
After 10 min	341693.7	346991.5	391286.1	13.80092	13.54939	16.01126	14.45386	1.3546
After 15 min	362129.4	379092.4	416373.3	15.48036	16.43546	18.11475	16.67686	1.33368
After 20 min	388210.9	397399.2	441638.4	17.6238	18.08135	20.23316	18.6461	1.39334
After 25 min	404168.4	412775	460774.1	18.93522	19.46373	21.83764	20.07886	1.5459
After 30 min	426761.4	424862.5	476970.3	20.79195	20.55047	23.19564	21.51269	1.46247
After 35 min	437426.2	434563	484854.8	21.6684	21.4226	23.85673	22.31591	1.34004
After 40 min	457630.5	444834.6	494983	23.32883	22.34608	24.70596	23.46029	1.18542
After 45 min	470551.2	459316	506374.8	24.39068	23.64804	25.66113	24.56662	1.01801
After 50 min	480385	474126	519647.8	25.19884	24.97956	26.77403	25.65081	0.9789
After 55 min	489748.7	482046.3	529650.1	25.96836	25.69164	27.6127	26.42424	1.0385
After 60 min	500626.9	496819.9	540401.8	26.86236	27.01987	28.5142	27.46547	0.91163
After 65 min	509698	504023.7	549261.8	27.60783	27.66753	29.25708	28.17748	0.93544
After 70 min	518317.7	511365.3	561252.4	28.31622	28.32759	30.26247	28.96876	1.1204
+ Triton-X	1390575	1308558	1392974	100	100	100	100	0.00E+00

Table S7. Maximum fluorescence emission of sulforhodamine B at different time intervals upon addition of **1**-*Z* to EYPC/Chol 8:2 LUVs.

Event	Trials (T)			Release %			Mean	Standard Deviation
	T1	T2	T3	T1	T2	T3		
LUVs only	198574.6	203143.9	204432.1	0	0	0	0	0
+**1**-*Z*	217182.3	220590.6	224143.1	1.71013	1.51536	1.75224	1.65925	0.12637
After 5 min	249185.3	267997.7	266539.8	4.65135	5.63296	5.52116	5.26849	0.53738
After 10 min	265832.3	284558.8	278521.1	6.18128	7.0714	6.58625	6.61298	0.44566
After 15 min	279009.3	296907.3	288565.6	7.39231	8.14394	7.47917	7.67181	0.41118
After 20 min	287307.5	313368.4	297133.2	8.15495	9.5737	8.2408	8.65648	0.79549
After 25 min	296751.1	324237.4	302103.5	9.02286	10.51774	8.68264	9.40774	0.97621
After 30 min	306004.7	327107.4	317962.9	9.8733	10.76701	10.09249	10.24427	0.46579
After 35 min	314647.3	340033.8	324594.3	10.6676	11.88975	10.682	11.07978	0.70149
After 40 min	325840.1	346393.5	329995.5	11.69627	12.44213	11.16215	11.76685	0.64291
After 45 min	334112	353831.9	340345.3	12.45649	13.0882	12.08221	12.5423	0.50846
After 50 min	339166.8	367289.1	348873.2	12.92105	14.25705	12.8403	13.33947	0.79567
After 55 min	350386.6	378494.7	356027.8	13.95221	15.23033	13.47633	14.21962	0.90706
After 60 min	355693.8	384084.6	365823.9	14.43995	15.71585	14.34716	14.83432	0.76483
After 65 min	360493.3	392863.2	373601.1	14.88106	16.47832	15.03853	15.46597	0.88025
After 70 min	369770.5	399569.3	385681.8	15.73367	17.06079	16.11245	16.30231	0.68363
+ Triton-X	1286661	1354471	1329336	100	100	100	100	8.20E-15

Table S8. Maximum fluorescence emission of sulforhodamine B at different time intervals upon addition of rotaxane **2** to EYPC/Chol 8:2 LUVs.

Event	Trials (T)			Release %			Mean	Standard Deviation
	T1	T2	T3	T1	T2	T3		
LUVs only	34676.76	34444.58	34895.94	0	0	0	0	0
+**2**	37775.43	38210.23	38099.4	1.47073	1.7312	1.48958	1.56384	0.14524
After 5 min	44510.69	47322.45	44196.99	4.66752	5.92038	4.32489	4.97093	0.83991
After 10 min	47441.04	50190.09	48063.78	6.05836	7.23873	6.12291	6.47333	0.66364
After 15 min	50422.62	53006.39	50281.95	7.47352	8.53348	7.15433	7.72045	0.72197
After 20 min	50884.1	54526.95	52388.38	7.69256	9.23253	8.1338	8.35296	0.79303
After 25 min	53563.22	57363.62	53790.14	8.96416	10.53664	8.7856	9.4288	0.96356
After 30 min	55044.24	58927.55	55336.55	9.6671	11.25563	9.50467	10.14247	0.96745
After 35 min	56651.68	58816.72	56227.95	10.43005	11.20468	9.91916	10.51796	0.64725
After 40 min	57907.89	60364.01	57720.45	11.02629	11.91602	10.61316	11.18515	0.6658
After 45 min	59094.58	62427.84	58380.21	11.58953	12.86483	10.91994	11.79143	0.98804
After 50 min	60682.13	63060.79	60041	12.34304	13.15582	11.69219	12.39701	0.73331
After 55 min	61752.95	64203.8	60438.3	12.85129	13.6813	11.87693	12.80317	0.90315
After 60 min	62439.85	66285.19	60990.23	13.17731	14.63818	12.13357	13.31635	1.25808
After 65 min	63753.07	66174.35	62330.99	13.80061	14.58723	12.75701	13.71495	0.91811
After 70 min	64059.05	66525.78	63030.11	13.94584	14.74879	13.08209	13.92557	0.83353
+ Triton-X	245365.4	251962.1	249954.6	100	100	100	100	0.00E+00

Table S9. Maximum fluorescence emission of sulforhodamine B at different time intervals upon addition of axle **3** to EYPC/Chol 8:2 LUVs.

Event	Trials (T)			Release %			Mean	Standard Deviation
	T1	T2	T3	T1	T2	T3		
LUVs only	42525.92	41663.16	41663.16	0	0	0	0	0
+**3**	44023.72	42297.95	42297.95	0.82185	0.33332	0.33332	0.49616	0.28205
After 5 min	45170.57	43083.2	43083.2	1.45112	0.74565	0.74565	0.98081	0.40731
After 10 min	47660.63	45996.57	45996.57	2.81742	2.27544	2.27544	2.4561	0.31291
After 15 min	51097.6	48156.66	48156.66	4.70329	3.40968	3.40968	3.84089	0.74686
After 20 min	52846.65	50073.17	50073.17	5.66299	4.41603	4.41603	4.83168	0.71994
After 25 min	53963.46	52516.27	52516.27	6.27578	5.69888	5.69888	5.89118	0.33308
After 30 min	51754.07	53622.9	53622.9	5.06349	6.27996	6.27996	5.87447	0.70233
After 35 min	55243.24	54688.6	54688.6	6.978	6.83955	6.83955	6.8857	0.07993
After 40 min	56293.14	56436.36	56436.36	7.55408	7.75729	7.75729	7.68955	0.11732
After 45 min	58204.23	57150.81	57150.81	8.6027	8.13244	8.13244	8.28919	0.2715
After 50 min	59532	58322.23	58322.23	9.33125	8.74754	8.74754	8.94211	0.337
After 55 min	58424.54	59551.63	59551.63	8.72358	9.39309	9.39309	9.16992	0.38654
After 60 min	63419.41	60522.54	60522.54	11.46427	9.9029	9.9029	10.42336	0.90146
After 65 min	65843.55	61494.26	61494.26	12.7944	10.41315	10.41315	11.2069	1.37482
After 70 min	66869.29	62388.96	62388.96	13.35722	10.88295	10.88295	11.70771	1.42852
+ Triton-X	224774.7	232106	232106	100	100	100	100	7.11E-15

S2.2.3. Dye release studies in EYPC/Chol 6:4 LUVs

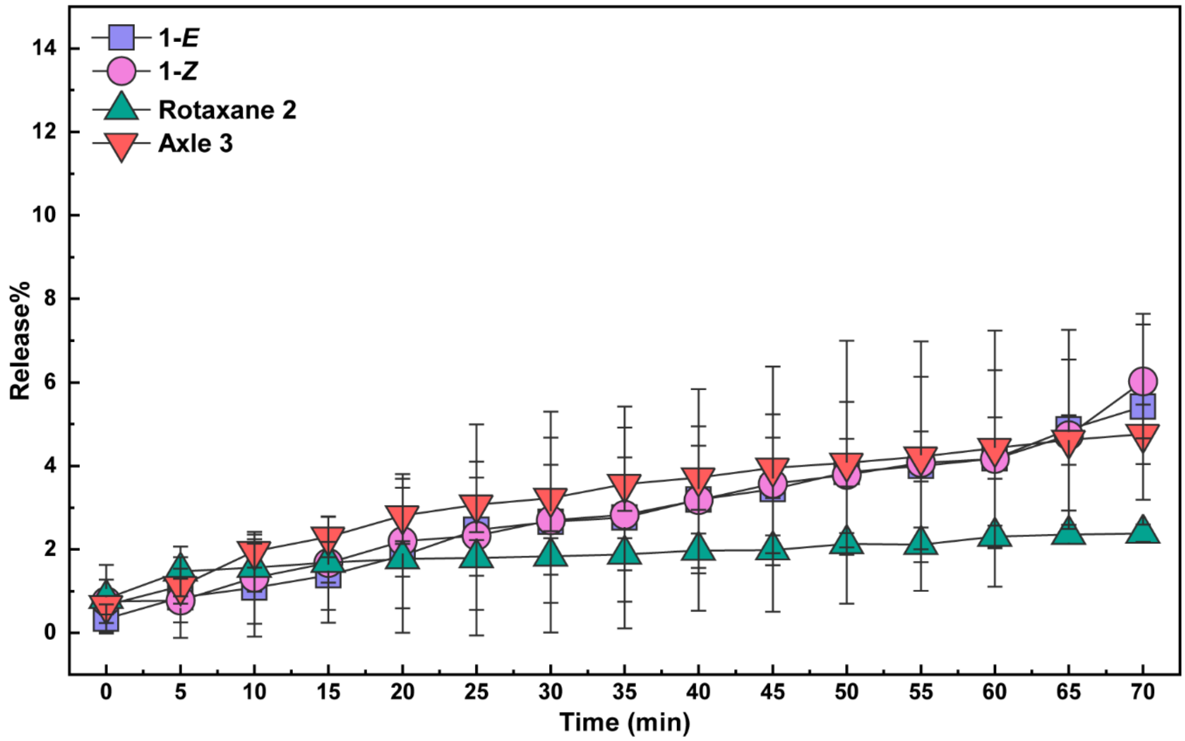

Figure S8. Percentage of sulforhodamine B released over time after adding **1-*E*, 1-*Z*,** rotaxane **2** or axle **3** to EYPC/Chol 6:4 LUVs at 25 °C. The LUVs are suspended in a buffer solution containing HEPES buffer (10 mM, pH 7.2) and NaCl (100 mM).

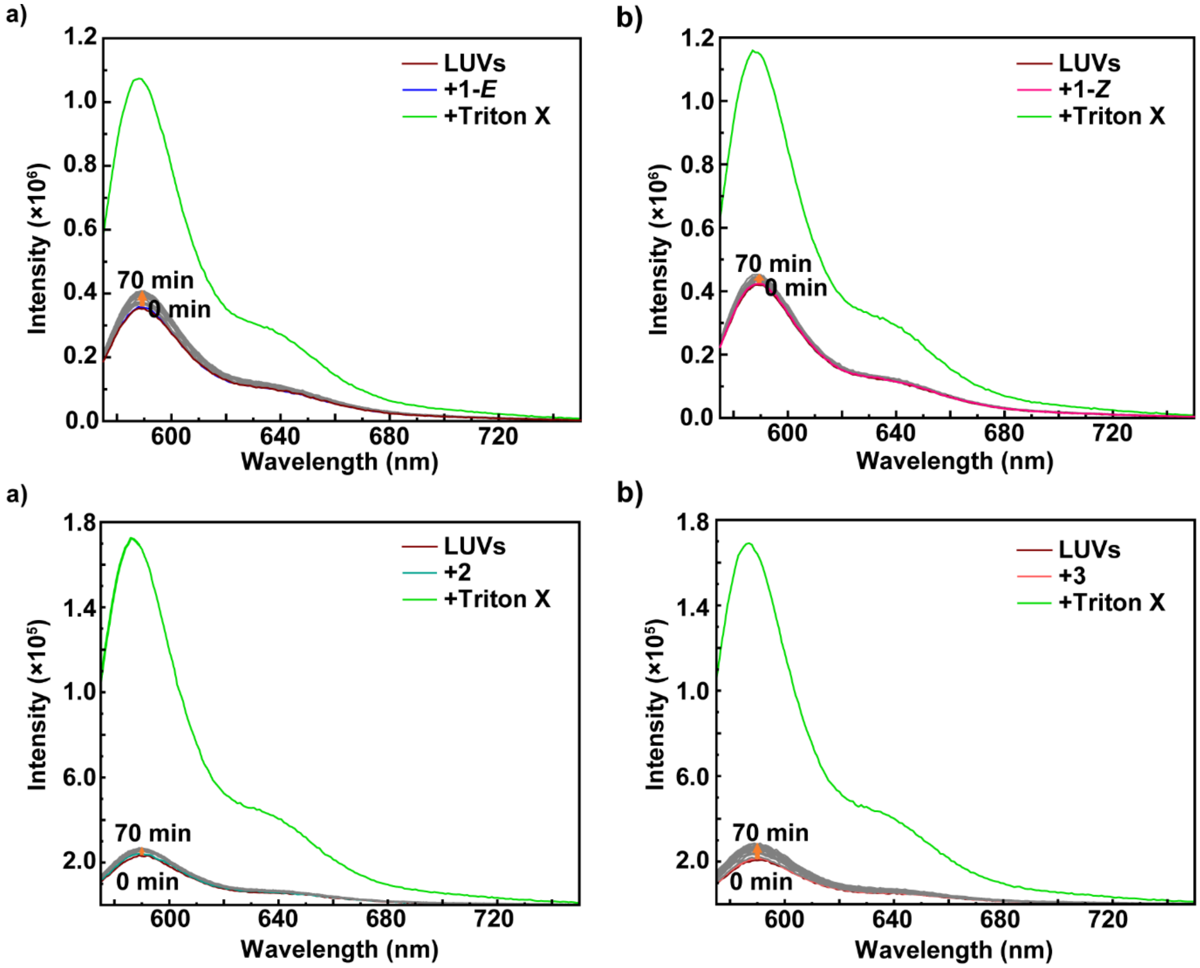

Figure S9. Emission spectra of sulforhodamine B released (trial 1) over time after the addition of **a) 1-***E* (10 mol %), **b) 1-***Z* (10 mol %), **c)** rotaxane **2** (10 mol %), and **d)** axle **3** (10 mol %) to EYPC/Chol 6:4 LUVs at 25 °C. The LUVs are suspended in a buffer solution containing HEPES buffer (10 mM, pH 7.2) and NaCl (100 mM).

Table S10. Maximum fluorescence emission of sulforhodamine B at different time intervals upon addition of **1**-*E* to EYPC/Chol 6:4 LUVs.

Event	Trials (T)			Release %			Mean	Standard Deviation
	T1	T2	T3	T1	T2	T3		
LUVs only	353454.2	389668.8	389742.3	0	0	0	0	0
+**1**-*E*	358770.3	390590.8	390665.3	0.73822	0.13594	0.12856	0.33424	0.34987
After 5 min	367523.7	390674.4	392929.3	1.95375	0.14828	0.44393	0.84865	0.96839
After 10 min	370444.3	390135.3	395499.7	2.35932	0.06879	0.80197	1.0767	1.16972
After 15 min	372412.3	392349	397785.7	2.63261	0.39519	1.12039	1.38273	1.14155
After 20 min	381852.5	392961.2	397744.2	3.94352	0.48545	1.11461	1.84786	1.84196
After 25 min	391841.3	393470.8	400562.3	5.3306	0.56059	1.50716	2.46611	2.52546
After 30 min	394047.3	393659.6	402289.5	5.63695	0.58843	1.74774	2.6577	2.64441
After 35 min	395025.6	394685.7	402539.9	5.77279	0.73972	1.78262	2.76504	2.65647
After 40 min	397972.5	397329.3	405900.4	6.18201	1.12951	2.25071	3.18741	2.6533
After 45 min	402121.3	397589.6	407042.6	6.75812	1.1679	2.40981	3.44528	2.93544
After 50 min	406215	397710.3	411554.1	7.32661	1.18568	3.03823	3.85017	3.14995
After 55 min	406383	400608.9	411472	7.34993	1.61307	3.02679	3.9966	2.98886
After 60 min	407952	400608.9	413759.9	7.56781	1.61307	3.34548	4.17546	3.0629
After 65 min	408401.1	413869.5	414372.8	7.63018	3.56829	3.43085	4.87644	2.3858
After 70 min	408206.4	411061.5	429173	7.60314	3.15425	5.49242	5.4166	2.22541
+ Triton-X	1073581	1067887	1107654	100	100	100	100	0.00E+00

Table S11. Maximum fluorescence emission of sulforhodamine B at different time intervals upon addition of **1**-*Z* to EYPC/Chol 6:4 LUVs.

Event	Trials (T)			Release %			Mean	Standard Deviation
	T1	T2	T3	T1	T2	T3		
LUVs only	398862.1	389293.2	419335.5	0	0	0	0	0
+**1**-*Z*	401580	399129.7	423101.2	0.40384	1.35329	0.50896	0.75536	0.52048
After 5 min	401190.5	399141.7	423911.5	0.34597	1.35494	0.61848	0.77313	0.52196
After 10 min	402573.9	408036.2	425463.8	0.55153	2.57862	0.8283	1.31948	1.09919
After 15 min	402457.5	409414.7	432050.1	0.53422	2.76828	1.71848	1.67366	1.1177
After 20 min	402712.2	416822.4	435915.4	0.57207	3.78741	2.2409	2.20013	1.60806
After 25 min	403342.8	419812.2	434930	0.66577	4.19874	2.10772	2.32407	1.7764
After 30 min	403857.3	423439.4	439040.1	0.74221	4.69777	2.66323	2.70107	1.97805
After 35 min	403941.3	425095.7	440149.4	0.7547	4.92564	2.81316	2.83117	2.08553
After 40 min	408920.6	425691.3	441935.7	1.49456	5.00758	3.0546	3.18558	1.76017
After 45 min	413423.9	428558.8	442598.9	2.16369	5.40209	3.14423	3.57	1.66066
After 50 min	413740.5	430408.1	445175.1	2.21073	5.6565	3.49243	3.78655	1.74161
After 55 min	414522.3	435488.8	445422.2	2.3269	6.3555	3.52582	4.06941	2.06858
After 60 min	415248.1	436875.3	445313	2.43475	6.54626	3.51107	4.16402	2.13211
After 65 min	422361.3	438766.9	448265.1	3.49168	6.8065	3.91006	4.73608	1.8052
After 70 min	441714.6	441564.3	452791.2	6.36731	7.19136	4.52181	6.02682	1.36696
+ Triton-X	1071870	1116154	1159211	100	100	100	100	0.00E+00

Table S12. Maximum fluorescence emission of sulforhodamine B at different time intervals upon addition of rotaxane **2** to EYPC/Chol 6:4 LUVs.

Event	Trials (T)			Release %			Mean	Standard Deviation
	T1	T2	T3	T1	T2	T3		
LUVs only	23641.99	26034.9	23277.92	0	0	0	0	0
+**2**	26431.18	26243.74	24165.6	1.71486	0.13974	0.59524	0.81661	0.81056
After 5 min	27083.57	27452.94	25272.12	2.11597	0.94891	1.33722	1.46736	0.59432
After 10 min	27096.21	27485.17	25666.08	2.12374	0.97048	1.60139	1.5652	0.57748
After 15 min	27205.37	27850.39	25757.02	2.19085	1.21487	1.66237	1.68937	0.48855
After 20 min	27205.57	28046.06	25930.49	2.19098	1.34581	1.77869	1.77182	0.42263
After 25 min	27223.9	28045.75	26030.45	2.20224	1.3456	1.84572	1.79785	0.43032
After 30 min	27306.42	28096.41	26056.38	2.25298	1.3795	1.86311	1.83186	0.43758
After 35 min	27328.31	28269.74	26093.59	2.26644	1.49549	1.88806	1.88333	0.3855
After 40 min	27498.41	28337.1	26255.52	2.37102	1.54056	1.99664	1.96941	0.4159
After 45 min	27349.61	28407.84	26363.4	2.27954	1.5879	2.06898	1.9788	0.35453
After 50 min	27537.14	28837.29	26451.51	2.39484	1.87527	2.12806	2.13272	0.25981
After 55 min	27698.12	28535.33	26513.2	2.49381	1.67321	2.16943	2.11215	0.41328
After 60 min	27861.36	29144.11	26649.61	2.59417	2.08059	2.2609	2.31189	0.26056
After 65 min	27842.71	29218.13	26797.19	2.58271	2.13012	2.35986	2.35756	0.2263
After 70 min	27869.84	29297.63	26807.61	2.59939	2.18332	2.36685	2.38319	0.20851
+ Triton-X	186289.9	175473.6	172408.5	100	100	100	100	0.00E+00

Table S13. Maximum fluorescence emission of sulforhodamine B at different time intervals upon addition of axle **3** to EYPC/Chol 6:4 LUVs.

Event	Trials (T)			Release %			Mean	Standard Deviation
	T1	T2	T3	T1	T2	T3		
LUVs only	20660.03	22562.58	22865.78	0	0	0	0	0
+**3**	21279.33	23870.75	23977.52	0.4172	0.84794	0.72466	0.66327	0.22184
After 5 min	22094.89	23783.14	25307.38	0.96662	0.79115	1.5915	1.11642	0.42067
After 10 min	23691.13	24923.05	26396.99	2.04196	1.53004	2.30173	1.95791	0.39265
After 15 min	24229.88	25297.29	27038.64	2.4049	1.77262	2.71998	2.29916	0.48245
After 20 min	24838.61	25849.76	28193.95	2.81498	2.13072	3.47304	2.80624	0.6712
After 25 min	25879.96	26135.98	28034.2	3.51651	2.31624	3.3689	3.06722	0.65454
After 30 min	26061.19	26131.1	28596.46	3.63859	2.31308	3.7354	3.22902	0.7947
After 35 min	26390.28	26922.44	28997.42	3.86029	2.82602	3.99676	3.56102	0.64018
After 40 min	26898.87	26937.84	29174.12	4.20291	2.836	4.11193	3.71695	0.76427
After 45 min	26840.44	27412.31	29849.45	4.16354	3.14355	4.55213	3.95307	0.72749
After 50 min	27385.19	27859.66	29415.18	4.53053	3.43352	4.26906	4.0777	0.57299
After 55 min	27627.51	28037.33	29667.54	4.69377	3.54868	4.43356	4.22534	0.60027
After 60 min	28029.45	28101.98	30103.75	4.96454	3.59059	4.71789	4.42434	0.73251
After 65 min	28475.2	28863.2	29799.89	5.26484	4.084	4.51983	4.62289	0.59713
After 70 min	28875.29	28960.75	29909.8	5.53436	4.14724	4.59147	4.75769	0.70835
+ Triton-X	169101	176838.1	176281.3	100	100	100	100	0.00E+00

S2.2.4. Dye release studies in DPPC LUVs

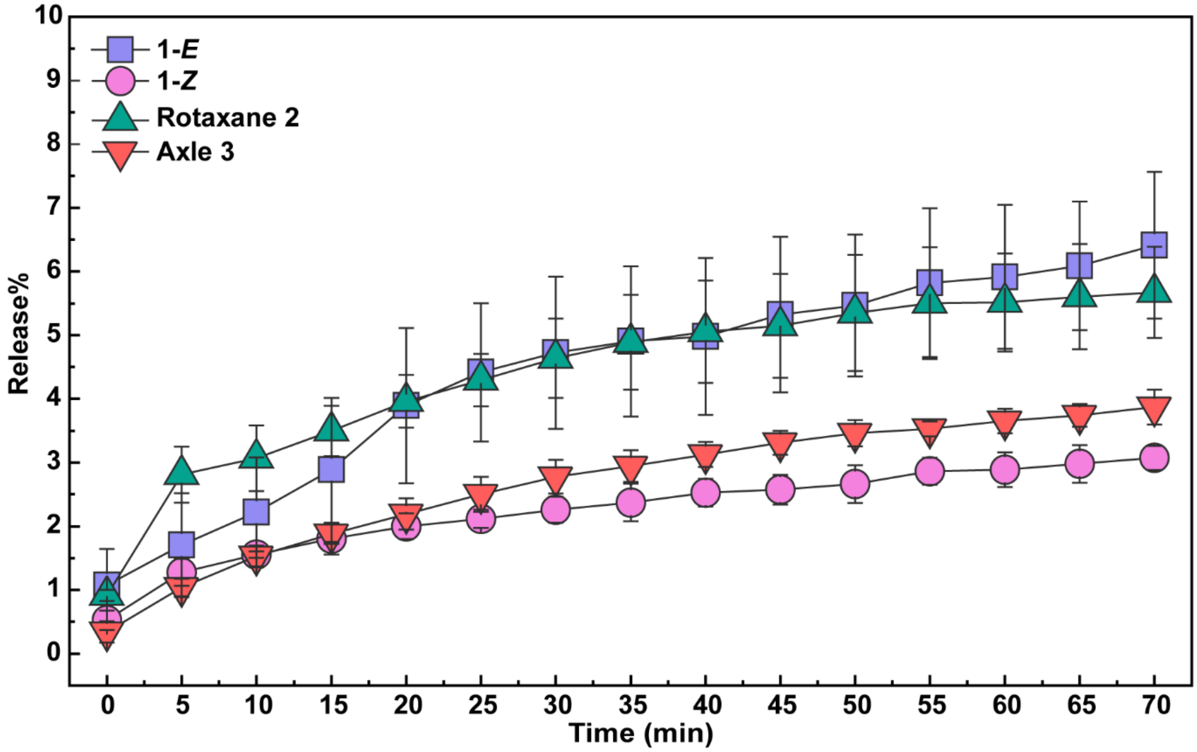

Figure S10. Percentage of sulforhodamine B released over time after adding **1-*E*, 1-*Z*,** rotaxane **2** or axle **3** to DPPC LUVs at 25 °C. The LUVs are suspended in a buffer solution containing HEPES buffer (10 mM, pH 7.2) and NaCl (100 mM).

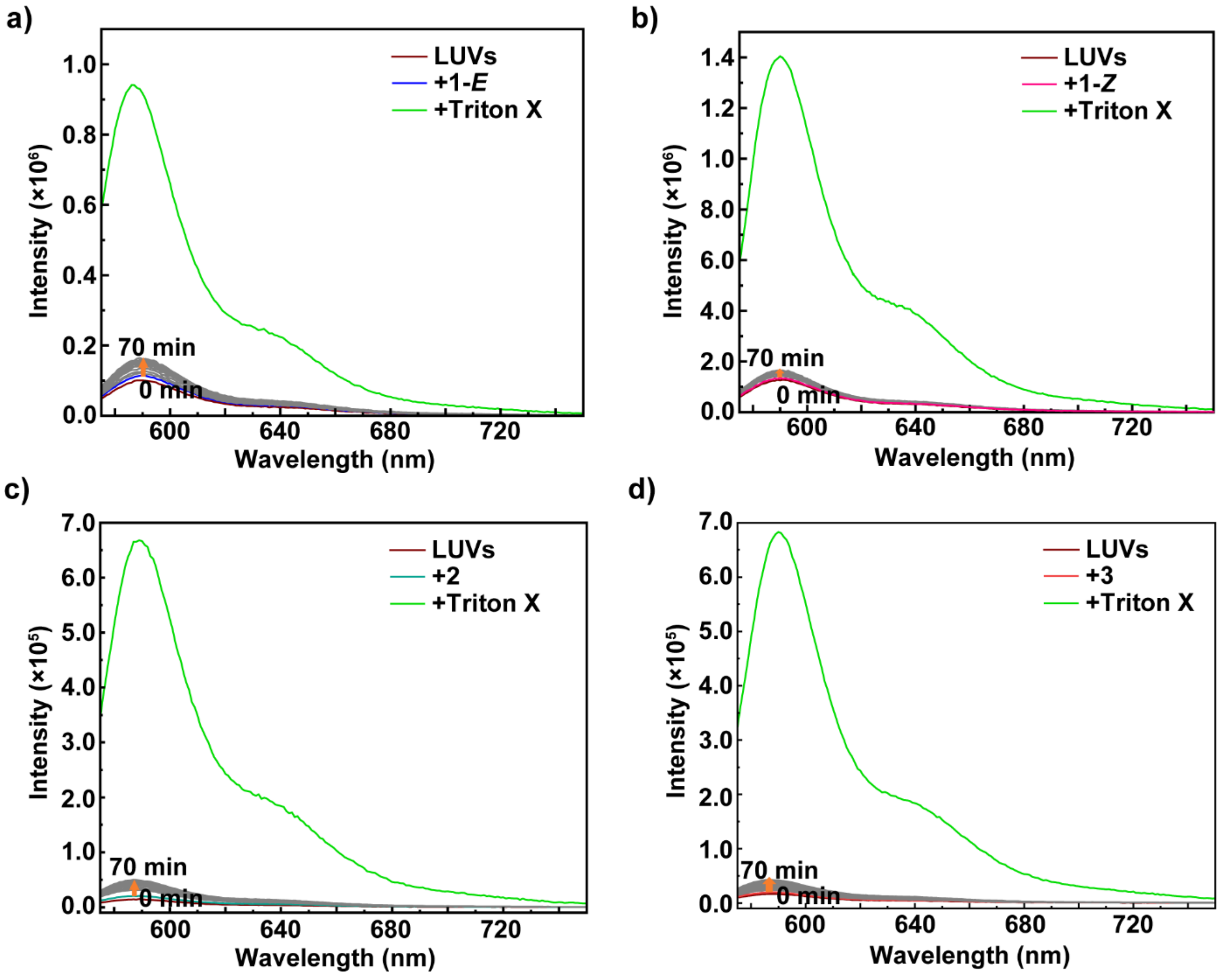

Figure S11. Emission spectra of sulforhodamine B released (trial 1) over time after the addition of **a) 1-*E*** (10 mol %), **b) 1-*Z*** (10 mol %), **c)** rotaxane **2** (10 mol %), and **d)** axle **3** (10 mol %) to DPPC LUVs at 25 °C. The LUVs are suspended in a buffer solution containing HEPES buffer (10 mM, pH 7.2) and NaCl (100 mM).

Table S14. Maximum fluorescence emission of sulforhodamine B at different time intervals upon addition of **1**-*E* to DPPC LUVs.

Event	Trials (T)			Release %			Mean	Standard Deviation
	T1	T2	T3	T1	T2	T3		
LUVs only	100642	124574.1	196748	0	0	0	0	0
+**1**-*E*	114736.7	133376.8	203603.1	1.67719	0.9989	0.58789	1.088	0.55009
After 5 min	121549.5	140122.5	207536.5	2.48787	1.76438	0.92522	1.72582	0.78203
After 10 min	126884.4	143386.1	214284.4	3.12269	2.13472	1.50393	2.25378	0.81592
After 15 min	135442.5	147180.2	221155.3	4.14105	2.56526	2.09318	2.93316	1.07236
After 20 min	144678.8	149908.9	241353.8	5.24011	2.87491	3.82541	3.98014	1.19017
After 25 min	145510.7	152952	255449.5	5.33911	3.22022	5.03427	4.5312	1.14552
After 30 min	149466.9	154947.1	258261.3	5.80987	3.44662	5.27541	4.84397	1.23929
After 35 min	151199.1	156838.6	259682.6	6.01599	3.66126	5.3973	5.02485	1.22075
After 40 min	152560.6	157357.9	259828	6.178	3.72018	5.40978	5.10266	1.25736
After 45 min	155739.2	160819.1	262967.7	6.55623	4.11296	5.67904	5.44941	1.23772
After 50 min	155280.8	162345.4	266983.6	6.50168	4.28615	6.02344	5.60376	1.16587
After 55 min	158726.4	164986.1	271474.6	6.91169	4.58581	6.40859	5.9687	1.22375
After 60 min	159233	166205.5	272423.6	6.97197	4.72419	6.48998	6.06205	1.18342
After 65 min	159616.6	168736.2	274745.5	7.01762	5.01136	6.68911	6.23937	1.07609
After 70 min	164221.1	170978.8	276918.7	7.56553	5.26584	6.87548	6.56895	1.18009
+ Triton-X	941021.4	1005815	1362785	100	100	100	100	0.00E+00

Table S15. Maximum fluorescence emission of sulforhodamine B at different time intervals upon addition of **1**-*Z* to DPPC LUVs.

Event	Trials (T)			Release %			Mean	Standard Deviation
	T1	T2	T3	T1	T2	T3		
LUVs only	127933.9	129291.1	130824.1	0	0	0	0	0
+**1**-*Z*	134231.4	134269.7	139513	0.4934	0.38878	0.69161	0.5246	0.15381
After 5 min	142174.7	144562.7	149829.8	1.11575	1.19256	1.5128	1.2737	0.21059
After 10 min	147822.9	148561.1	150935.5	1.55827	1.5048	1.60081	1.55463	0.04811
After 15 min	149784.3	149963.9	156999.6	1.71194	1.61434	2.08349	1.80326	0.24755
After 20 min	151837.4	153537	158887.1	1.8728	1.89337	2.23373	1.99997	0.20271
After 25 min	153890.9	155275	159519.8	2.03369	2.02909	2.28409	2.11562	0.14592
After 30 min	155437.2	156573.6	162180.5	2.15484	2.1305	2.49588	2.26041	0.20429
After 35 min	156519.8	157038	164934.5	2.23966	2.16676	2.71509	2.37384	0.29777
After 40 min	159780.8	159055.2	165489.5	2.49515	2.32428	2.75927	2.52623	0.21915
After 45 min	159514.8	160084.4	166543.8	2.47431	2.40466	2.84318	2.57405	0.23566
After 50 min	160795.5	160298.7	168412.8	2.57465	2.42139	2.99195	2.66266	0.29528
After 55 min	163066.2	164208.7	169864.9	2.75256	2.72672	3.10753	2.86227	0.2128
After 60 min	163860	163340.3	170959.2	2.81475	2.65891	3.19464	2.88943	0.27556
After 65 min	163920.7	165154.5	172555.8	2.8195	2.80058	3.32172	2.9806	0.29557
After 70 min	165625.9	167323	172182.6	2.9531	2.96992	3.29201	3.07168	0.191
+ Triton-X	1404288	1409861	1387152	100	100	100	100	7.11E-15

Table S16. Maximum fluorescence emission of sulforhodamine B at different time intervals upon addition of rotaxane **2** to DPPC LUVs.

Event	Trials (T)			Release %			Mean	Standard Deviation
	T1	T2	T3	T1	T2	T3		
LUVs only	14581.16	15147.35	16560.58	0	0	0	0	0
+**2**	20536.48	21560.34	22044.63	0.91163	1.00452	0.83117	0.91577	0.08675
After 5 min	31389.56	31377.85	38451.34	2.57299	2.54231	3.31781	2.81104	0.43915
After 10 min	32271.87	33291.46	40710.08	2.70805	2.84205	3.66015	3.07008	0.51539
After 15 min	35231.8	36834.06	42496.2	3.16115	3.39696	3.93086	3.49632	0.39436
After 20 min	38195.87	39706.99	45712.23	3.61488	3.84697	4.41829	3.96005	0.41347
After 25 min	41245.58	40910.74	48049.52	4.08172	4.03552	4.77253	4.29659	0.41282
After 30 min	42600	42373.99	51895.16	4.28905	4.26472	5.35538	4.63639	0.62279
After 35 min	44287.6	43073.17	54441.06	4.54739	4.37424	5.74125	4.88762	0.74431
After 40 min	44912.27	44172.08	56031.29	4.64301	4.54637	5.98226	5.05722	0.80257
After 45 min	45184.97	44935.51	56727.1	4.68475	4.66595	6.08772	5.14614	0.81549
After 50 min	46495.87	45565.79	58804.55	4.88542	4.76468	6.40259	5.3509	0.91279
After 55 min	47507.08	46790.95	59553.64	5.04022	4.95659	6.51612	5.50431	0.87725
After 60 min	48577.1	46730.25	58712	5.20401	4.94708	6.38856	5.51322	0.76888
After 65 min	48840.87	47220.62	59780.64	5.24439	5.02389	6.55052	5.60627	0.82515
After 70 min	49973.72	47820.16	59303.49	5.4178	5.1178	6.47821	5.67127	0.71475
+ Triton-X	667845	653562.7	676356.1	100	100	100	100	0.00E+00

Table S17. Maximum fluorescence emission of sulforhodamine B at different time intervals upon addition of axle **3** to DPPC LUVs.

Event	Trials (T)			Release %			Mean	Standard Deviation
	T1	T2	T3	T1	T2	T3		
LUVs only	16868.21	16817.71	17368.39	0	0	0	0	0
+**3**	19708.68	19837.59	18373.81	0.42643	0.44507	0.14848	0.34	0.16612
After 5 min	23589.55	24864.44	23569.43	1.00906	1.18593	0.91579	1.03693	0.13721
After 10 min	26361.01	28496.55	27175.61	1.42514	1.72123	1.44836	1.53158	0.16465
After 15 min	28636.88	30768.44	29646.98	1.76681	2.05606	1.81334	1.87874	0.15532
After 20 min	30324.77	33592.14	31463.23	2.02021	2.47221	2.08158	2.19133	0.24518
After 25 min	32039.04	35854.66	33715.03	2.27757	2.80566	2.41413	2.49912	0.27411
After 30 min	34217.24	37728.08	35291.72	2.60458	3.08177	2.64698	2.77778	0.26412
After 35 min	35727.85	38741.63	36135.69	2.83136	3.23115	2.77162	2.94471	0.24985
After 40 min	37227.68	39521.54	37530.56	3.05653	3.34609	2.97762	3.12675	0.19401
After 45 min	38740.82	40653.48	38655.59	3.28369	3.51291	3.14377	3.31346	0.18636
After 50 min	39718.71	41794.71	39523.2	3.4305	3.68111	3.2719	3.46117	0.20632
After 55 min	40239.38	41613.9	40531.96	3.50867	3.65446	3.42088	3.528	0.11799
After 60 min	40710.08	43118.79	41154.99	3.57933	3.87625	3.51289	3.65616	0.19348
After 65 min	41526.84	43534.95	41604.65	3.70195	3.93758	3.5793	3.73961	0.18209
After 70 min	42880.75	44821.67	41640.69	3.90521	4.12722	3.58462	3.87235	0.27279
+ Triton-X	682966.3	695336.4	694492.1	100	100	100	100	0.00E+00

S2.3. Release of sulforhodamine B from LUVs with light irradiation

To evaluate the effect of photoisomerization of rotaxane **1** on the permeability of large unilamellar vesicles (LUVs), the percentage of sulforhodamine B released after five cycles of light irradiation was assessed at wavelengths of 370 nm and 467 nm. Initially, 2946 µL of an extravesicular buffer (10 mM HEPES, 100 mM NaCl, pH 7.2) with 48 µL of LUVs encapsulating sulforhodamine B (12.5 mM) were combined in a quartz cuvette. Then, the mixture was placed in the fluorometer chamber and stirred at 150 rpm for 2 minutes to ensure homogeneity. The emission spectrum was recorded from 575 nm to 750 nm, using an excitation wavelength of 565 nm. All fluorescence studies were conducted at 25 °C, with the mixture continuously stirred at 150 rpm during the emission spectrum recordings. Next, 6 µL of a DMSO solution containing 10 mM rotaxane **1**-*E* was added to the cuvette, followed by 5 minutes of stirring in the fluorometer to allow time for incorporation into the bilayers. After this, the emission spectrum was recorded again. The cuvette was then irradiated at 370 nm for 1 minute outside the fluorometer, followed by another emission spectrum recording. The mixture was stirred for 5 minutes, and the emission spectrum was collected again. This process was repeated by irradiating the cuvette at 467 nm for 1 minute and recording the emission spectrum afterwards. The entire procedure was conducted for a total of 5 irradiation cycles. Finally, Triton X-100 was added to the cuvette, stirred for 8 minutes to lyse the vesicles, and the emission spectrum was recorded once more. The percentage of sulforhodamine release was calculated according to equation S1. This procedure was followed for all rotaxane **1**, control rotaxane **2**, and axle **3** separately in all lipid compositions, and each compound was tested three different times.

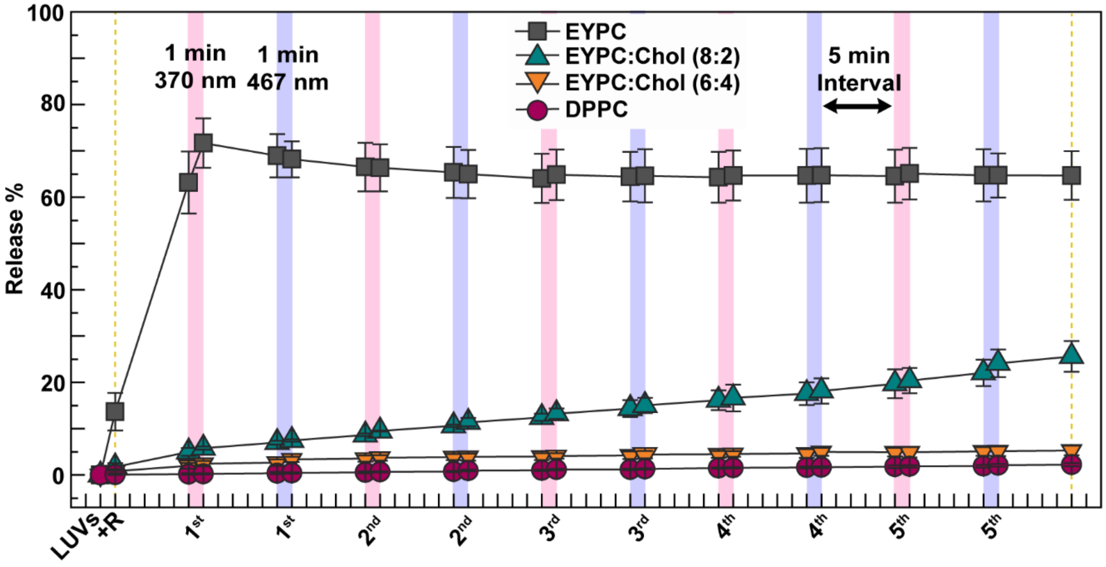

Figure S12: Percentage of sulforhodamine B released from LUVs containing rotaxane **2** upon five alternating light-irradiation cycles. The fluorescent emission of sulforhodamine B was measured before and after 1-minute irradiation with 370 nm light (pink strips) or 467 nm light (blue strips), and after 5 minutes post-irradiation. The sulforhodamine B (10 mM) was encapsulated in LUVs composed of different lipid/cholesterol ratios suspended in a solution of HEPES buffer (10 mM, pH 7.2). Rotaxane **2** (10 mol % with respect to the total lipid concentration) dissolved in DMSO was added to the LUVs

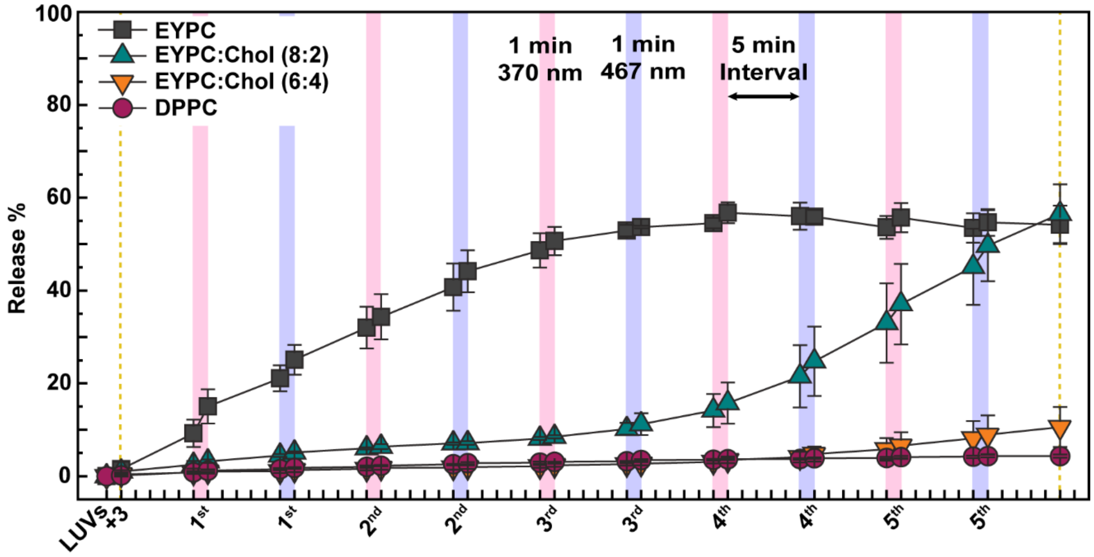

Figure S13: Percentage of sulforhodamine B released from LUVs containing axle **3** upon five alternating light-irradiation cycles. The fluorescent emission of sulforhodamine B was measured before and after 1-minute irradiation with 370 nm light (pink strips) or 467 nm light (blue strips), and after 5 minutes post-irradiation. The sulforhodamine B (10 mM) was encapsulated in LUVs composed of different lipid/cholesterol ratios suspended in a solution of HEPES buffer (10 mM, pH 7.2). axle **3** (10 mol % with respect to the total lipid concentration) dissolved in DMSO was added to the LUVs.

S2.3.1. Dye release studies in EYPC LUVs

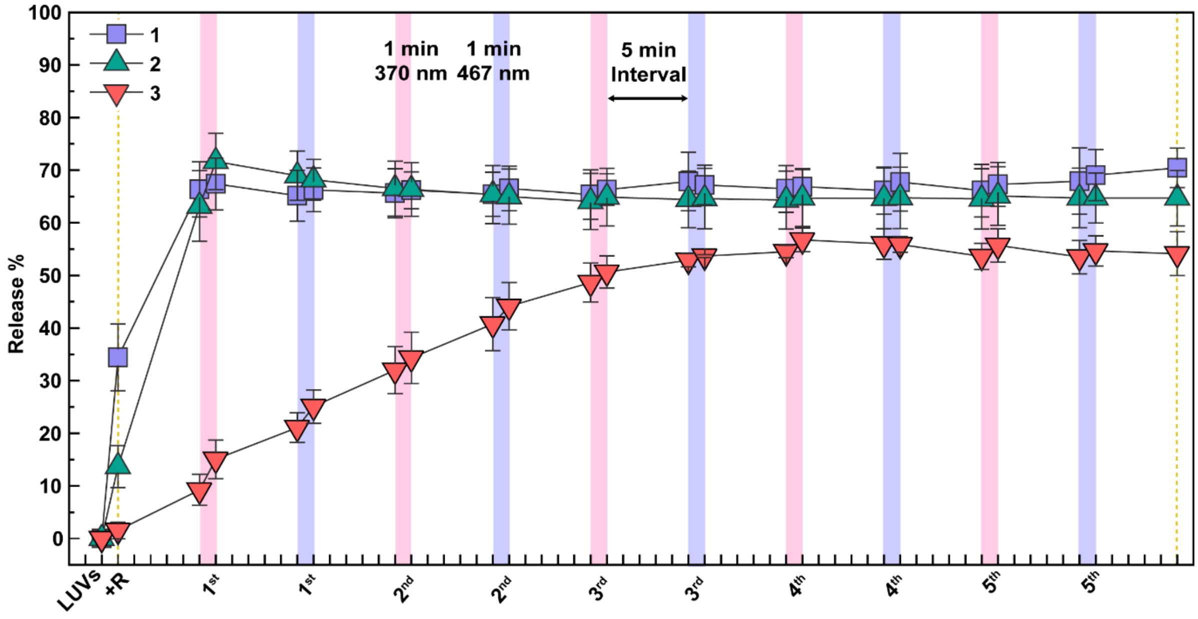

Figure S14. Percentage of sulforhodamine B released from EYPC LUVs upon five irradiation cycles after the addition of rotaxane **1,** rotaxane **2,** and axle **3** in 10 mol % at 25 °C. The fluorescent emission of sulforhodamine B was measured before and after 1-minute irradiation with 370 nm light (pink strips) or 467 nm light (blue strips), and after 5 minutes post-irradiation. The EYPC LUVs are suspended in a buffer solution containing HEPES buffer (10 mM, pH 7.2) and NaCl (100 mM).

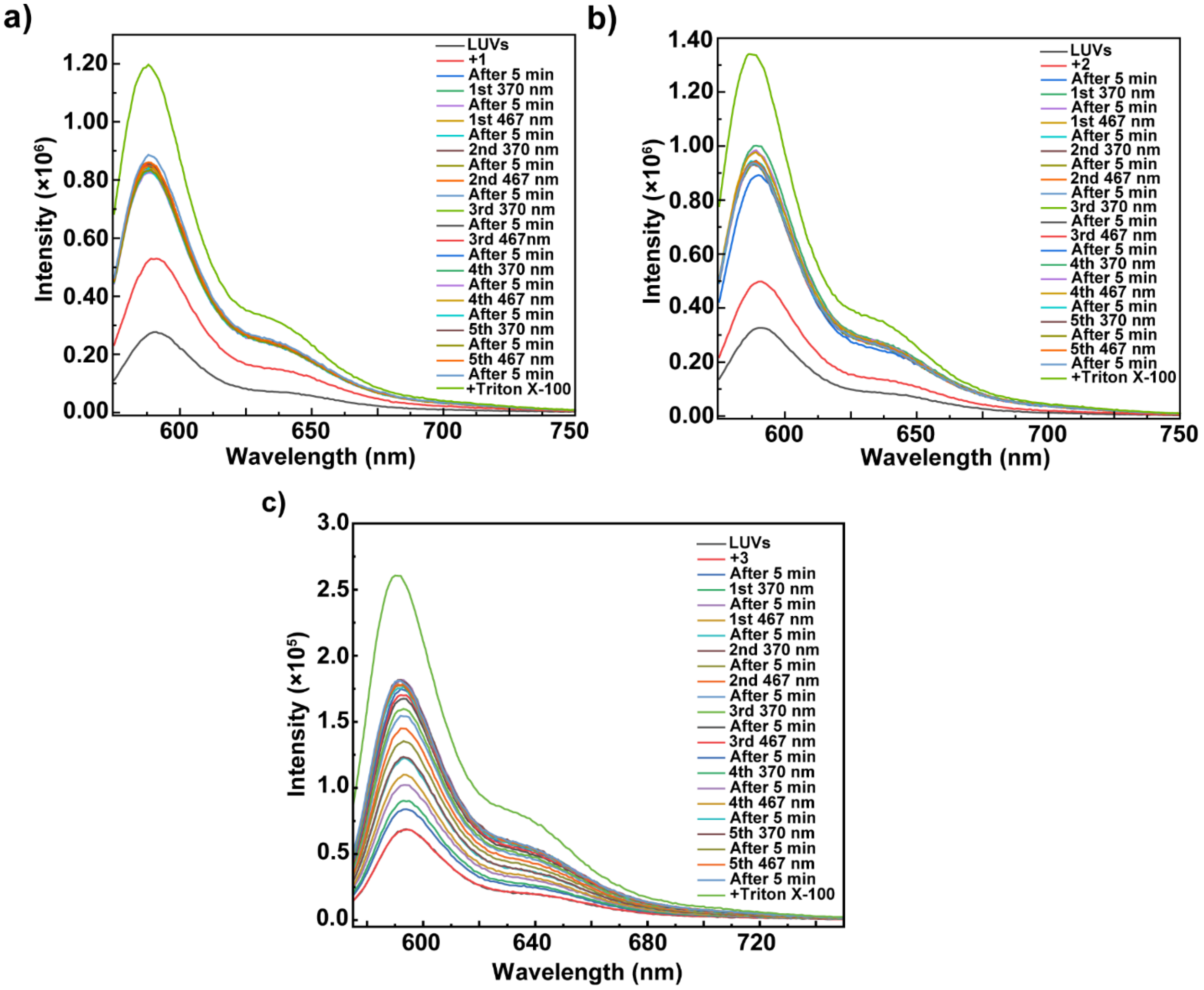

Figure S15. Emission spectra of sulforhodamine B (trial 1) released from EYPC LUVs during five light irradiation cycles after the addition of **a)** rotaxane **1** (10 mol %)**, b)** rotaxane **2** (10 mol %), and **c)** axle **3** (10 mol %) at 25 °C. The LUVs are suspended in a buffer solution containing HEPES buffer (10 mM, pH 7.2) and NaCl (100 mM).

Table S18. Maximum fluorescence emission of sulforhodamine B released from EYPC LUVs over five irradiation cycles after the addition of **1-*E***.

Event	Trials (T)			Release %			Mean	Standard Deviation
	T1	T2	T3	T1	T2	T3		
LUVs	277065.8	296874.9	313374.3	0	0	0	0	0
+**1**-*E*	529734.9	610940.4	672112.6	27.47594	35.93197	39.90031	34.43607	6.34583
After 5 min	831799.4	905370.8	935462.7	60.32325	69.61751	69.19117	66.37731	5.2473
After 1st irr. @370 nm	844335.3	915418.5	939965.4	61.68644	70.76707	69.69197	67.38183	4.96156
After 5 min	824861.6	893746.5	920823.5	59.56882	68.28759	67.56294	65.13978	4.83818
After 1st irr. @467 nm	842554.7	900560.3	927059.1	61.49281	69.06715	68.25648	66.27215	4.15883
After 5 min	831020.3	895987.6	925401.8	60.23853	68.544	68.07216	65.61823	4.66492
After 2nd irr. @370 nm	850001.6	900884.5	916903.9	62.3026	69.10424	67.12699	66.17794	3.49873
After 5 min	836099.2	898997	911936.5	60.79082	68.8883	66.57449	65.41787	4.1708
After 2nd irr. @467 nm	847534.3	913329.3	915898.4	62.0343	70.52804	67.01515	66.52583	4.26796
After 5 min	830106	901460.3	913859.8	60.1391	69.17012	66.78841	65.36588	4.68055
After 3rd irr. @370 nm	857937.9	901044.8	912904.4	63.16562	69.12258	66.68215	66.32345	2.99464
After 5 min	843522.3	905013.9	963680.5	61.59803	69.57669	72.32966	67.83479	5.57384
After 3rd irr. @ 467 nm	857332.3	912277.6	925304.4	63.09976	70.40772	68.06133	67.1896	3.73115
After 5 min	841981.6	907469.6	925338.8	61.43048	69.85763	68.06514	66.45109	4.43938
After 4th irr. @ 370 nm	858363.8	908824.1	918471.4	63.21194	70.0126	67.30132	66.84195	3.42353
After 5 min	839004	906657.3	919958.6	61.1067	69.76471	67.46674	66.11272	4.48501
After 4th irr. @ 467 nm	841942.3	921029	945951.7	61.42621	71.40896	70.3578	67.73099	5.48534
After 5 min	839742	918753.2	907210.5	61.18695	71.14859	66.04885	66.12813	4.98129
After 5th irr. @ 370 nm	858063.8	922525.4	917191.6	63.17931	71.58017	67.15899	67.30615	4.20236
After 5 min	834736.4	922729.4	956441.7	60.64263	71.6035	71.52453	67.92356	6.30559
After 5th irr. @ 467 nm	861265.4	931509.5	952107.8	63.52747	72.60802	71.0425	69.05933	4.85426
After 5 min	886591.6	921852.3	974876.9	66.2815	71.50315	73.57497	70.45321	3.75839
+Triton X	1196668	1170931	1212461	100	100	100	100	1.09E-14

Table S19. Maximum fluorescence emission of sulforhodamine B released from EYPC LUVs over five irradiation cycles after the addition of rotaxane **2**.

Event	Trials (T)			Release %			Mean	Standard Deviation
	T1	T2	T3	T1	T2	T3		
LUVs	316517	327253	340248	0	0	0	0	0
+**2**	451967.6	498671.9	426488.9	15.02347	16.84617	9.16242	13.67735	4.01485
After 5 min	930427.2	892274.8	960642.6	68.0917	55.52743	65.91209	63.17708	6.71383
After 1st irr. @370 nm	1009826	1001149	1016890	76.8982	66.22701	71.88798	71.67106	5.3389
After 5 min	982693.6	984724.1	983502.5	73.88881	64.61287	68.34078	68.94749	4.66764
After 1st irr. @467 nm	960350.6	977143.4	992083.3	71.41065	63.86788	69.25242	68.17699	3.88468
After 5 min	955755.3	945209.4	979061.6	70.90096	60.72957	67.86896	66.49983	5.22208
After 2nd irr. @370 nm	953573	945037.3	977219	70.65891	60.71266	67.6732	66.34826	5.10378
After 5 min	953691.1	934287.3	959388	70.67202	59.6562	65.7788	65.369	5.51933
After 2nd irr. @467 nm	953001.8	940209	943658.2	70.59556	60.23816	64.10763	64.98045	5.23358
After 5 min	947933.7	936541.2	926519.3	70.03343	59.8777	62.28676	64.06596	5.30649
After 3rd irr. @370 nm	952801.8	934425.2	945740.5	70.57338	59.66975	64.32886	64.85733	5.47099
After 5 min	950202.9	934713.9	936296.4	70.28512	59.69813	63.32549	64.43625	5.38019
After 3rd irr. @ 467 nm	955064	932631.4	938155.3	70.82429	59.49347	63.52299	64.61358	5.7436
After 5 min	950834.6	933640.7	933346.3	70.35518	59.59266	63.01207	64.31997	5.49918
After 4th irr. @ 370 nm	952011.9	935664.9	940812.4	70.48577	59.79159	63.80529	64.69422	5.40222
After 5 min	957473.1	936204.8	933367.8	71.09149	59.84464	63.01436	64.65016	5.79912
After 4th irr. @ 467 nm	958598.8	936785.9	934470.8	71.21634	59.90176	63.13154	64.74988	5.82831
After 5 min	956278	937905.9	929704.1	70.95894	60.01182	62.62511	64.53196	5.71725
After 5th irr. @ 370 nm	958273.6	941028.1	940489.6	71.18028	60.31865	63.77099	65.08998	5.54964
After 5 min	955574.9	935534.4	938176	70.88096	59.77877	63.52519	64.7283	5.64803
After 5th irr. @ 467 nm	945752.6	942096.5	941491.3	69.79152	60.42365	63.87741	64.69753	4.73748
After 5 min	950483.9	936937.9	940975.3	70.31629	59.91669	63.82259	64.68519	5.25319
+Triton X	1218110	1344807	1281494	100	100	100	100	0

Table S20. Maximum fluorescence emission of sulforhodamine B released from EYPC LUVs over five irradiation cycles after the addition of axle **3**.

Event	Trials (T)			Release %			Mean	Standard Deviation
	T1	T2	T3	T1	T2	T3		
LUVs	67018.39	67758.87	70628.19	0	0	0	0	0
+**3**	68501.51	75042.52	71645.4	0.76624	3.35583	0.50042	1.54083	1.57745
After 5 min	83943.78	94733.4	83995.86	8.74432	12.42809	6.57631	9.24958	2.95843
After 1st irr. @370 nm	90358.39	109276.8	98986.75	12.05836	19.12872	13.95117	15.04608	3.66014
After 5 min	102226.2	114010.6	119015.7	18.18976	21.30978	23.80453	21.10136	2.81318
After 1st irr. @467 nm	110017.1	120979.6	128595.5	22.21483	24.5206	28.5174	25.08428	3.18887
After 5 min	122555.2	133388.1	146064.7	28.69247	30.23763	37.11146	32.01386	4.48175
After 2nd irr. @370 nm	123557.9	143405.6	149781.6	29.21051	34.85306	38.94001	34.33453	4.88543
After 5 min	135453.9	157773.8	162938.6	35.35646	41.47298	45.41267	40.74737	5.06722
After 2nd irr. @467 nm	144970.2	161332.8	170443	40.27296	43.11274	49.10454	44.16341	4.50856
After 5 min	154640.6	172056.6	177586.2	45.26906	48.05357	52.61864	48.64709	3.71057
After 3rd irr. @370 nm	159855.2	176302.1	180399.1	47.96312	50.00962	54.00251	50.65842	3.07153
After 5 min	167607.5	181540	181042.5	51.96828	52.42287	54.31899	52.90338	1.24685
After 3rd irr. @ 467 nm	170317	184324.3	180224.2	53.36812	53.70568	53.91643	53.66341	0.27658
After 5 min	174725.9	186305.7	178980.4	55.64593	54.6186	53.30455	54.52303	1.17361
After 4th irr. @ 370 nm	181226	191040.5	181362.6	59.00414	56.8001	54.47651	56.76025	2.26408
After 5 min	181764.7	187328.9	179671.1	59.28244	55.09002	53.64437	56.00561	2.92843
After 4th irr. @ 467 nm	178380.4	186360.6	183382.4	57.53396	54.64389	55.47016	55.88267	1.48854
After 5 min	176281.4	181594.9	176240.4	56.44958	52.44816	51.9566	53.61811	2.46441
After 5th irr. @ 370 nm	181826.5	183799.3	180985.8	59.31435	53.4638	54.29112	55.68976	3.16613
After 5 min	177494.7	178704.5	176912.3	57.07639	51.11646	52.28711	53.49332	3.15775
After 5th irr. @ 467 nm	179066.9	181481.6	179821	57.88865	52.39599	53.7181	54.66758	2.86679
After 5 min	181054	179297.8	176546.1	58.91527	51.38981	52.10697	54.13735	4.15331
+Triton X	260577	284803.7	273898.3	100	100	100	100	0

S2.3.2. Dye release studies in EYPC/Chol 8:2 LUVs

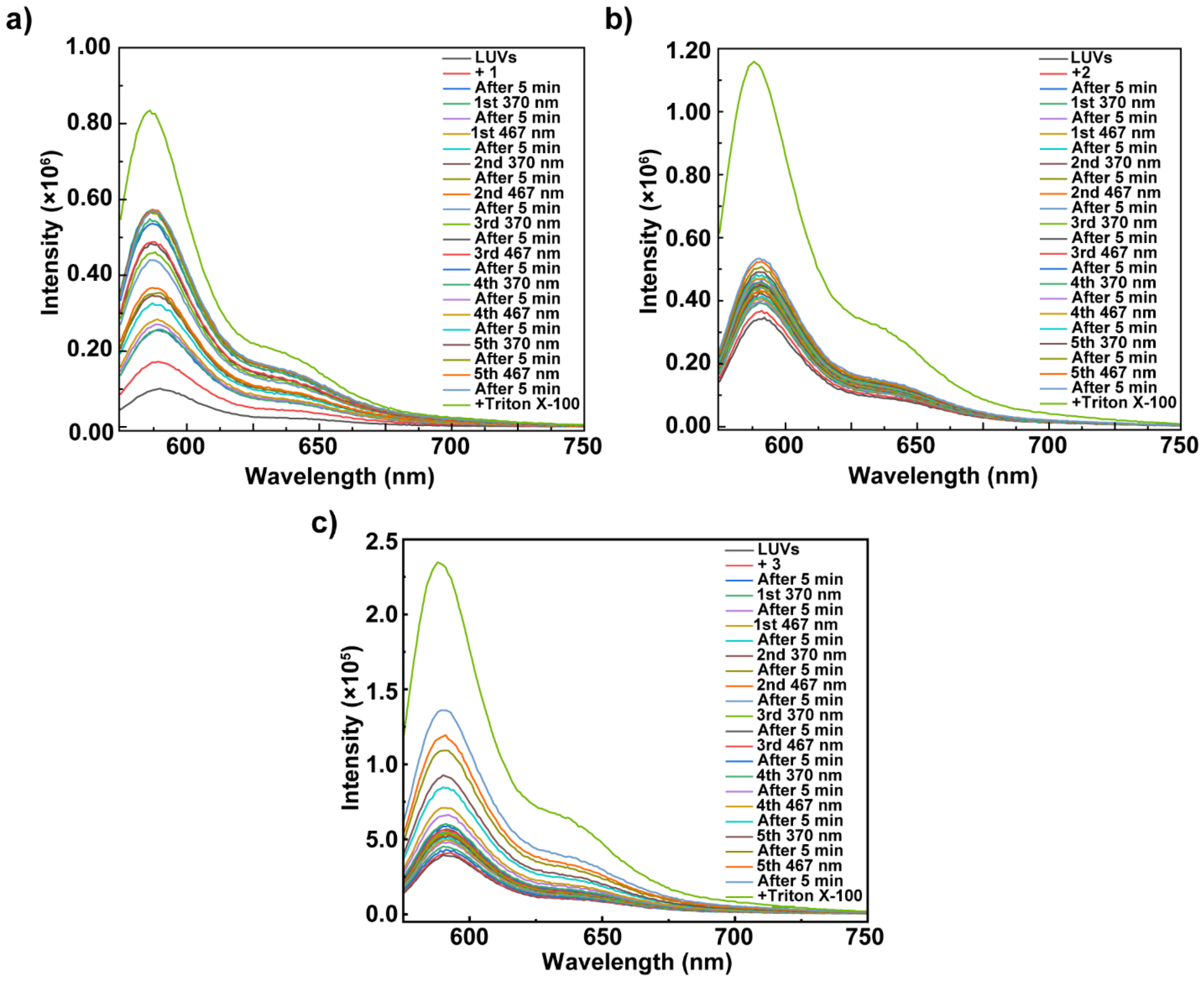

Figure S16. Emission spectra of sulforhodamine B (trial 1) released from EYPC/Chol 8:2 LUVs during five light irradiation cycles after the addition of **a)** rotaxane **1** (10 mol %), **b)** rotaxane **2** (10 mol %), and c) axle **3** (10 mol %) at 25 °C. The LUVs are suspended in a buffer solution containing HEPES buffer (10 mM, pH 7.2) and NaCl (100 mM)

Table S21. Maximum fluorescence emission of sulforhodamine B released from EYPC/Chol 8:2 over five irradiation cycles after the addition of **1-***E* to LUVs

Event	Trials (T)			Release %			Mean	Standard Deviation
	T1	T2	T3	T1	T2	T3		
LUVs	374785.3	403486.2	413003.2	0	0	0	0	0
+**1-***E*	419945.3	448279.6	458299.2	5.99693	5.44132	6.03734	5.8252	0.33306
After 5 min	511388.3	549310.8	561201.2	18.13996	17.71417	19.75276	18.53563	1.07535
After 1st irr. @370 nm	527637.6	556532.1	565199.8	20.29775	18.59138	20.28572	19.72495	0.98172
After 5 min	540183.8	575918.3	583184.9	21.9638	20.94634	22.68289	21.86434	0.87253
After 1st irr. @467 nm	548972.3	585143.6	588674.4	23.13086	22.067	23.41456	22.87081	0.71042
After 5 min	572510.8	611163.2	619757.9	26.25661	25.22775	27.55757	26.34731	1.16756
After 2nd irr. @370 nm	590612.1	622957.4	643643.8	28.66035	26.66047	30.74122	28.68735	2.04051
After 5 min	591865.8	631708.9	648952.6	28.82682	27.72357	31.44882	29.33307	1.91353
After 2nd irr. @467 nm	622392.4	641972.8	663585.1	32.88056	28.97037	33.39914	31.75002	2.42117
After 5 min	656210	704298.2	697821.8	37.3713	36.54141	37.96241	37.29171	0.71383
After 3rd irr. @370 nm	687948.1	709995.3	725274.8	41.5859	37.23347	41.62152	40.14696	2.52323
After 5 min	705323.4	720167.9	734288.2	43.89324	38.4692	42.82288	41.72844	2.87287
After 3rd irr. @ 467 nm	716403.3	725950.1	756239.1	45.36457	39.17159	45.74863	43.42827	3.69138
After 5 min	766784.6	764238.4	805119.1	52.05486	43.8227	52.26367	49.38041	4.81425
After 4th irr. @ 370 nm	779764.3	774325.3	827844.4	53.77849	45.04802	55.29264	51.37305	5.52971
After 5 min	804958.8	790943.8	833866.9	57.12415	47.06677	56.09536	53.42876	5.53361
After 4th irr. @ 467 nm	806122.8	807528.1	846208.3	57.27872	49.08136	57.74029	54.70012	4.87146
After 5 min	828965.7	837966.8	860110.5	60.3121	52.77893	59.59327	57.56143	4.15733
After 5th irr. @ 370 nm	828035.4	833655.4	858582.4	60.18856	52.2552	59.3896	57.27779	4.36799
After 5 min	834624.6	838118.2	859645.9	61.06357	52.79732	59.53135	57.79741	4.39746
After 5th irr. @ 467 nm	834965.6	848696.8	859938.7	61.10885	54.08236	59.57037	58.25386	3.69361
After 5 min	848327.7	853899.4	865296.7	62.88324	54.71435	60.28452	59.29404	4.17354
+Triton X	1127836	1226695	1163268	100	100	100	100	0.00E+00

Table S22. Maximum fluorescence emission of sulforhodamine B released from EYPC/Chol 8:2 LUVs over five irradiation cycles after the addition of rotaxane **2**.

**Event**	**Trials (T)**			**Release %**			**Mean**	**Standard Deviation**
	**T1**	**T2**	**T3**	**T1**	**T2**	**T3**		
LUVs	346352.3	325659.3	337062.3	0	0	0	0	0
+**2**	367988.9	337164.3	348665.4	2.68448	1.26069	1.30037	1.74851	0.81081
After 5 min	393779.3	364303.9	380725.3	5.88434	4.2346	4.89331	5.00408	0.83043
After 1st irr. @370 nm	392661.3	375943.3	392376.8	5.74563	5.51004	6.19909	5.81825	0.35022
After 5 min	402091.7	386717.3	403593.6	6.91566	6.69063	7.45615	7.02081	0.39345
After 1st irr. @467 nm	407129	391325.3	405708.8	7.54065	7.19556	7.69321	7.47647	0.25496
After 5 min	413959.4	404560.3	416879.6	8.3881	8.64584	8.94511	8.65968	0.27876
After 2nd irr. @370 nm	422917.8	411554.6	423235.9	9.49958	9.41225	9.65746	9.5231	0.12429
After 5 min	429838.6	423079.8	433742.2	10.35825	10.67517	10.83491	10.62277	0.24261
After 2nd irr. @467 nm	429936.2	434612.4	443319.4	10.37037	11.9389	11.90822	11.40583	0.89687
After 5 min	440063	441296.2	453847.5	11.62681	12.67129	13.0881	12.46207	0.75278
After 3rd irr. @370 nm	445827.4	457921.5	452154.5	12.34201	14.49306	12.89836	13.24448	1.11651
After 5 min	448782.7	474465.3	462724.6	12.70867	16.30591	14.08295	14.36584	1.81523
After 3rd irr. @ 467 nm	454911.8	478219.8	469855.2	13.46912	16.71732	14.88208	15.02284	1.62867
After 5 min	460956.8	493781.5	478146.2	14.21913	18.42254	15.81125	16.15098	2.1222
After 4th irr. @ 370 nm	458341.9	504799.8	483155.2	13.89469	19.62991	16.37261	16.6324	2.87642
After 5 min	468135.7	508278.6	494457.3	15.10982	20.01111	17.63923	17.58672	2.45107
After 4th irr. @ 467 nm	470656.4	515988.7	498979.9	15.42257	20.85597	18.14608	18.14154	2.7167
After 5 min	481324.5	535039.4	509778.7	16.74618	22.94352	19.3563	19.682	3.11148
After 5th irr. @ 370 nm	491186.9	538777.7	513674.9	17.96982	23.35315	19.79295	20.37197	2.73797
After 5 min	507735.9	556660.9	523142.5	20.02308	25.31276	20.85397	22.06327	2.84464
After 5th irr. @ 467 nm	524459.7	577165.8	539435.3	22.09802	27.55965	22.6799	24.11252	2.99944
After 5 min	534086.6	594079.1	552184.8	23.29244	29.41298	24.10874	25.60472	3.32321
+Triton X	1152341	1238249	1229363	100	100	100	100	8.20E-15

Table S23. Maximum fluorescence emission of sulforhodamine B released from EYPC/Chol 8:2 LUVs over five irradiation cycles after the addition of axle **3**.

Event	Trials (T)			Release %			Mean	Standard Deviation
	T1	T2	T3	T1	T2	T3		
LUVs	39458.67	39419.17	39563.37	0	0	0	0	0
+**3**	40972.52	41872.46	41731.79	0.77533	1.22161	1.12735	1.04143	0.23522
After 5 min	42769.68	44199.8	45895.25	1.69576	2.3805	3.29193	2.45606	0.80076
After 1st irr. @370 nm	45196.33	45643.18	46606.29	2.93859	3.09922	3.66159	3.23313	0.37965
After 5 min	47943.15	47902.19	48838.78	4.34539	4.22409	4.82226	4.46391	0.31621
After 1st irr. @467 nm	49511.68	49334.98	49877.72	5.14872	4.93754	5.3624	5.14956	0.21243
After 5 min	51285.53	51072.02	51441.3	6.05721	5.8025	6.1753	6.01167	0.19053
After 2nd irr. @370 nm	52664.81	50992.17	52253.68	6.76362	5.76274	6.59765	6.37467	0.53641
After 5 min	53827.93	53305.74	52961.3	7.35933	6.91477	6.96555	7.07988	0.24333
After 2nd irr. @467 nm	54270.84	53333.43	52961.3	7.58617	6.92856	6.96555	7.16009	0.36946
After 5 min	54807.67	55544.58	55909.47	7.86111	8.02959	8.49829	8.12966	0.33017
After 3rd irr. @370 nm	55331.44	57048.5	56180.59	8.12936	8.77846	8.63924	8.51569	0.34174
After 5 min	56536.96	61600.96	60326.33	8.74678	11.04535	10.7946	10.19557	1.26094
After 3rd irr. @ 467 nm	56197.64	65591.62	62748.64	8.57299	13.03248	12.05395	11.21981	2.34384
After 5 min	59029.39	72045.11	70692.95	10.0233	16.24597	16.18417	14.15115	3.57496
After 4th irr. @ 370 nm	60237.09	75920.42	74998.39	10.64183	18.17567	18.42256	15.74669	4.42266
After 5 min	66376.98	88877.76	89807.44	13.78642	24.62773	26.12174	21.51196	6.73209
After 4th irr. @ 467 nm	70950.41	96873.8	96374.59	16.12874	28.60934	29.53598	24.75802	7.48752
After 5 min	84649.92	114434.6	113687.3	23.14504	37.35366	38.53679	33.01183	8.56534
After 5th irr. @ 370 nm	92722.63	120211.6	123564.6	27.27955	40.23031	43.672	37.06062	8.64369
After 5 min	109373.8	136147.7	138366.1	35.80757	48.16565	51.36724	45.11349	8.21661
After 5th irr. @ 467 nm	119490.3	145215.6	145974.4	40.98883	52.68096	55.32276	49.66418	7.62831
After 5 min	136153.3	156446.9	158471.3	49.52288	58.27354	61.81985	56.53876	6.32938
+Triton X	234711	240243.9	231909.2	100	100	100	100	0.00E+00

S2.3.3. Dye release studies in EYPC/Chol 6:4 LUVs

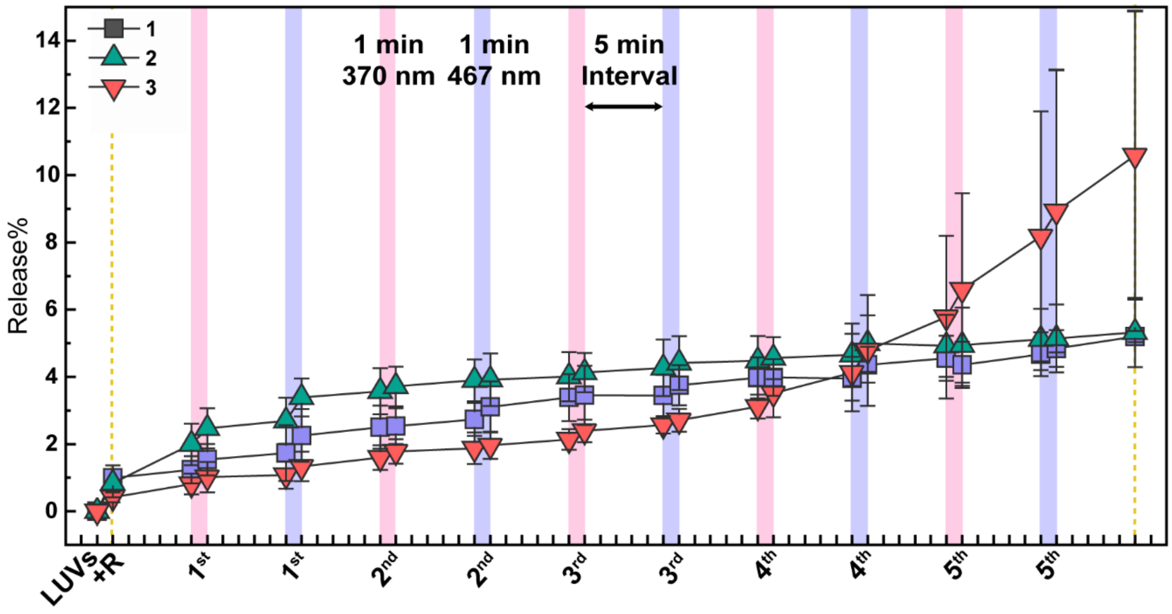

Figure S17. Percentage of sulforhodamine B released from EYPC/Chol 6:4 LUVs upon five irradiation cycles after the addition of rotaxane **1,** rotaxane **2**, and axle **3** in 10 mol % at 25 °C. The EYPC LUVs are suspended in a buffer solution containing HEPES buffer (10 mM, pH 7.2) and NaCl (100 mM).

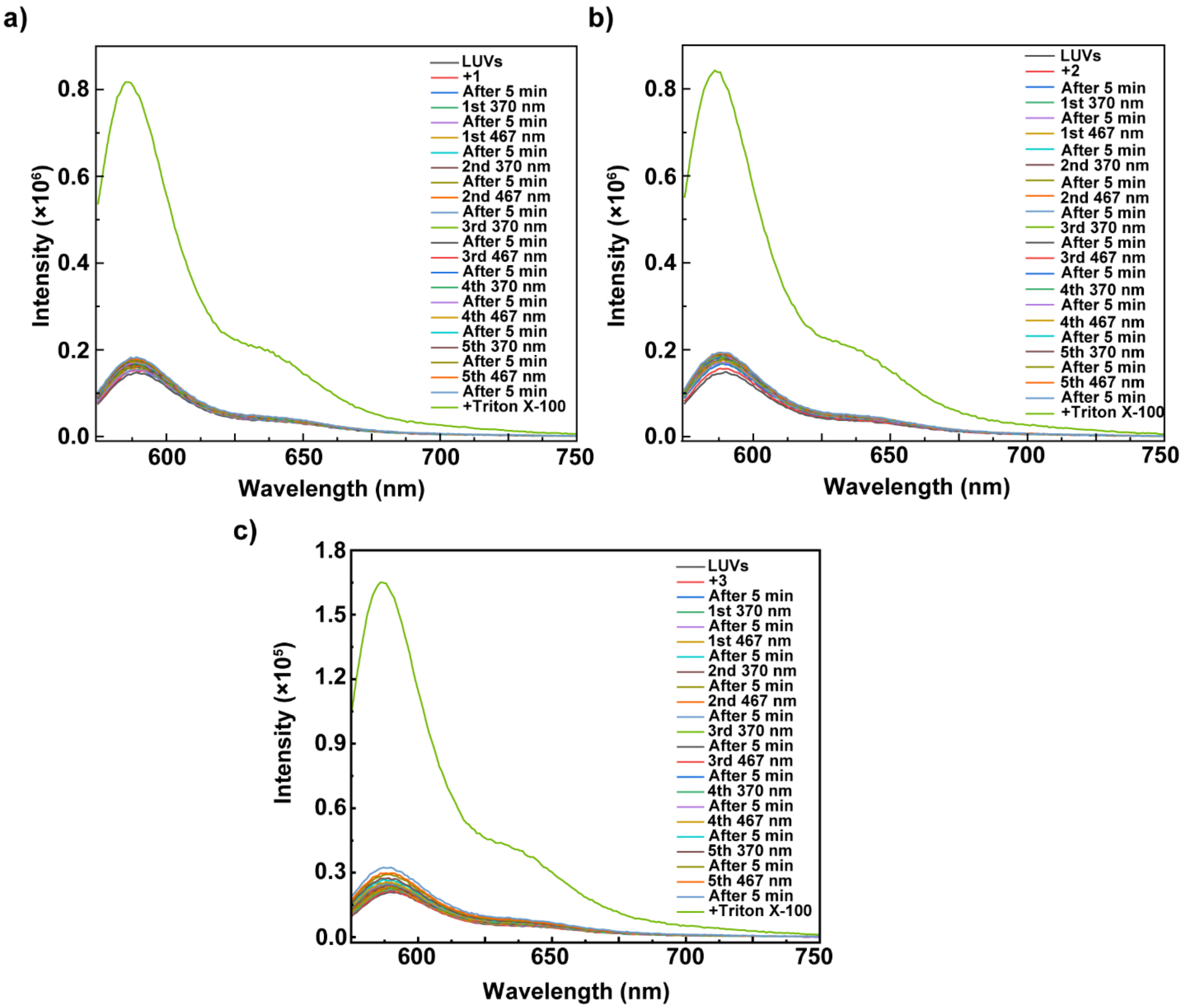

Figure S18. Emission spectra of sulforhodamine B (trial 1) released from EYPC/Chol 6:4 LUVs during five light irradiation cycles after the addition of **a)** rotaxane **1** (10 mol %)**, b)** rotaxane **2** (10 mol %), and **c)** axle **3** (10 mol %) at 25 °C. The LUVs are suspended in a buffer solution containing HEPES buffer (10 mM, pH 7.2) and NaCl (100 mM).

Table S24. Maximum fluorescence emission of sulforhodamine B released from EYPC: Chol 6:4 over five irradiation cycles after the addition of **1**-*E* to LUVs.

Event	Trials (T)			Release %			Mean	Standard Deviation
	T1	T2	T3	T1	T2	T3		
LUVs	148028	144221.4	144120.2	0	0	0	0	0
+**1**-*E*	152624.3	153505.1	150396	0.68701	1.40637	0.89846	0.99728	0.36972
After 5 min	154114.9	155359.6	151824.3	0.9098	1.6873	1.10293	1.23335	0.40483
After 1st irr. @370 nm	154948.1	157159	155421.9	1.03434	1.9599	1.61797	1.5374	0.46801
After 5 min	153492.3	160263.2	157745.6	0.81675	2.43015	1.95064	1.73251	0.82852
After 1st irr. @467 nm	158368.4	164648	158943	1.54558	3.09438	2.12206	2.25401	0.78279
After 5 min	161782.5	165588.3	159608	2.05588	3.23683	2.21727	2.50333	0.64034
After 2nd irr. @370 nm	162094.5	164869	160773.9	2.10251	3.12786	2.38417	2.53818	0.52974
After 5 min	164883.7	166017.5	160797.1	2.51941	3.30184	2.38749	2.73625	0.49424
After 2nd irr. @467 nm	164548.3	170288	164333.3	2.46928	3.94877	2.89374	3.10393	0.76181
After 5 min	167638.7	171853.6	165301	2.93119	4.18595	3.03229	3.38314	0.69709
After 3rd irr. @370 nm	166644.5	173583.9	165933.3	2.78258	4.44806	3.12281	3.45115	0.87995
After 5 min	168224.1	171625.8	166230.6	3.01869	4.15143	3.16537	3.44516	0.61603
After 3rd irr. @ 467 nm	170685.2	173532.4	168041.3	3.38655	4.44026	3.42459	3.75047	0.59768
After 5 min	171066	174794.4	170931	3.44347	4.63144	3.83829	3.97106	0.60501
After 4th irr. @ 370 nm	173126.6	174624	169211.6	3.75146	4.60563	3.59214	3.98307	0.545
After 5 min	172062.1	175235.7	168928.3	3.59235	4.69829	3.55157	3.94741	0.65061
After 4th irr. @ 467 nm	174656.2	177054.5	172930.3	3.98009	4.97381	4.12451	4.35947	0.53691
After 5 min	176036.9	179409	173109.5	4.18646	5.33049	4.15016	4.5557	0.67123
After 5th irr. @ 370 nm	174790.5	178190	171446.8	4.00016	5.14583	3.91213	4.35271	0.68827
After 5 min	177914.3	179895.9	173107.5	4.46706	5.40426	4.14987	4.67373	0.65223
After 5th irr. @ 467 nm	178935.5	180282.6	175130.4	4.61971	5.46283	4.43948	4.84068	0.54629
After 5 min	183501.4	179136.4	179086.8	5.30217	5.2892	5.00589	5.19909	0.16744
+Triton X	817064.9	804341.4	842629.3	100	100	100	100	0.00E+00

Table S25. Maximum fluorescence emission of sulforhodamine B released from EYPC/Chol 6:4 LUVs over five irradiation cycles after the addition of rotaxane **2**.

Event	Trials (T)			Release %			Mean	Standard Deviation
	T1	T2	T3	T1	T2	T3		
LUVs	149346.8	151756.4	155118.5	0	0	0	0	0
+**2**	156827.8	159172.5	157597.4	1.08559	1.07516	0.35415	0.8383	0.41932
After 5 min	167560.7	164987.8	165032.8	2.64307	1.91824	1.41642	1.99258	0.61669
After 1st irr. @370 nm	170887.6	167794.3	168826	3.12584	2.32512	1.95834	2.46977	0.59704
After 5 min	172096.6	171291.5	168782.5	3.30127	2.83213	1.95213	2.69518	0.68492
After 1st irr. @467 nm	177084	173762.5	175782.6	4.025	3.19037	2.9522	3.38919	0.56336
After 5 min	179336.2	174471.5	176612.9	4.35183	3.29316	3.07082	3.57194	0.68449
After 2nd irr. @370 nm	179641.3	175428.7	178360.5	4.3961	3.43194	3.32049	3.71617	0.59146
After 5 min	181094.3	177013	179067.3	4.60696	3.66161	3.42147	3.89668	0.62673
After 2nd irr. @467 nm	182308.8	177255.5	177947.3	4.78318	3.69677	3.26146	3.91381	0.78373
After 5 min	182774.8	176122.3	180578.2	4.85082	3.53249	3.63733	4.00688	0.73275
After 3rd irr. @370 nm	182463.5	177874.3	181671.7	4.80565	3.78648	3.79356	4.12856	0.58638
After 5 min	185433.5	178330.4	181100.9	5.23662	3.85261	3.712	4.26708	0.84258
After 3rd irr. @ 467 nm	186063.7	178513.7	183284.7	5.32807	3.87918	4.024	4.41041	0.798
After 5 min	185943.8	180924.7	182384.3	5.31067	4.22873	3.89535	4.47825	0.73992
After 4th irr. @ 370 nm	185740.6	180908.7	184296.7	5.28118	4.2264	4.16857	4.55872	0.62634
After 5 min	188829.6	180455.5	183628.7	5.72944	4.1607	4.07315	4.65443	0.93201
After 4th irr. @ 467 nm	190406.8	182300.9	187248.3	5.95831	4.42825	4.59025	4.99227	0.84053
After 5 min	190635.3	181913.2	185947	5.99147	4.37203	4.40434	4.92261	0.9258
After 5th irr. @ 370 nm	192322.9	181753.6	184902.5	6.23636	4.3489	4.25513	4.94679	1.11778
After 5 min	191741.8	182428.2	188302.6	6.15204	4.4467	4.74089	5.11321	0.9116
After 5th irr. @ 467 nm	192763	182440.6	187857.6	6.30022	4.44849	4.67731	5.14201	1.00954
After 5 min	193839.7	182474.6	190486.1	6.45647	4.45343	5.05283	5.32091	1.02808
+Triton X	838469.1	841522.4	855074.3	100	100	100	100	0.00E+00

Table S26. Maximum fluorescence emission of sulforhodamine B released from EYPC: Chol 6:4 over five irradiation cycles after the addition of axle **3** to LUVs.

Event	Trials (T)			Release %			Mean	Standard Deviation
	T1	T2	T3	T1	T2	T3		
LUVs	20791.8	20419.01	22488.95	0	0	0	0	0
+**2**	21537.48	21105.29	22916.74	0.51666	0.47987	0.24774	0.41475	0.14581
After 5 min	22104.5	21928.83	23304	0.90952	1.05572	0.472	0.81241	0.30373
After 1st irr. @370 nm	22218.59	22540.55	23481.77	0.98857	1.48345	0.57495	1.01566	0.45486
After 5 min	22308.4	22552.13	23665.34	1.0508	1.49155	0.68126	1.07454	0.40567
After 1st irr. @467 nm	22435.93	23050.63	24255.41	1.13916	1.84012	1.02297	1.33408	0.44207
After 5 min	22716.78	23296.52	24984.08	1.33375	2.01205	1.44495	1.59692	0.36379
After 2nd irr. @370 nm	23094.66	23562.61	25164.21	1.59557	2.19811	1.54927	1.78098	0.36198
After 5 min	23128.1	23875.22	25236.54	1.61874	2.41669	1.59115	1.87553	0.46887
After 2nd irr. @467 nm	23288.09	23890.56	25466.99	1.72959	2.42742	1.72461	1.96054	0.40434
After 5 min	23731.02	23967.56	25751.67	2.03648	2.48126	1.88947	2.13574	0.30813
After 3rd irr. @370 nm	24069.96	24383.34	26166.83	2.27133	2.77199	2.12989	2.39107	0.33738
After 5 min	24247.43	24497.3	26726.92	2.39429	2.85167	2.45424	2.56673	0.24858
After 3rd irr. @ 467 nm	24149.74	24650.23	27392.31	2.3266	2.95861	2.83958	2.70826	0.33584
After 5 min	24743.43	24928.13	28436.47	2.73795	3.15293	3.44426	3.11171	0.35495
After 4th irr. @ 370 nm	24885.3	25339.36	29839.34	2.83624	3.44047	4.25667	3.51113	0.71285
After 5 min	25499.35	25681.24	31883.87	3.2617	3.67952	5.44068	4.1273	1.15644
After 4th irr. @ 467 nm	25647.06	26725.25	33870.73	3.36404	4.40953	6.59128	4.78828	1.64662
After 5 min	26867.05	26931.17	37281.07	4.20933	4.55352	8.56624	5.77636	2.42222
After 5th irr. @ 370 nm	27535.46	27912.19	39565.17	4.67245	5.23948	9.88898	6.6003	2.86215
After 5 min	29208.08	29347.58	44007.89	5.83135	6.24315	12.4618	8.17877	3.71492
After 5th irr. @ 467 nm	29833.78	30073.44	46264.83	6.26488	6.75069	13.7688	8.92812	4.19918
After 5 min	32235.04	32290.29	49324.13	7.92862	8.30078	15.54047	10.58996	4.2913
+Triton X	165119.9	163432.9	195168.3	100	100	100	100	0.00E+00

S2.3.4. Dye release studies in DPPC LUVs

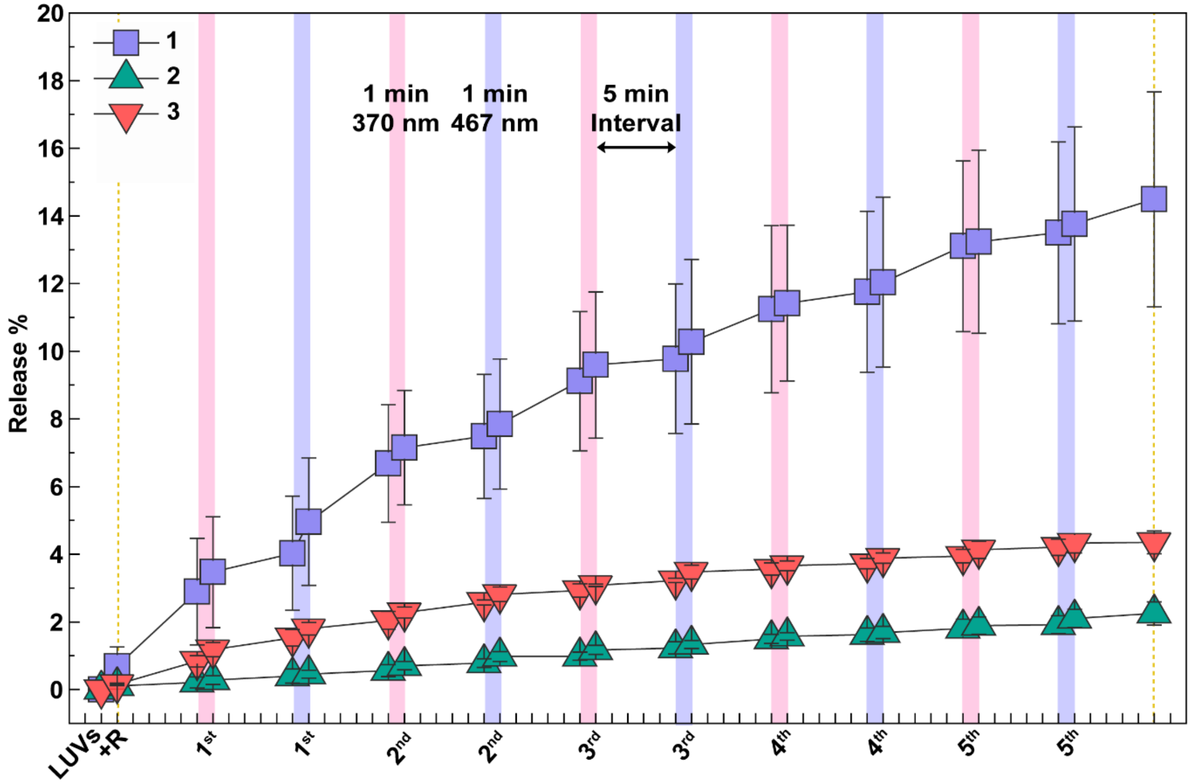

Figure S19. Percentage of sulforhodamine B released from DPPC LUVs upon five irradiation cycles after the addition of rotaxane **1,** rotaxane **2**, and axle **3** in 10 mol % at 25 °C. The DPPC LUVs are suspended in a buffer solution containing HEPES buffer (10 mM, pH 7.2) and NaCl (100 mM).

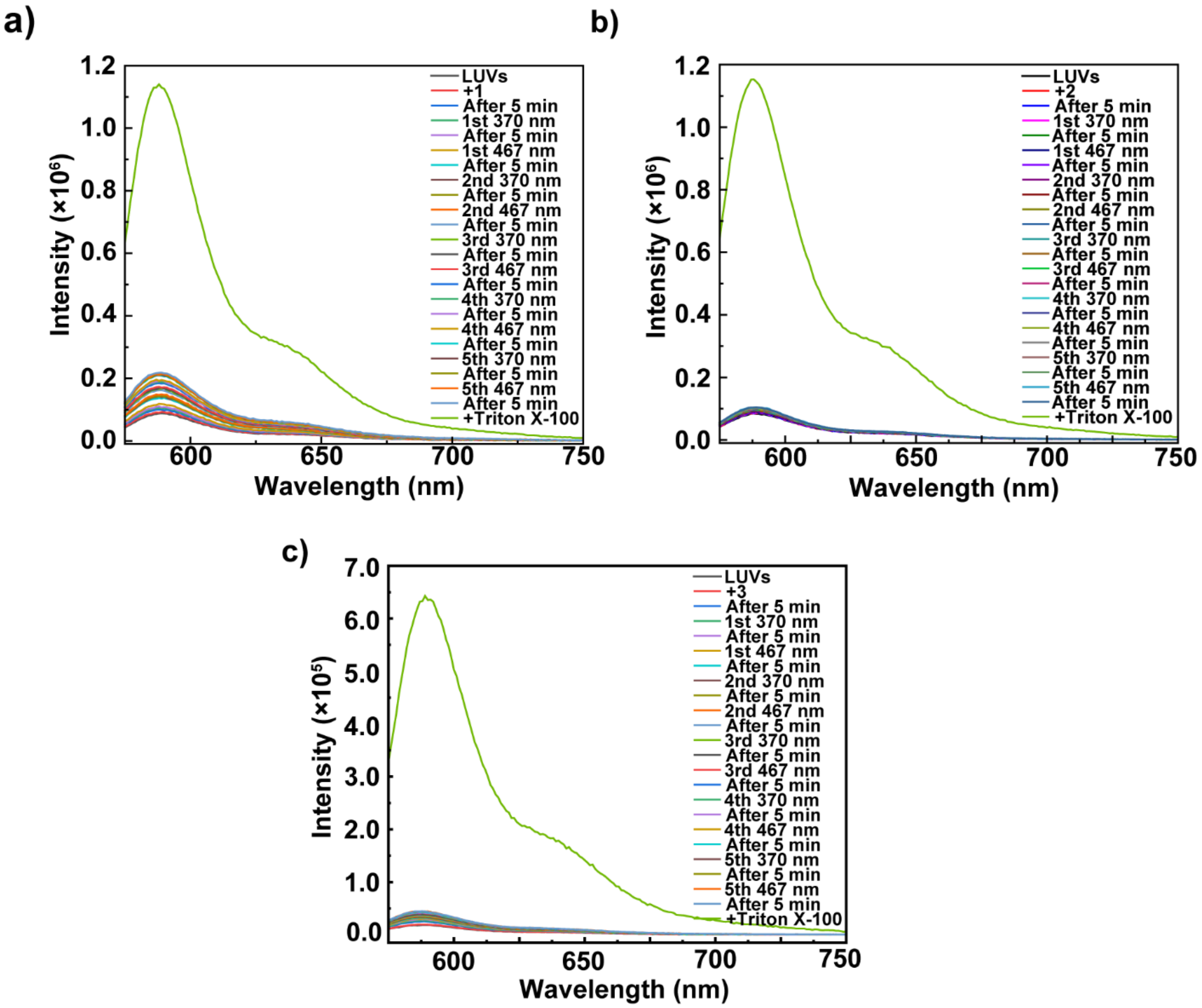

Figure S20. Emission spectra of sulforhodamine B (trial 1) released from DPPC LUVs during five light irradiation cycles after the addition of **a)** rotaxane **1** (10 mol %)**, b)** rotaxane **2** (10 mol %), and **c)** axle **3** (10 mol %) at 25 °C. The LUVs are suspended in a buffer solution containing HEPES buffer (10 mM, pH 7.2) and NaCl (100 mM)

Table S27. Maximum fluorescence emission of sulforhodamine B released from DPPC LUVs over five irradiation cycles after the addition of **1**-*E* to LUVs.

Event	Trials (T)			Release %			Mean	Standard Deviation
	T1	T2	T3	T1	T2	T3		
LUVs	88139.13	95570.16	87072.38	0	0	0	0	0
+**1**-*E*	92946.98	98462.02	100934.7	0.45693	0.27774	1.34498	0.69322	0.57151
After 5 min	100062.3	130863.3	129920.1	1.13315	3.38964	4.15724	2.89335	1.57195
After 1st irr. @370 nm	105494.9	136487.9	136933.7	1.64945	3.92984	4.83774	3.47234	1.64264
After 5 min	110338.2	144751.7	141271.5	2.10976	4.72352	5.2586	4.03063	1.6849
After 1st irr. @467 nm	118245.7	152985.4	154215	2.86127	5.5143	6.51443	4.96334	1.88787
After 5 min	137622.8	178235.4	163514.4	4.70283	7.93938	7.41669	6.6863	1.7375
After 2nd irr. @370 nm	143343.4	183649.8	166869.2	5.24651	8.45939	7.74219	7.14936	1.68649
After 5 min	145063.4	188466	170737.9	5.40997	8.92195	8.11754	7.48316	1.83993
After 2nd irr. @467 nm	148695.6	194853.4	172273.4	5.75517	9.53541	8.26653	7.85237	1.92385
After 5 min	162150.4	211742.3	181435.5	7.03388	11.15747	9.15547	9.11561	2.06208
After 3rd irr. @370 nm	168410.1	219645	182287.4	7.6288	11.91646	9.23812	9.59446	2.16593
After 5 min	170143.2	222221.2	183733.3	7.79351	12.16389	9.3784	9.7786	2.2125
After 3rd irr. @ 467 nm	173034.3	229745.4	188905.3	8.06827	12.88653	9.88021	10.27834	2.43367
After 5 min	184238.7	240938.9	196536.5	9.13311	13.96158	10.62062	11.23844	2.47281
After 4th irr. @ 370 nm	188880.9	241386.7	197200.6	9.5743	14.00459	10.68506	11.42131	2.30509
After 5 min	193658.9	246174.2	198063.2	10.02839	14.46439	10.76875	11.75384	2.37641
After 4th irr. @ 467 nm	194669.8	250645.3	201549.7	10.12447	14.89381	11.10702	12.04177	2.51833
After 5 min	209748.6	262356.9	208094.1	11.55752	16.01862	11.74198	13.10604	2.52405
After 5th irr. @ 370 nm	210812.1	265901.2	207529.9	11.6586	16.35902	11.68724	13.23495	2.70556
After 5 min	213859.9	268429.5	210297.6	11.94825	16.60184	11.95578	13.50196	2.68458
After 5th irr. @ 467 nm	214227.6	273329.3	213242	11.9832	17.07244	12.24145	13.7657	2.86663
After 5 min	218513.1	284476	220447.4	12.39048	18.143	12.94055	14.49134	3.17436
+Triton X	1140349	1136776	1117748	100	100	100	100	8.20E-15

Table S28. Maximum fluorescence emission of sulforhodamine B released from DPPC LUVs over five irradiation cycles after the addition of rotaxane **2**.

Event	Trials (T)			Release %			Mean	Standard Deviation
	T1	T2	T3	T1	T2	T3		
LUVs	84598.77	82709.15	87381.89	0	0	0	0	0
+**2**	86124.18	84509.39	87463.67	0.14286	0.16938	0.00757	0.10661	0.08678
After 5 min	88365.82	85276.84	87760.84	0.3528	0.24159	0.0351	0.20983	0.16122
After 1st irr. @370 nm	87585.38	87129.41	89046.91	0.27971	0.4159	0.15421	0.28327	0.13088
After 5 min	89754.38	88649.98	89096.17	0.48284	0.55897	0.15877	0.40019	0.21251
After 1st irr. @467 nm	90134.63	88445.6	90891.56	0.51845	0.53974	0.32506	0.46108	0.11828
After 5 min	90745.02	90477.81	91478.91	0.57562	0.73095	0.37945	0.56201	0.17614
After 2nd irr. @370 nm	91775.98	91704.86	93893.29	0.67217	0.8464	0.60307	0.70721	0.12539
After 5 min	92698.21	92501.83	94561.77	0.75854	0.92139	0.66498	0.78164	0.12975
After 2nd irr. @467 nm	95229.12	94503.02	96288.23	0.99557	1.10968	0.82488	0.97671	0.14333
After 5 min	95268.56	94503.02	96612.34	0.99927	1.10968	0.8549	0.98795	0.12776
After 3rd irr. @370 nm	97242.12	96578.48	98523.22	1.1841	1.30495	1.03188	1.17364	0.13684
After 5 min	97299.55	97856.13	98933.02	1.18948	1.42517	1.06983	1.22816	0.1808
After 3rd irr. @ 467 nm	98218.99	98265.26	100856.8	1.27559	1.46366	1.24801	1.32909	0.11736
After 5 min	100581.9	100151.2	102113.9	1.49688	1.64111	1.36444	1.50081	0.13838
After 4th irr. @ 370 nm	100875.7	100815.1	103450.2	1.52439	1.70358	1.48821	1.57206	0.11533
After 5 min	101362.5	102314.2	103103.4	1.56999	1.84462	1.45608	1.62357	0.19973
After 4th irr. @ 467 nm	101745.3	102814.3	104256.2	1.60584	1.89168	1.56285	1.68679	0.17874
After 5 min	101887.2	105229	105513.8	1.61913	2.11888	1.67933	1.80578	0.27282
After 5th irr. @ 370 nm	102864.9	105929.3	106626.3	1.7107	2.18477	1.78236	1.89261	0.25554
After 5 min	103288	106303.7	106644.6	1.75032	2.22	1.78406	1.91813	0.26197
After 5th irr. @ 467 nm	104529.1	108184.6	109389.3	1.86655	2.39696	2.03827	2.10059	0.27064
After 5 min	104407.6	109176	113299.9	1.85518	2.49024	2.40045	2.24862	0.34368
+Triton X	1152361	1145530	1167095	100	100	100	100	0.00E+00

Table S29. Maximum fluorescence emission of sulforhodamine B released from DPPC LUVs over five irradiation cycles after the addition of axle **3** to LUVs.

Event	Trials (T)			Release %			Mean	Standard Deviation
	T1	T2	T3	T1	T2	T3		
LUVs	18537.18	19038.32	19126.9	0	0	0	0	0
+**3**	19399.54	19993.29	20162.93	0.13812	0.15304	0.16259	0.15125	0.01233
After 5 min	24962.31	23996.3	23589.55	1.02906	0.79457	0.70033	0.84132	0.16928
After 1st irr. @370 nm	27157.19	26487.51	25253.63	1.3806	1.19382	0.96147	1.17863	0.20998
After 5 min	29518.8	28812.03	27257.94	1.75884	1.56634	1.27601	1.53373	0.24306
After 1st irr. @467 nm	30720.21	30599.71	29229.69	1.95126	1.85284	1.58544	1.79651	0.1893
After 5 min	32465.19	32578.38	30235.11	2.23074	2.16995	1.74322	2.04797	0.26566
After 2nd irr. @370 nm	33464.21	33700.88	32478.5	2.39075	2.34984	2.09528	2.27862	0.16009
After 5 min	35022.58	35223.47	34992.44	2.64034	2.59385	2.4898	2.57466	0.07708
After 2nd irr. @467 nm	36941.37	37345.87	35508.11	2.94766	2.93399	2.57072	2.81746	0.21379
After 5 min	37129.93	38369.61	36403.69	2.97786	3.09805	2.71127	2.92906	0.19796
After 3rd irr. @370 nm	37985.91	38158.35	38617.08	3.11495	3.0642	3.05861	3.07926	0.03104
After 5 min	38435.85	39604.86	39479.3	3.18702	3.29602	3.19392	3.22565	0.06104
After 3rd irr. @ 467 nm	41488.34	40732.27	39934.68	3.67591	3.4767	3.26539	3.47266	0.20529
After 5 min	42011.81	41016.21	40820.33	3.75975	3.5222	3.40437	3.56211	0.18102
After 4th irr. @ 370 nm	42266.51	41928.65	41485.79	3.80054	3.66843	3.5088	3.65926	0.14609
After 5 min	42271.86	42857.64	41762.91	3.8014	3.81731	3.55229	3.72367	0.14863
After 4th irr. @ 467 nm	43328.53	43778.64	42735.93	3.97064	3.96491	3.70499	3.88018	0.15175
After 5 min	43082.17	44932.16	43002.27	3.93118	4.14978	3.74679	3.94258	0.20174
After 5th irr. @ 370 nm	44137.57	46534.37	43967.82	4.10022	4.40655	3.89831	4.13503	0.2559
After 5 min	44417.2	46962.41	44630.81	4.145	4.47514	4.00236	4.2075	0.24251
After 5th irr. @ 467 nm	44722.89	48084.68	45445.94	4.19396	4.655	4.13027	4.32641	0.28634
After 5 min	44620.76	48595.31	45507.53	4.17761	4.73684	4.13994	4.35146	0.33428
+Triton X	642903.9	643020.1	656349.4	100	100	100	100	0.00E+00

S2.3.5. Effect of varying rotaxane 1 concentrations on sulforhodamine B release from LUVs with light irradiation

To assess whether different concentrations of Rotaxane **1** can influence the highly fluid pure EYPC LUVs and EYPC/Chol 8:2 LUVs, Sulforhodamine B release after five irradiation cycles was examined following the same procedure described in section **S2.2**, using 5 mol % of rotaxane **1**. Rotaxane **2** was also tested as a control using the same method.

Table S30. Sulforhodamine B release from LUVs upon addition of different concentrations of rotaxanes **1** and **2**

Rotaxane mol %	Dye release %			
	EYPC		EYPC/Chol 8:2	
	1	2	1	2
5	60 (±4.30)	74 (±6.00)	28 (±2.34)	16 (±2.20)
10	70 (±3.76)	65 (±5.25)	59 (±4.17)	26 (±3.32)

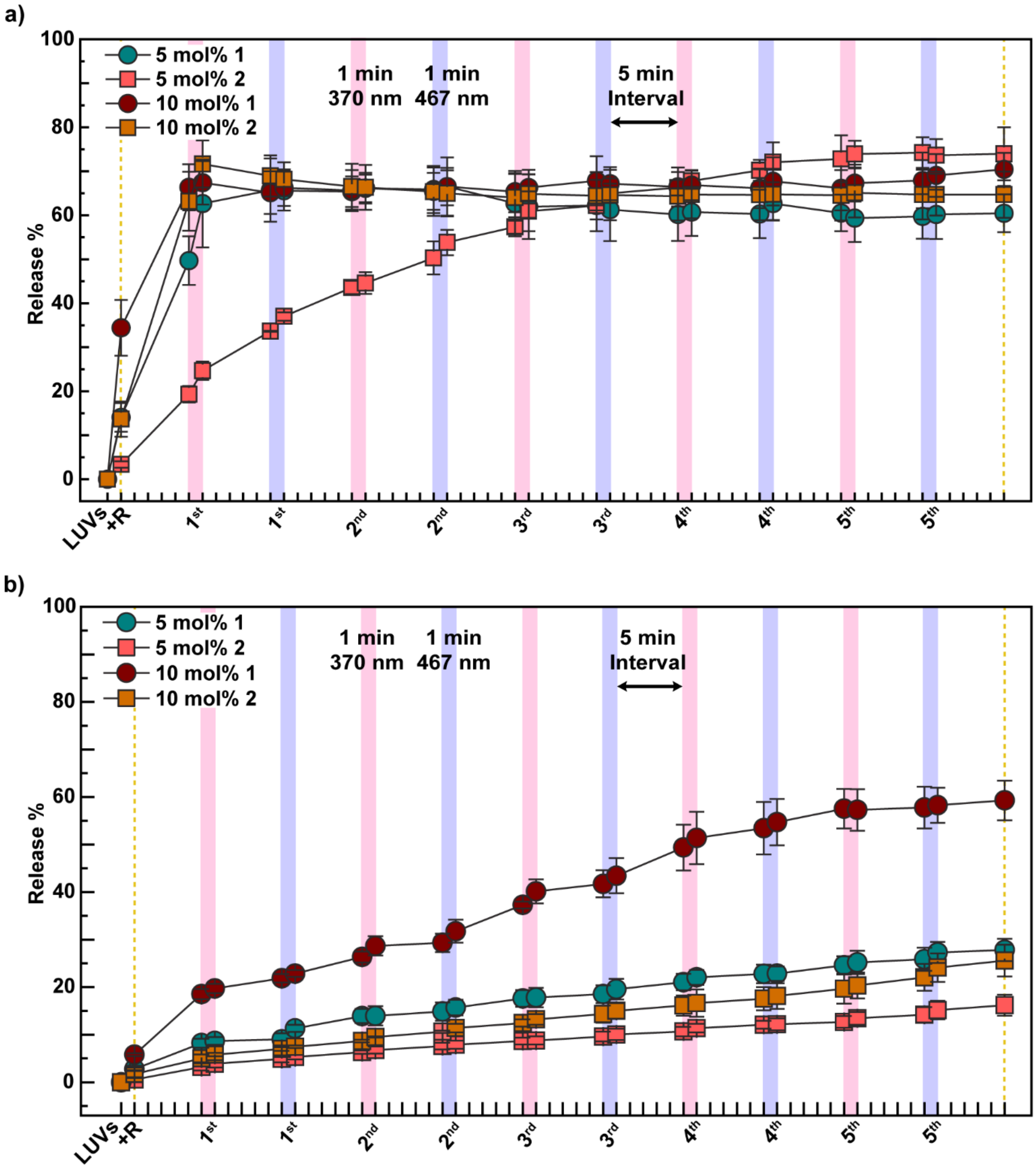

Figure S21: Percentage of sulforhodamine B released from LUVs containing rotaxane **1** at different concentrations (5 mol % and 10 mol %) from **a)** EYPC LUVs and **b)** EYPC/Chol 8:2 LUVs upon five alternating light-irradiation cycles. The fluorescent emission of sulforhodamine B was measured before and after 1-minute irradiation with 370 nm light (pink strips) or 467 nm light (blue strips), and after 5 minutes post-irradiation. The sulforhodamine B (10 mM) was encapsulated in LUVs composed of different lipid/cholesterol ratios suspended in a solution of HEPES buffer (10 mM, pH 7.2). rotaxane **1** (5 mol % or 10 mol % with respect to the total lipid concentration) dissolved in DMSO was added to the LUVs

Table S31. Maximum fluorescence emission of sulforhodamine B release from EYPC LUVs over five irradiation cycles after the addition of 5 mol % **1-*E*** to LUVs.

Event	Trials (T)			Release %			Mean	Standard Deviation
	T1	T2	T3	T1	T2	T3		
LUVs	66926.91	59991.34	60755.21	0	0	0	0	0
+**1**-*E*	97422.56	78646.66	84546.19	17.74997	11.5158	12.99354	14.08643	3.25761
After 5 min	145613.9	136704.4	163330.4	45.79968	47.35443	56.02183	49.72531	5.50808
After 1st irr. @370 nm	194409.8	151961.3	165118.9	74.2013	56.77238	56.99862	62.65743	9.99792
After 5 min	193699.6	162718.3	170548.3	73.78791	63.41259	59.96391	65.72147	7.19541
After 1st irr. @467 nm	188161	164668.4	173586.8	70.56417	64.61641	61.62341	65.60133	4.55103
After 5 min	179621.7	171328.4	173935	65.59389	68.72753	61.81361	65.37834	3.462
After 2nd irr. @370 nm	178344.6	172869.5	177691.4	64.85053	69.67887	63.86516	66.13152	3.11135
After 5 min	174974.7	176679.9	175535.9	62.88908	72.031	62.68791	65.86933	5.33711
After 2nd irr. @467 nm	172045.8	179730.1	178724.8	61.18435	73.91385	64.42955	66.50925	6.61468
After 5 min	163293.4	173116.8	174588.2	56.09002	69.8315	62.1703	62.69727	6.88588
After 3rd irr. @370 nm	161698.2	172916.1	172447.7	55.16156	69.70763	61.00128	61.95682	7.31996
After 5 min	165335.1	171233.1	171863.4	57.27841	68.66874	60.68215	62.20977	5.8468
After 3rd irr.@ 467 nm	163394.9	172466.6	167421.2	56.14909	69.43014	58.25605	61.27843	7.13776
After 5 min	164118.7	168679.9	164921.7	56.57037	67.09265	56.89093	60.18465	5.98465
After 4th irr.@ 370 nm	164512.8	168344.2	167929	56.79978	66.88542	58.53339	60.73953	5.39262
After 5 min	164746.2	167980.5	165748.6	56.9356	66.66091	57.34253	60.31301	5.5012
After 4th irr.@ 467 nm	169812.1	168665	172468.7	59.88422	67.08344	61.01275	62.66014	3.87203
After 5 min	168172.6	165741.7	165873.8	58.92996	65.27894	57.41093	60.53994	4.17378
After 5th irr.@ 370 nm	165992.6	165878.4	161398	57.66109	65.36329	54.96646	59.33028	5.39566
After 5 min	166347.3	166033.5	163022.3	57.86754	65.45908	55.85355	59.72672	5.06547
After 5th irr.@ 467 nm	163329.2	167904	166782.6	56.11085	66.61373	57.90725	60.21061	5.61754
After 5 min	163336.6	164810.2	171714.9	56.11514	64.70391	60.60108	60.47338	4.29581
+Triton X	238733.8	221989	243853.8	100	100	100	100	0.00E+00

Table S32. Maximum fluorescence emission of sulforhodamine B release from EYPC LUVs over five irradiation cycles after the addition of 5 mol % rotaxane **2** to LUVs.

Event	Trials (T)			Release %			Mean	Standard Deviation
	T1	T2	T3	T1	T2	T3		
LUVs	63252.77	61571.16	65450.7	0	0	0	0	0
+**2**	68652.55	69520.58	70194.17	3.14164	4.19659	2.74823	3.36216	0.74894
After 5 min	100163.8	96655.91	96497.1	21.47517	18.52166	17.98742	19.32808	1.87852
After 1st irr. @370 nm	109508	104818.2	107316.2	26.91172	22.83062	24.25572	24.66602	2.07125
After 5 min	120815.5	125368.2	123730.4	33.49054	33.67924	33.76565	33.64514	0.14069
After 1st irr. @467 nm	126317.6	130231.6	131204.9	36.69167	36.24665	38.09616	37.01149	0.96534
After 5 min	138732.9	141043.8	142975.1	43.91504	41.95455	44.91546	43.59501	1.50617
After 2nd irr. @370 nm	142567.7	140664.4	144666.3	46.14614	41.75425	45.89528	44.59856	2.46643
After 5 min	155007.7	149000.9	154179.1	53.38386	46.15523	51.40676	50.31528	3.73588
After 2nd irr. @467 nm	158424.3	157159.4	161470.5	55.37165	50.46219	55.6312	53.82168	2.9123
After 5 min	162982.3	165717.7	167596.2	58.02356	54.98021	59.18025	57.39467	2.1695
After 3rd irr. @370 nm	166959.7	168989.9	178802	60.33765	56.70762	65.67257	60.90595	4.50941
After 5 min	171281.8	177075.3	174027.5	62.85224	60.97602	62.90638	62.24488	1.0992
After 3rd irr.@ 467 nm	175480.7	180291.1	181170.7	65.29524	62.67366	67.04491	65.0046	2.20007
After 5 min	178116	183368.7	182313.2	66.82845	64.29838	67.70684	66.27789	1.76967
After 4th irr.@ 370 nm	181227.6	186134.1	184511.8	68.63883	65.75828	68.9807	67.7926	1.77005
After 5 min	183517.8	190344	190935.3	69.97126	67.98073	72.70228	70.21809	2.37043
After 4th irr.@ 467 nm	182564.1	193132.3	198755	69.41643	69.45268	77.23277	72.03396	4.50234
After 5 min	182806.7	194166	201733.4	69.55758	69.99842	78.95837	72.83812	5.30487
After 5th irr.@ 370 nm	188606.2	196984.8	198895.9	72.93176	71.48649	77.31442	73.91089	3.03483
After 5 min	188571	197279.9	200436.4	72.91125	71.64225	78.20697	74.25349	3.48211
After 5th irr.@ 467 nm	192660	193073.4	197084.3	75.29028	69.42162	76.26484	73.65891	3.70182
After 5 min	188669.7	191390.5	204258.6	72.96869	68.53317	80.42143	73.97443	6.00761
+Triton X	235130.5	250996.7	238051.4	100	100	100	100	0

Table S33. Maximum fluorescence emission of sulforhodamine B released from EYPC/Chol 8:2 LUVs over five irradiation cycles after the addition of 5 mol % **1-***E* to LUVs.

Event	Trials (T)			Release %			Mean	Standard Deviation
	T1	T2	T3	T1	T2	T3		
LUVs	246232.3	255522.5	264039.5	0	0	0	0	0
+**1-***E*	267347.1	274267.7	288726.8	2.74121	2.39258	3.11965	2.75115	0.36364
After 5 min	317941.6	315961.1	324543.6	9.30961	7.71418	7.64568	8.22316	0.94152
After 1st irr. @370 nm	320404.4	319356.6	330073.3	9.62934	8.14757	8.34445	8.70712	0.80471
After 5 min	326764.7	323075.7	327639.5	10.45507	8.62226	8.0369	9.03808	1.26157
After 1st irr. @467 nm	339927.9	343366.7	348383.3	12.16397	11.21214	10.65821	11.34478	0.76159
After 5 min	366502.3	361332.8	364403.1	15.61398	13.50527	12.68257	13.93394	1.51199
After 2nd irr. @370 nm	371138.7	360464.3	361513.8	16.2159	13.39442	12.31746	13.97593	2.01322
After 5 min	377217.9	368443	368676.5	17.00513	14.41279	13.22259	14.88017	1.9341
After 2nd irr. @467 nm	379438.6	379908	374537.3	17.29342	15.87615	13.96319	15.71092	1.67125
After 5 min	395811.3	393404.1	389525.2	19.419	17.59874	15.85716	17.62496	1.78107
After 3rd irr. @370 nm	398976.9	396202	388451.6	19.82998	17.95586	15.7215	17.83578	2.05687
After 5 min	402554.2	402138.3	395272.3	20.29439	18.71354	16.5834	18.53044	1.86226
After 3rd irr.@ 467 nm	412460.3	411672	400827.7	21.58045	19.9304	17.28541	19.59875	2.16664
After 5 min	422063.9	420225.7	417099.6	22.82724	21.02216	19.34163	21.06367	1.74318
After 4th irr.@ 370 nm	429674	425177.1	428266.6	23.81521	21.65414	20.75276	22.07404	1.57381
After 5 min	437715.6	432261.2	429887.4	24.8592	22.55833	20.95758	22.7917	1.96125
After 4th irr.@ 467 nm	434226.6	434050.7	433213	24.40625	22.78673	21.37783	22.85694	1.51543
After 5 min	450890.5	447383.8	443729.9	26.56963	24.48853	22.7068	24.58832	1.93335
After 5th irr.@ 370 nm	458840.8	453442.6	443925.1	27.60178	25.26185	22.73146	25.19836	2.43578
After 5 min	466526	453442.6	452708.3	28.5995	25.26185	23.84137	25.90091	2.44259
After 5th irr.@ 467 nm	473657.1	469941.7	462293.8	29.52529	27.36775	25.05265	27.31523	2.23679
After 5 min	473657.1	481596.8	463255.5	29.52529	28.85537	25.17418	27.85161	2.34279
+Triton X	1016503	1038997	1055390	100	100	100	100	7.11E-15

Table S34. Maximum fluorescence emission of sulforhodamine B released from EYPC/Chol 8:2 LUVs over five irradiation cycles after the addition of 5 mol % of rotaxane **2** to LUVs.

Event	Trials (T)			Release %			Mean	Standard Deviation
	T1	T2	T3	T1	T2	T3		
LUVs	269753.2	274380.1	276467.3	0	0	0	0	0
+**2**	272880.9	282751.4	278168.9	0.40079	1.1334	0.22747	0.58722	0.48088
After 5 min	292713.5	304548.6	296397.5	2.9422	4.08455	2.6642	3.23031	0.75274
After 1st irr. @370 nm	301523.9	307927.2	299368.8	4.07119	4.54199	3.06139	3.89152	0.75647
After 5 min	306269.7	316649.2	308861.1	4.67932	5.72287	4.33029	4.91083	0.72458
After 1st irr. @467 nm	309962.9	317422.3	313403.1	5.15258	5.82754	4.93745	5.30586	0.46442
After 5 min	318338.9	323985.4	321830.8	6.22591	6.71613	6.06404	6.33536	0.33954
After 2nd irr. @370 nm	319835.4	330494.2	323738.4	6.41767	7.59736	6.31904	6.77803	0.71128
After 5 min	326969.9	336720.6	329523	7.33191	8.44036	7.09231	7.62153	0.71918
After 2nd irr. @467 nm	325433.2	339655.3	334791.5	7.13499	8.8377	7.79658	7.92309	0.85838
After 5 min	332727.9	344323.5	339848.4	8.06976	9.46973	8.47258	8.67069	0.72071
After 3rd irr. @370 nm	332723.7	346288.4	340669.7	8.06921	9.73576	8.58236	8.79578	0.85352
After 5 min	336595.5	353038.6	348291.9	8.56536	10.64967	9.60127	9.60544	1.04217
After 3rd irr.@ 467 nm	340192.6	358363.7	349915.5	9.0263	11.37065	9.81832	10.07175	1.19254
After 5 min	346420.7	361365	354419.6	9.82438	11.77699	10.42041	10.67393	1.00069
After 4th irr.@ 370 nm	361834.7	363124.8	353805.2	11.79958	12.01526	10.33827	11.38437	0.91235
After 5 min	367847.4	367356.8	359794.8	12.57007	12.58824	11.13894	12.09908	0.83156
After 4th irr.@ 467 nm	358441.9	375246.7	362843.3	11.36482	13.65646	11.54646	12.18925	1.27389
After 5 min	362230.4	378776.3	367063.9	11.85029	14.13433	12.11065	12.69842	1.25033
After 5th irr.@ 370 nm	369885.9	384961.5	370328.4	12.83128	14.97176	12.54704	13.45003	1.3255
After 5 min	375558.2	393036.8	373864.1	13.55815	16.06508	13.01968	14.2143	1.62527
After 5th irr.@ 467 nm	379549.5	403119.1	381653.5	14.06961	17.43014	14.06094	15.1869	1.94271
After 5 min	384335.1	412722.2	390184.7	14.68284	18.73031	15.20136	16.20484	2.20244
+Triton X	1050132	1012980	1024541	100	100	100	100	7.11E-15

S2.4. Temperature-dependent sulforhodamine B release from DPPC LUVs

S2.4.1. Sulforhodamine B release from DPPC LUVs at 25 °C without light irradiation

To evaluate the individual effects of **1**-*Z*, rotaxane **2**, and axle **3** on the permeability of DPPC LUVs at 25 °C, the percentage of sulforhodamine B released in every 5 minutes over a total of 15 minutes was measured. Initially, 2946 µL of an extravesicular buffer (10 mM HEPES, 100 mM NaCl, pH 7.2) with 48 µL of LUVs encapsulating 12.5 mM sulforhodamine B were combined in a quartz cuvette. The mixture was placed in the fluorometer chamber and stirred at 150 rpm for 2 minutes to ensure homogeneity. We recorded the emission spectrum from 575 nm to 750 nm, using an excitation wavelength of 565 nm. All fluorescence measurements were performed at 25 °C, with the mixture continuously stirred at 150 rpm during spectrum recordings. Next, 6 µL of a DMSO solution containing 10 mM of the respective compound was added to the cuvette, and the emission spectrum was recorded. Fluorescence was measured every 5 minutes for 15 minutes. Finally, Triton X-100 was added, and the mixture was stirred for 8 minutes to lyse the liposomes, followed by another spectrum recording. The percentage of sulforhodamine release was calculated according to equation S1.

S2.4.2. Sulforhodamine B release from DPPC LUVs at 25 °C with light irradiation

To evaluate the photoswitching effect of **1**-*Z*→**1**-*E*, the same procedure described in section **S2.4.1** was followed up to measurements taken after the addition of the target compound and the subsequent remeasurement after 5 minutes at 25 °C. Next, the cuvette was irradiated at 467 nm for 1 minute, and the emission spectrum was measured immediately afterward. The emission was then remeasured after 4 minutes and again after 5 minutes, totaling 15 minutes of study. Finally, Triton X-100 was added, and the mixture was stirred for 8 minutes to lyse the liposomes, followed by another spectrum recording. The percentage of sulforhodamine B release was calculated according to equation S1.

S2.4.3. Sulforhodamine B release from DPPC LUVs at 45 °C without light irradiation

To evaluate the individual effects of **1**-*Z*, rotaxane **2**, and axle **3** on the permeability of DPPC LUVs at 45 °C, the procedure described in Section **S2.4.1** was followed up to measurements taken after the addition of the target compound and the subsequent remeasurement after 5 minutes at 25 °C. Afterwards, the cuvette was placed in a 45 °C water bath for 5 minutes, and emission was measured again. This process was repeated twice. Finally, Triton X-100 was added, and the mixture was stirred for 8 minutes to lyse the liposomes, followed by another spectrum recording. The percentage of sulforhodamine B release was calculated according to equation S1.

S2.4.4. Sulforhodamine B release from DPPC LUVs at 45 °C with light irradiation

To evaluate the photoswitching effect of **1**-*Z*→ **1**-*E* on the permeability of DPPC LUVs at 45 °C, the same procedure described in section **S2.4.2** was followed, up to the measurement taken after irradiation at 467 nm for 1 minute at 25 °C. Then, the cuvette was placed in a 45 °C water bath for 4 and 5 minutes, respectively, and the emission was measured after each warming period. Finally, Triton X-100 was added, and the mixture was stirred for 8 minutes to lyse the liposomes, followed by another spectrum recording. The percentage of sulforhodamine B release was calculated according to equation S1.

Table S35. Sulforhodamine B release from DPPC LUVs at 25 °C and 45 °C

**Temperature °C**	**Dye release %**			
	**No light irradiation**			**light irradiation**
	**1-*Z***	**2**	**3**	**1 (*Z*→*E*)**
25	3 (±0.30)	2 (±0.12)	1 (±0.22)	5 (±0.97)
45	15 (±1.13)	11 (±0.18)	13 (±0.26)	20 (±1.97)

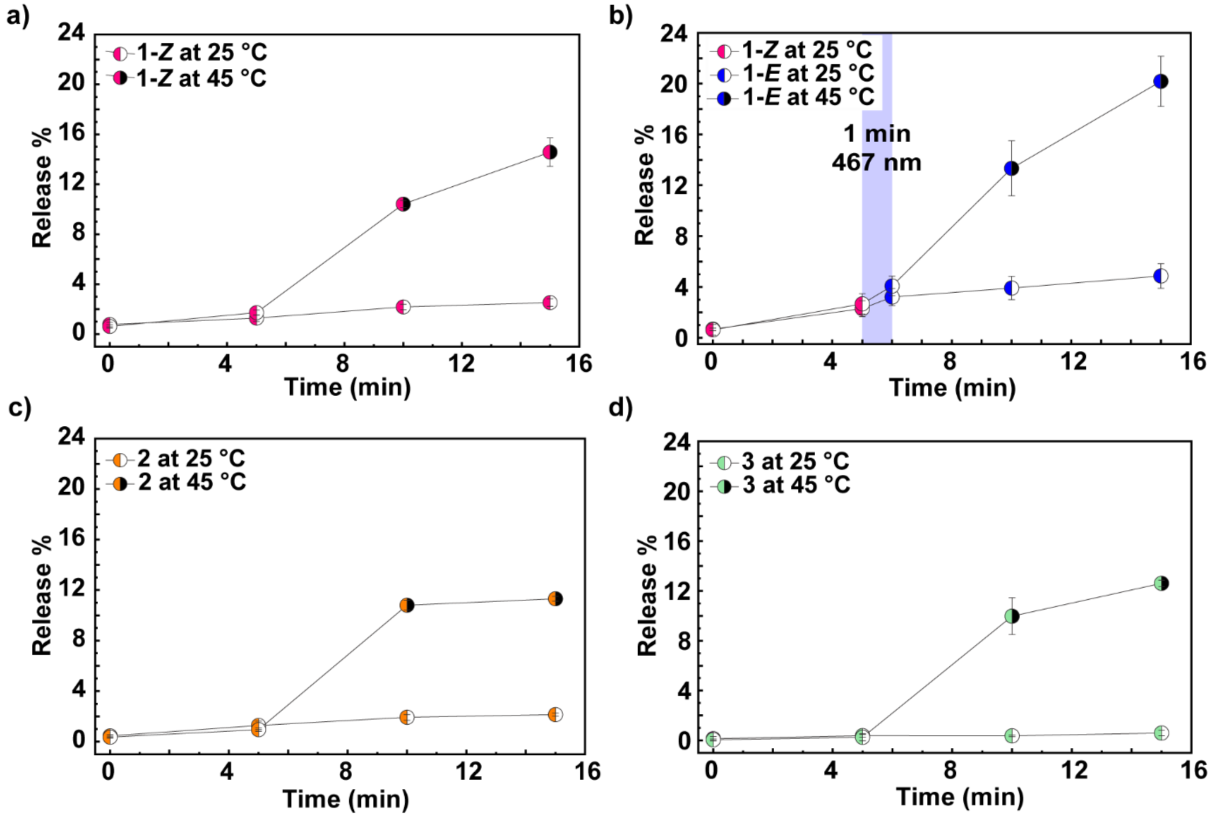

Figure S22: Percentage of sulforhodamine B released over time after adding **a) 1-***Z*, **b) 1**-*E*, **c)** rotaxane **2,** and **d)** axle **3** to DPPC LUVs at 25 °C and 45 °C. The LUVs are suspended in a buffer solution containing HEPES buffer (10 mM, pH 7.2) and NaCl (100 mM).

Table S36. Maximum fluorescence emission of sulforhodamine B at different time intervals upon addition of **1**-*Z* to DPPC LUVs at 25 °C.

Event	Trials (T)			Release %			Mean	Standard Deviation
	T1	T2	T3	T1	T2	T3		
LUVs	17191.11	17706.9	16554.79	0	0	0	0	0
+**1**-*Z*	19688.67	19700.84	18119.51	0.93657	0.74871	0.60217	0.76249	0.16763
After 5 min	20564.78	21836.55	19217.56	1.26511	1.55066	1.02475	1.28017	0.26328
After 10 min	23529.2	22903.62	22269.36	2.37676	1.95134	2.1992	2.17577	0.21367
After 15 min	24734.67	24409.83	22310.97	2.8288	2.51692	2.21522	2.52031	0.30681
+ Triton-X	283860.7	284022	276402.3	100	100	100	100	0.00E+00

Table S37. Maximum fluorescence emission of sulforhodamine B at different time intervals at 45 °C after the addition of **1**-*Z* to DPPC LUVs at 25 °C

Event	Trials (T)			Release %			Mean	Standard Deviation
	T1	T2	T3	T1	T2	T3		
LUVs	21806.82	22124.54	22269.36	0	0	0	0	0
+**1**-*Z*	26941.83	26475.63	25839.54	0.74224	0.61005	0.48912	0.6138	0.1266
After 5 min	34457.75	32668.27	35854.36	1.82862	1.47831	1.86118	1.7227	0.21228
After 10 min @ 45 °C	94952.71	97655.39	95722.35	10.57281	10.58995	10.06325	10.40867	0.29927
After 15 min @ 45 °C	113981.2	128045.8	135755.8	13.32326	14.8509	15.54793	14.57403	1.13788
+ Triton-X	713637.1	735355.8	752182.9	100	100	100	100	8.20E-15

Table S38. Maximum fluorescence emission of sulforhodamine B at different time intervals after the addition of **1**-*Z* to DPPC LUVs, followed by **1**-*Z* → **1**-*E* (one photoswitching) at 25 °C.

Event	Trials (T)			Release %			Mean	Standard Deviation
	T1	T2	T3	T1	T2	T3		
LUVs	25442.4	24790.328	24618.145	0	0	0	0	0
+**1**-*Z*	26941.834	26475.635	25940.557	0.65868	0.77148	0.57457	0.66824	0.0988
After 5 min	32235.469	28610.08	29524.125	2.98411	1.74856	2.13157	2.28808	0.63247
After the irr. @467 nm	34457.746	30582.846	31476.549	3.96033	2.65162	2.97987	3.19727	0.6809
After 4 min	36588.863	31504.902	33290.586	4.8965	3.07371	3.76804	3.91275	0.91997
After 10 min	38694.215	33290.586	35854.363	5.82136	3.89114	4.88196	4.86482	9.65E-01
+ Triton-X	253083.72	243242.06	254776.27	100	100	100	100	0

Table S39. Maximum fluorescence emission of sulforhodamine B at different time intervals at 45 °C after the addition of **1**-*Z* to DPPC LUVs followed by **1**-*Z* → **1**-*E* (one photoswitching) at 25 °C.

Event	Trials (T)			Release %			Mean	Standard Deviation
	T1	T2	T3	T1	T2	T3		
LUVs	23777.46	23672.94	23156.84	0	0	0	0	0
+**1**-*Z*	24957.73	26495.84	23704.99	0.53137	1.08754	0.2433	0.62074	0.42916
After 5 min	31741.81	29617.98	27917.43	3.58566	2.29037	2.11303	2.66302	0.80393
After the irr. @467 nm	34783.25	33311.29	31205.48	4.95496	3.71325	3.57246	4.08022	0.76081
After 4 min @ 45 °C	48129.29	59646.4	57372.98	10.96354	13.85904	15.18711	13.33656	2.15972
After 10 min @ 45 °C	65157.57	74333.48	73634.7	18.62991	19.51735	22.40501	20.18409	1.97E+00
+ Triton-X	245894	283239.6	248454.1	100	100	100	100	7.11E-15

Table S40. Maximum fluorescence emission of sulforhodamine B at different time intervals upon addition of rotaxane **2** to DPPC LUVs at 25 °C.

Event	Trials (T)			Release %			Mean	Standard Deviation
	T1	T2	T3	T1	T2	T3		
LUVs	15243.85	13842.56	13856.99	0	0	0	0	0
+**2**	18766.99	18800.74	20674.88	0.3977	0.38679	0.58384	0.45611	0.11075
After 5 min	26780.97	25826.07	32511.7	1.30235	0.93484	1.59746	1.27822	0.33197
After 10 min	30814.45	37320.9	39394.28	1.75766	1.83156	2.18684	1.92536	0.22945
After 15 min	34156.45	39670.16	40214.27	2.13492	2.01483	2.25706	2.1356	0.12112
+ Triton-X	901112.5	1295719	1181627	100	100	100	100	7.11E-15

Table S41. Maximum fluorescence emission of sulforhodamine B at different time intervals at 45 °C after the addition of rotaxane **2** to DPPC LUVs at 25 °C

Event	Trials (T)			Release %			Mean	Standard Deviation
	T1	T2	T3	T1	T2	T3		
LUVs	14442.44	14097.82	14376.34	0	0	0	0	0
+**2**	17951.6	16440.85	17270.71	0.43444	0.28941	0.35635	0.36007	0.07258
After 5 min	23426.27	20910.11	21605.17	1.11222	0.84146	0.89001	0.94789	0.14436
After 10 min @ 45 °C	99701.95	105301.7	100483.3	10.55531	11.26558	10.60138	10.80742	0.39745
After 15 min @ 45 °C	105341.8	107331	105141.7	11.25353	11.51625	11.17492	11.3149	0.17875
+ Triton-X	822182.9	823677.2	826600.2	100	100	100	100	0.00E+00

Table S42. Maximum fluorescence emission of sulforhodamine B at different time intervals upon addition of axle **3** to DPPC LUVs at 25 °C.

Event	Trials (T)			Release %			Mean	Standard Deviation
	T1	T2	T3	T1	T2	T3		
LUVs	22201.496	22647.096	22597.289	0	0	0	0	0
+**3**	22377.559	22665.313	23389.65	0.07834	0.00828	0.35211	0.14624	0.1817
After 5 min	23320.682	23298.096	23359.688	0.49801	0.29577	0.3388	0.37753	0.10654
After 10 min	22905.244	23429.912	23574.402	0.31315	0.35566	0.43421	0.36767	0.06142
After 15 min	23172.145	24519.932	23729.854	0.43192	0.85088	0.5033	0.59536	0.22414
+ Triton-X	246931.61	242752.34	247627.16	100	100	100	100	0.00E+00

Table S43. Maximum fluorescence emission of sulforhodamine B at different time intervals at 45 °C after the addition of axle **3** to DPPC LUVs at 25 °C

Event	Trials (T)			Release %			Mean	Standard Deviation
	T1	T2	T3	T1	T2	T3		
LUVs	22017.4	22171.88	22171.88	0	0	0	0	0
+**3**	22319.4	22222.75	22222.75	0.1329	0.01591	0.01619	0.055	0.06746
After 5 min	23343.36	22483.25	22483.25	0.58348	0.09737	0.09908	0.25998	0.28017
After 10 min @ 45 °C	48540.47	51093.33	51093.33	11.67132	9.04385	9.20311	9.97276	1.47315
After 15 min @ 45 °C	51275.38	61692.03	61692.03	12.87479	12.35811	12.57573	12.60288	0.25941
+ Triton-X	249267.5	341963.1	336429.1	100	100	100	100	0.00E+00

S3. Investigation of the behavior of axle 3 in LUVs upon light irradiation

S3.1. Sulforhodamine B release from EYPC/Chol 8:2 LUVs with 370 nm and 467 nm irradiations

The same procedure was followed as described in section **S2.2.2** with axle **3**, except for 5 irradiation cycles alternating between 370 nm and 467 nm. Instead, two separate studies were conducted, with EYPC/Chol 8:2 LUVs containing axle **3**. Ten irradiations were applied at 370 nm and ten at 467 nm, matching the total number of irradiations performed in the 5-cycle procedure (Figure main manuscript).

Table S44. Maximum fluorescence emission of sulforhodamine B released from EYPC/Chol 8:2 LUVs over five irradiation cycles after the addition of DMSO (control).

Event	Trials (T)			Release %			Mean	Standard Deviation
	T1	T2	T3	T1	T2	T3		
LUVs	39698.81	40539.5	41373.25	0	0	0	0	0
+DMSO	40900.71	40729.57	41438.43	0.66822	0.10684	0.03744	0.27083	0.34589
After 5 min	43855.96	41266.73	42234.43	2.31125	0.40879	0.49459	1.07154	1.07448
After 1st irr. @370 nm	44928.45	42892.52	43005.06	2.90752	1.32268	0.93716	1.72245	1.04424
After 5 min	45692.93	43294.93	42394.62	3.33255	1.54888	0.58658	1.82267	1.39331
After 1st irr. @467 nm	46833.75	43963.34	42878.88	3.96681	1.9246	0.8647	2.25204	1.57677
After 5 min	47151.93	43444.98	42553.2	4.14371	1.63323	0.67765	2.15153	1.79022
After 2nd irr. @370 nm	47317.53	44548.67	42430	4.23578	2.25363	0.6069	2.36544	1.81702
After 5 min	47416.23	45630.96	42253.3	4.29065	2.862	0.50542	2.55269	1.91148
After 2nd irr. @467 nm	47423.05	46285.73	42696.91	4.29444	3.23006	0.76019	2.76156	1.81311
After 5 min	47479.32	46598.68	42969.73	4.32573	3.40598	0.91687	2.88286	1.76361
After 3rd irr. @370 nm	47492.51	46029.17	44394.74	4.33306	3.08585	1.73527	3.05139	1.29924
After 5 min	47654.95	46538.27	43466.18	4.42337	3.37202	1.20199	2.99913	1.64275
After 3rd irr. @ 467 nm	47781.13	46228.17	44242.98	4.49353	3.19771	1.64811	3.11311	1.42459
After 5 min	47845.92	46631.32	44733.73	4.52955	3.42432	1.92995	3.29461	1.30464
After 4th irr. @ 370 nm	47914.13	46664.27	45347.91	4.56747	3.44285	2.28268	3.431	1.14244
After 5 min	48246.47	47154.55	44923.33	4.75224	3.71844	2.03884	3.50318	1.36945
After 4th irr. @ 467 nm	48556.96	47267.04	45726.45	4.92486	3.78168	2.50008	3.73554	1.21305
After 5 min	48666.09	47302.09	45794.14	4.98554	3.80138	2.53895	3.77529	1.2235
After 5th irr. @ 370 nm	48948.99	47296.87	46551.73	5.14282	3.79844	2.97404	3.97177	1.09473
After 5 min	49356.66	46790.45	46538.09	5.36948	3.51377	2.96621	3.94982	1.25957
After 5th irr. @ 467 nm	50204.11	46492.64	46725.67	5.84063	3.34637	3.07394	4.08698	1.5248
After 5 min	50424.07	46662.56	46814.88	5.96293	3.44189	3.12517	4.17666	1.55503
+Triton X	219564.6	218437.8	215495.7	100	100	100	100	0.00E+00

Table S45. Maximum fluorescence emission of sulforhodamine B released from EYPC/Chol 8:2 LUVs over ten 370 nm irradiations after the addition of axle **3**.

Event	Trials (T)			Release %			Mean	Standard Deviation
	T1	T2	T3	T1	T2	T3		
LUVs	42229.22	39717.76	41032.22	0	0	0	0	0
+**3**	45353.25	46625.05	41707.72	1.60127	3.67788	0.41278	1.89731	1.65256
After 5 min	48169.89	48966.19	45131.36	3.04498	4.92446	2.50489	3.49144	1.27007
After 1st irr. @370 nm	49147.7	48870.7	44739.18	3.54617	4.87362	2.26524	3.56168	1.30426
After 5 min	49781.24	50174.84	47259.2	3.87091	5.56802	3.80517	4.4147	0.99935
After 2nd irr. @370 nm	49923.4	51257.88	47941.41	3.94377	6.14471	4.22205	4.77017	1.19848
After 5 min	50040.42	51849.56	49455.56	4.00375	6.45975	5.14731	5.20361	1.22897
After 3rd irr. @370 nm	50816.25	52088.39	48852.92	4.40142	6.58692	4.77905	5.2558	1.16815
After 5 min	50816.25	52342.29	50579.24	4.40142	6.72211	5.83397	5.6525	1.17094
After 4th irr. @370 nm	51987.98	52380.03	50508.66	5.00201	6.74221	5.79084	5.84502	0.87137
After 5 min	51893.89	53346.6	51873.43	4.95378	7.25687	6.62482	6.27849	1.18997
After 5th irr. @370 nm	52333.82	53479.66	51962.1	5.17927	7.32772	6.679	6.39533	1.10196
After 5 min	53307.45	53953.16	52067.62	5.67832	7.57985	6.74348	6.66722	0.95306
After 6th irr. @ 370 nm	53239.51	54443.06	52826.6	5.6435	7.8407	7.20728	6.89716	1.13095
After 5 min	53868.43	55368.55	53735.29	5.96586	8.33349	7.76256	7.35397	1.23557
After 7th irr. @ 370 nm	54869.25	56676.78	56761.47	6.47885	9.03008	9.61179	8.37357	1.66646
After 5 min	56557.33	62290.6	61162.98	7.3441	12.01924	12.30145	10.55493	2.78424
After 8th irr. @ 370 nm	58037.43	65485.97	63079.74	8.10275	13.72066	13.47274	11.76538	3.17436
After 5 min	62470.3	75687.59	71068.31	10.37488	19.15266	18.35438	15.96064	4.85385
After 9th irr. @ 370 nm	74038.49	79997.83	75859.48	16.30434	21.44771	21.28216	19.67807	2.9229
After 5 min	94834.16	96276.7	90422.41	26.96349	30.11563	30.18123	29.08678	1.83912
After 10th irr. @370 nm	105136.7	102881.3	98304.39	32.24422	33.63234	34.99774	33.62477	1.37677
After 5 min	126430.1	122102.6	119299.4	43.15848	43.86699	47.82728	44.95092	2.51607
+Triton X	237326.1	227523.7	204677.6	100	100	100	100	7.11E-15

Table S46. Maximum fluorescence emission of sulforhodamine B released from EYPC/Chol 8:2 LUVs over ten 467 nm irradiations after the addition of axle **3**.

Event	Trials (T)			Release %			Mean	Standard Deviation
	T1	T2	T3	T1	T2	T3		
LUVs	42380.79	40809.21	39669.73	0	0	0	0	0
+**3**	43025.29	41961.52	40406.46	0.36024	0.64917	0.43122	0.48021	0.15057
After 5 min	44018.59	44318.88	43335.8	0.91544	1.97723	2.14581	1.67949	0.66704
After 1st irr. @467 nm	44403.44	45042.69	43793.84	1.13055	2.385	2.41391	1.97649	0.73274
After 5 min	45665.06	47195.87	45927.95	1.83573	3.59802	3.66304	3.03226	1.03674
After 2nd irr. @467 nm	46260.77	47573.14	46253.71	2.1687	3.81056	3.85371	3.27766	0.96063
After 5 min	47986.23	48932.09	48418.89	3.13314	4.57615	5.12103	4.27677	1.0272
After 3rd irr. @467 nm	48246.83	49803.32	48341.69	3.2788	5.06697	5.07584	4.47387	1.03497
After 5 min	50266.51	51029.91	49891.53	4.40769	5.75799	5.98299	5.38289	0.85201
After 4th irr. @467 nm	50928.7	50968.01	49805.11	4.77782	5.72312	5.93241	5.47778	0.61515
After 5 min	53416.21	52802.73	52238.33	6.1682	6.75673	7.35661	6.76052	0.59421
After 5th irr. @467 nm	54929.02	53174.15	53245.32	7.01378	6.96598	7.94602	7.30859	0.55254
After 5 min	56707.48	57365.34	56433.81	8.00784	9.32715	9.81229	9.04909	0.93381
After 6th irr. @ 370 nm	58751.48	58854.41	57566.35	9.15033	10.16604	10.47519	9.93052	0.69312
After 5 min	63836.29	65594.61	63079.54	11.99246	13.96323	13.70215	13.21928	1.07045
After 7th irr. @ 467 nm	68900.85	66668.84	64162.3	14.82328	14.56842	14.33591	14.57587	0.24377
After 5 min	77182.16	75272.92	70967.71	19.45208	19.41566	18.31923	19.06232	0.6438
After 8th irr. @467 nm	81255.7	79658.51	74969.11	21.72897	21.88635	20.66131	21.42554	0.6665
After 5 min	94367.43	89689.2	85539.5	29.05772	27.53729	26.84834	27.81445	1.13047
After 9th irr. @467 nm	100600.5	94939.07	90704.45	32.54165	30.49488	29.87147	30.96934	1.39689
After 5 min	121915.9	107722.6	104586	44.45581	37.69666	37.99657	40.04968	3.81877
After 10th irr. @467 nm	128780.4	115847.7	112515.9	48.29271	42.27406	42.63809	44.40162	3.37469
After 5 min	143136.4	129678.5	127224.8	56.31693	50.06587	51.24745	52.54342	3.32093
+Triton X	221289	218313.9	210517.4	100	100	100	100	0.00E+00

S3.2. Changes in absorbance of axle 3 over light irradiation

To study the behavior of axle **3** in LUVs using UV spectroscopy, the EYPC/Chol 8:2 LUVs were prepared as described in section **S1.1**. Several experiments were conducted to examine and eliminate the effect of light irradiation on the background and interactions between medium and axle **3**. Therefore, different sample preparations were performed as outlined below. Each sample was irradiated at 370 nm and 467 nm alternately (one switching cycle) five times unless otherwise specified. Absorbance was recorded after each 1-minute irradiation. 10 mol% of axle **3** equals 20 μM, which has been used in solution-phase studies (**experiments 5 and 6**). Rotaxane **2** in DMSO was studied following the same procedure.

Experiment 1. 10 mol % of axle **3** with LUVs (**Figure 6c** and **d**)

Sample: Axle **3** in DMSO (6 μL, 10 mM) + LUVs (24 μL, 25 mM of lipid content) in HEPES buffer + HEPES buffer (2970 μL, 10 mM, pH 7.2).

Blank: DMSO (6 μL, 10 mM) + LUVs (24 μL, 25 mM of lipid content) in HEPES buffer + HEPES buffer (2970 μL, 10 mM, pH 7.2).

Experiment **1** was repeated with ten 1-minute irradiations at 467 nm instead of irradiation cycles.

Experiment 2: LUVs suspended in HEPES buffer

Sample: LUVs (24 μL, 25 mM of lipid content) in HEPES buffer + HEPES buffer (2976 μL, 10 mM, pH 7.2).

Blank: HEPES buffer (3000 μL, 10 mM, pH 7.2).

Experiment 3: DMSO in HEPES buffer

Sample: DMSO (6 μL, 10 mM) + HEPES buffer (2994 μL, 10 mM, pH 7.2).

Blank: HEPES buffer (3000 μL, 10 mM, pH 7.2).

Experiment 4: DMSO with LUVs suspended in HEPES buffer

Sample: DMSO (6 µL, 10 mM) + LUVs (24 µL, 25 mM of lipid content) in HEPES buffer + HEPES buffer (2970 µL, 10 mM, pH 7.2).

Blank: LUVs (24 µL, 25 mM of lipid content) in HEPES buffer + HEPES buffer (2976 µL, 10 mM, pH 7.2).

Experiment 5: Axle **3** (20 µM) in DMSO

Sample: Axle **3** in DMSO (6 µL, 10 mM) + DMSO (2994 µL)

Blank: DMSO (3000 µL)

Experiment 6: Rotaxane **2** (20 µM) in DMSO

Sample: Axle **3** in DMSO (6 µL, 10 mM) + DMSO (2994 µL)

Blank: DMSO (3000 µL)

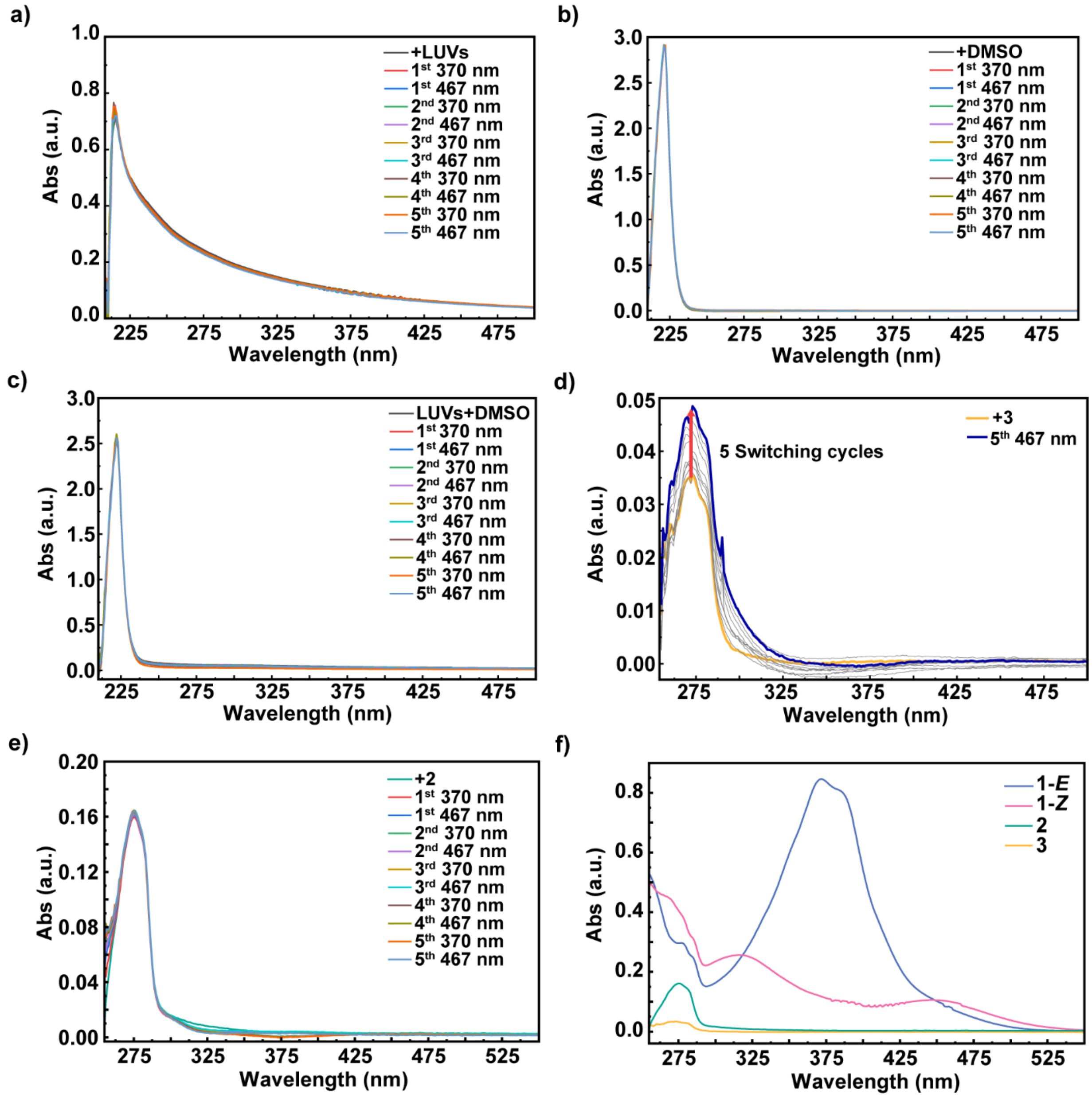

Figure S23: Effect of five light irradiation cycles at 370 nm and 467 nm alternatively on **a)** LUVs suspended in HEPES buffer (**experiment 2**), **b)** DMSO in HEPES buffer (**experiment 3**)**, c)** DMSO in LUVs suspended in HEPES buffer (**experiment 4**), **d)** axle **3** (20 μM) in DMSO (**experiment 5**) where the blue trace shows the last irradiation at 467 nm, **e)** Rotaxane **2** (20 μM) in DMSO (**experiment 6**), and **f)** UV absorption spectra of **1**-*E*, **1**-*Z*, rotaxane **2**, and axle **3** at 20 μM in DMSO.

S4. Characterization and investigation of rotaxane 4

S4.1. Synthetic scheme of rotaxane 4

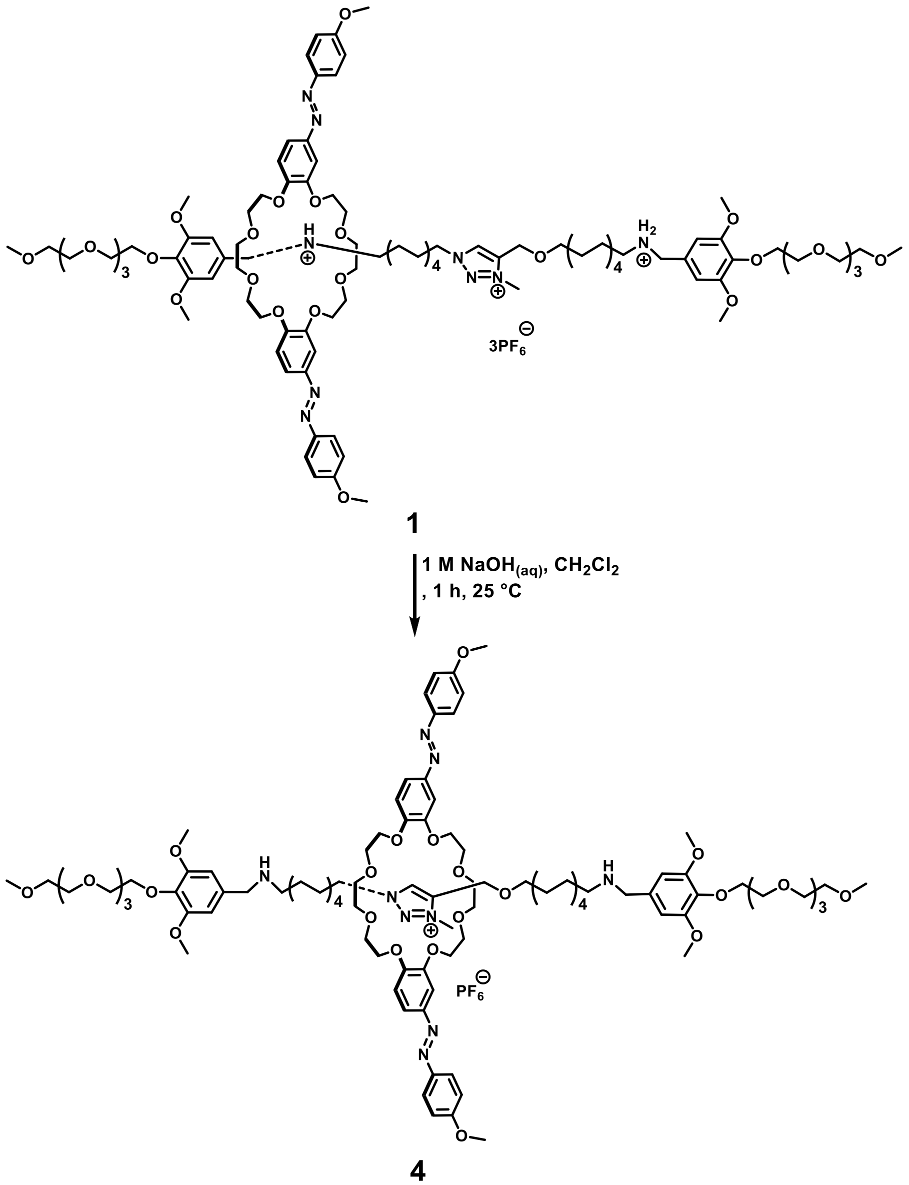

Figure S24: Synthesis of rotaxane **4**

S4.2. Synthetic procedure of rotaxane 4

Rotaxane **1** (52 mg, 0.024 mmol, 1 equiv) was dissolved in dichloromethane (3 mL) in a 20 mL glass vial. Then, an aqueous sodium hydroxide solution (8 mL, 1 M) was added to the vial and stirred vigorously for 1 hour. After that, the reaction mixture was transferred to a separatory funnel and extracted with dichloromethane (5 mL). Once the organic layer was collected, it was dried over magnesium sulfate to obtain rotaxane **4** as a dark orange oil (28 mg, 55%). ^{1}H NMR (400 MHz, acetonitrile-d_3): δ_{ppm} = 9.01 (s, 1H), 7.83 (dd, *J* = 8.9, 1.8 Hz, 4H), 7.47 (d, *J* = 8.6 Hz, 2H), 7.41 (app s, 2H), 7.05 (d, *J* = 8.6 Hz, 4H), 7.00 (d, *J* = 8.6 Hz, 2H), 6.59 (d, *J* = 4.2 Hz, 4H), 4.97 (app s, 1H), 4.68 – 4.46 (m, 2H), 4.16 (app s, 10H), 3.97 (t, *J* = 4.8 Hz, 4H), 3.85 (app s, 8H), 3.81 – 3.71 (m, 18H), 3.70 – 3.61 (m, 6H), 3.62 – 3.54 (m, 8H), 3.57 – 3.50 (m, 16H), 3.45 (dd, *J* = 5.7, 3.5 Hz, 8H), 3.28 (s, 6H), 2.57 – 2.45 (m, 2H), 2.40 (app s, 1H), 2.17 – 1.97 (m, 4H), 1.49 (app s, 2H), 1.41 (t, *J* = 6.7 Hz, 3H), 1.34 – 1.13 (m, 20H), 1.12 – 0.90 (m, 10H); ^{13}C NMR (126 MHz, acetonitrile-d_3): δ_{ppm} = 163.07, 154.23, 154.00, 150.79, 149.78, 149.06, 147.88, 147.63, 141.99, 136.71, 129.96, 129.27, 126.69, 125.25, 125.15, 122.33, 120.45, 115.47, 115.05, 112.83, 111.46, 107.66, 107.47, 106.41, 106.38, 104.10, 73.13, 72.98, 72.61, 72.30, 71.76, 71.39, 71.30, 71.23, 71.21, 71.18, 71.15, 71.08, 70.99, 70.46, 69.62, 69.42, 69.18, 60.96, 58.90, 57.08, 56.87, 56.69, 56.60, 56.45, 54.79, 54.12, 53.32, 49.80, 39.11, 30.22, 30.10, 30.03, 29.93, 29.85, 29.43, 28.00, 27.40, 26.68, 26.52, 23.40; HR-ESI-TOF MS: m/z (%) calcd. for $[C_{98}H_{152}N_9O_{25}]^{3+}$ $[M+2H]^{+3}$ 618.6972, found 618.6974; calcd. for $[C_{98}H_{150}N_9O_{25}]^+$ $[M]^+$ 1854.0771, found 1854.0706; calcd. for $[C_{98}H_{151}F_6N_9O_{25}P]^+$ $[M+H+PF_6]^+$ 2000.0491, found: 2000.0391.

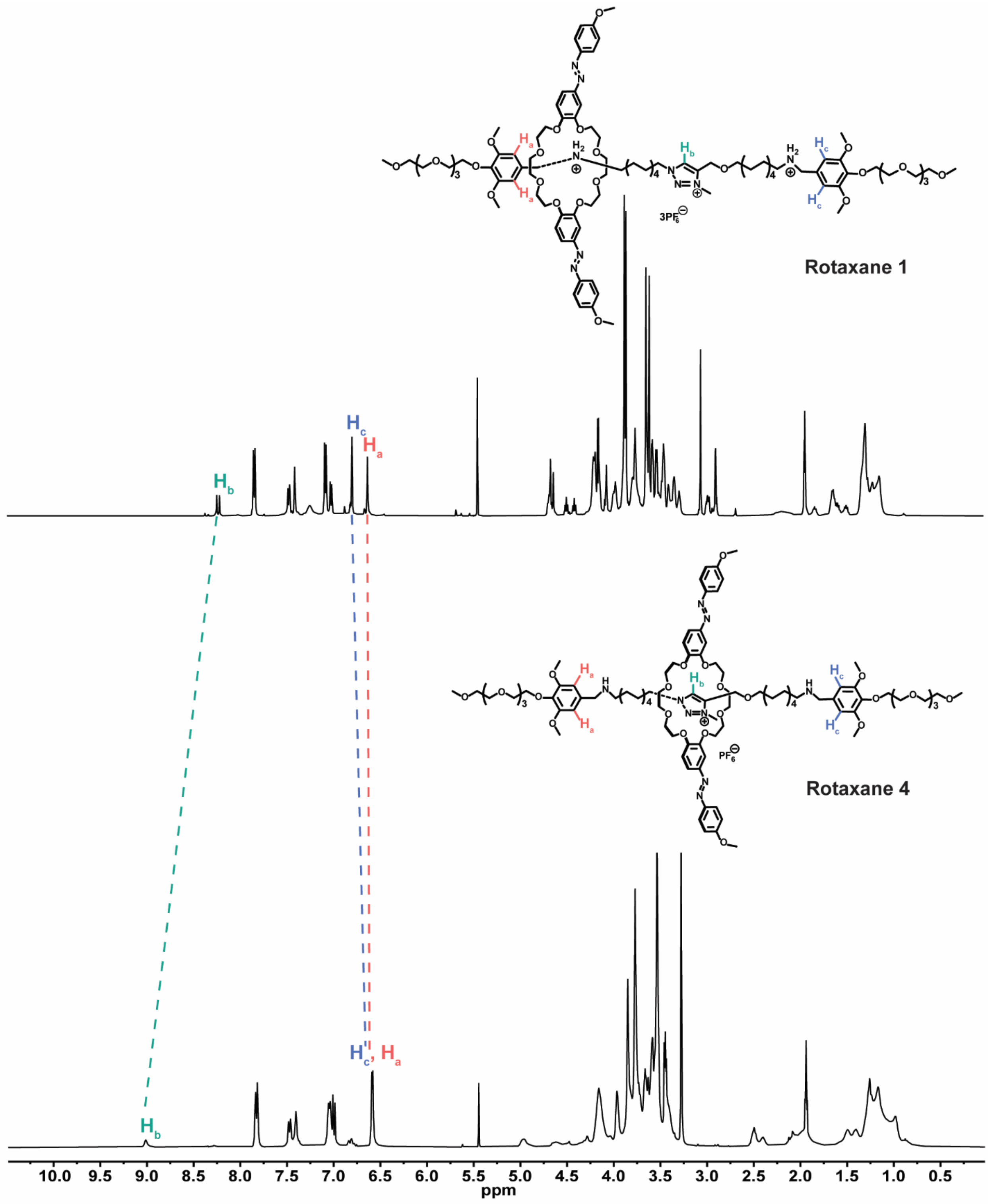

Figure S25: Confirmation of formation of rotaxane **4**

S4.3. ^{1}H NMR, ^{13}C NMR, and mass spectra of rotaxane 4

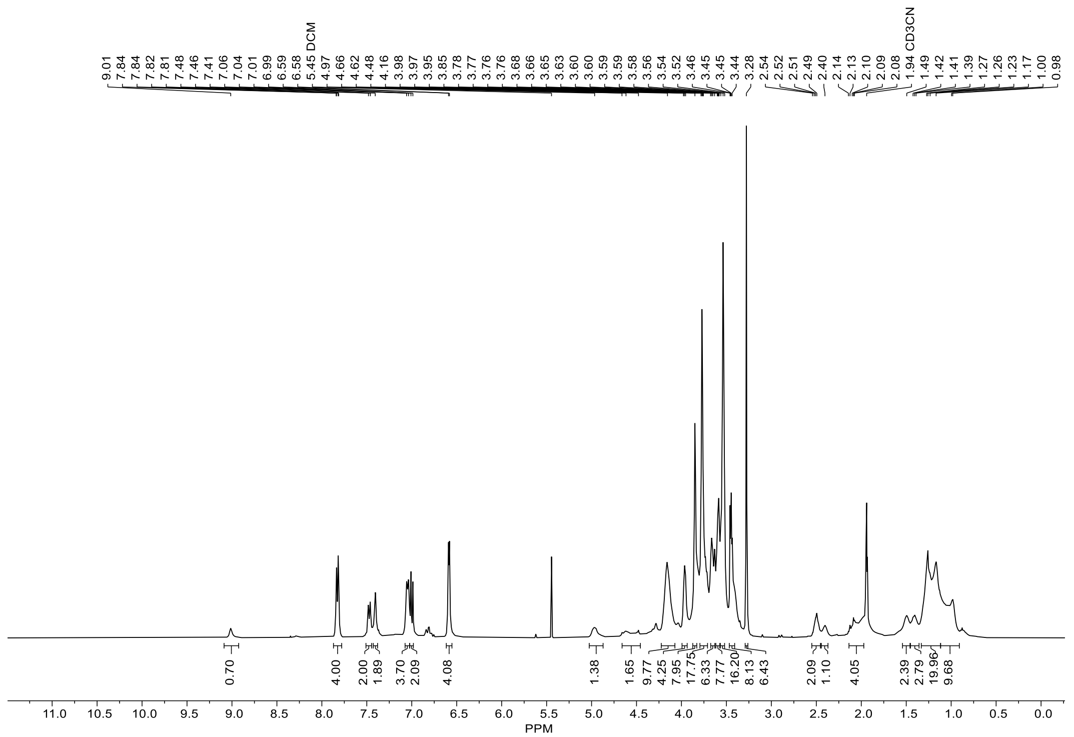

Figure S26: ^{1}H NMR spectrum (400 MHz, 298 K) of rotaxane **4** in acetonitrile-d_3.

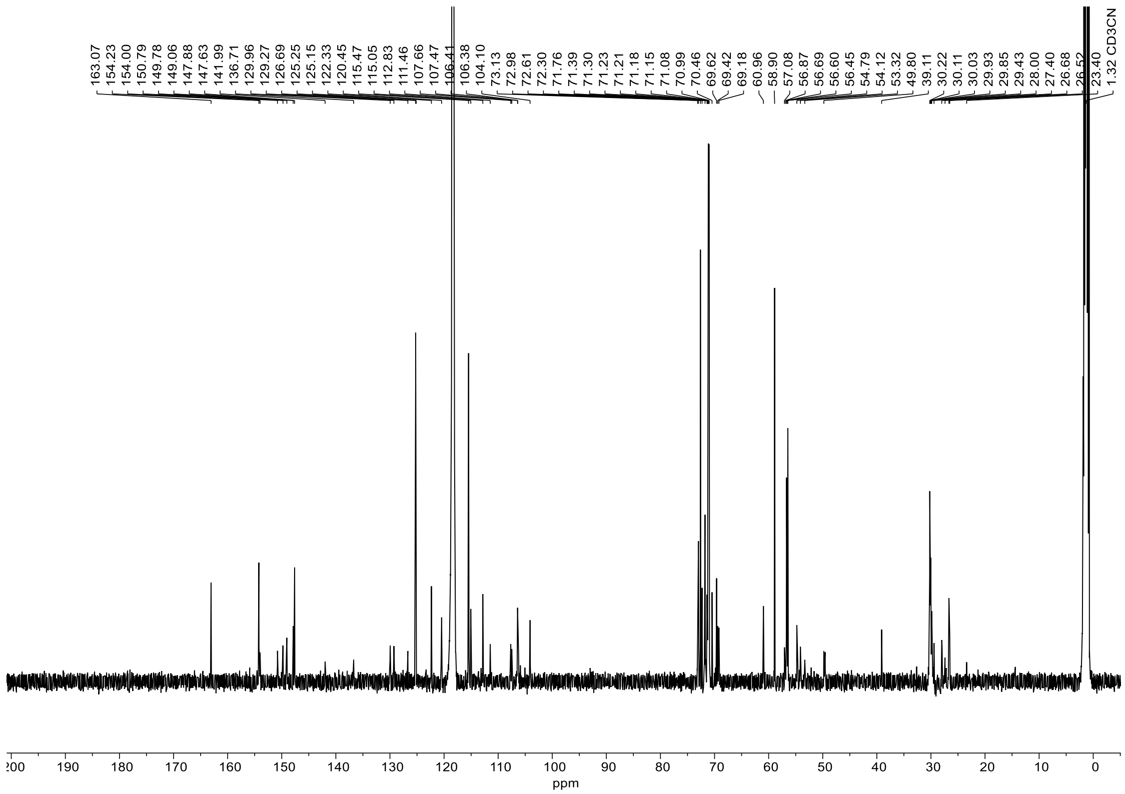

Figure S27: ^{13}C NMR spectrum (126 MHz, 298 K) of rotaxane **4** in acetonitrile-d_3.

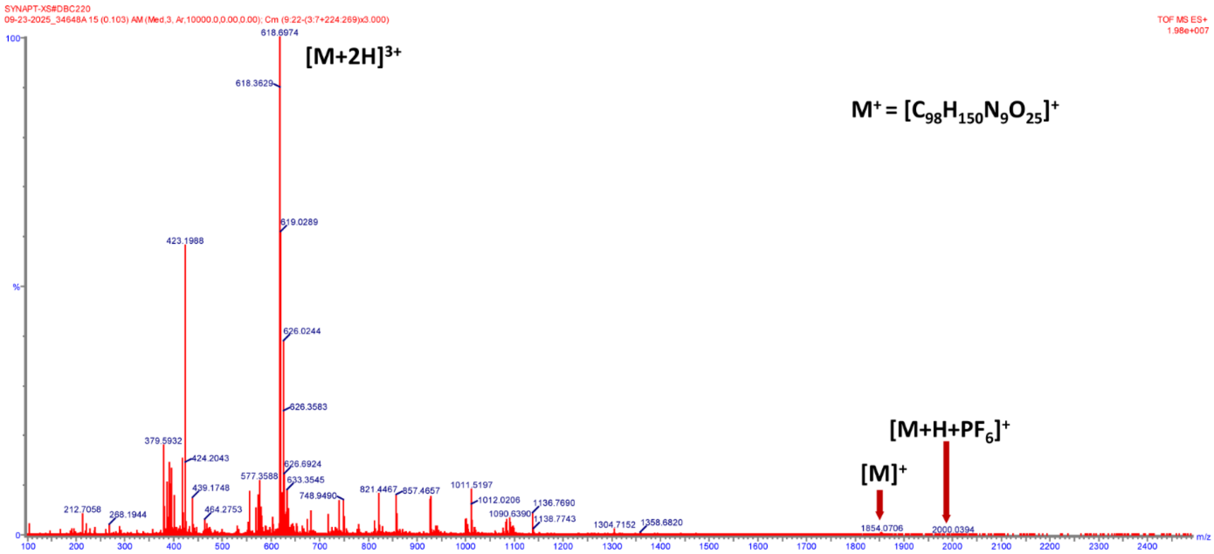

Figure S28: HRMS spectrum of rotaxane **4**

S4.4. Photoswitching studies in solution by UV–vis spectroscopy

Solution of rotaxane **4** was prepared in dry acetonitrile (30 μM, 3 mL) and placed in 3.5 mL quartz cuvettes individually. The initial absorption spectrum was measured at 25 °C, with no light exposure. Each sample was then irradiated at 370 nm for 5 minutes to reach $PSS_{370\ nm}$, with its absorption spectrum subsequently recorded. Next, the samples were irradiated at 467 nm for 5 minutes to attain $PSS_{467\ nm}$, and their spectra were recorded. The irradiation was provided by a Kessil LED positioned 8 cm from the cuvette, with the long axis of the cuvette perpendicular to the LED beam.

To evaluate photostability over multiple cycles, rotaxane **4** was alternately irradiated at 370 nm and 467 nm for 5 minutes each, completing 20 photoswitching cycles. After each cycle, the absorption spectrum was recorded, and the absorbance at 365 nm was plotted.

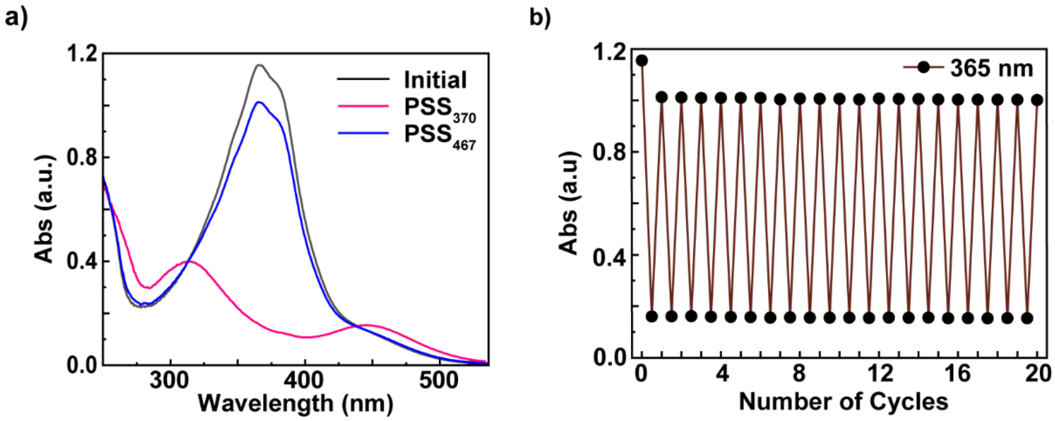

Figure S29: UV–vis absorption spectra showing reversible photoswitching of rotaxane **4** in acetonitrile. **a)** UV–vis spectral changes of rotaxane **4** after one cycle of photoisomerization, and **b)** changes in the absorbance at 365 nm over twenty photoswitching cycles at 25 °C. All samples have a concentration of 30 μM.

S4.5. *Z*→*E* Thermal isomerization in solution

A solution of rotaxane **4** was prepared in dry acetonitrile (30 μM, 3 mL) and placed in separate 3.5 mL quartz cuvettes. Each cuvette was then irradiated at 370 nm for 5 minutes to produce the $PSS_{370\ nm}$, followed by recording the absorption spectrum. Afterwards, the cuvette was immersed in a water bath at 40 °C for 5 minutes, and the spectrum was recorded again. This cycle of heating at 40 °C for 5 minutes and spectral measurement was repeated until no further changes appeared in the absorption spectrum. The entire process to regenerate the *E* isomer of rotaxane **4** took a total of 85 minutes at 40 °C (excluding measurement time).

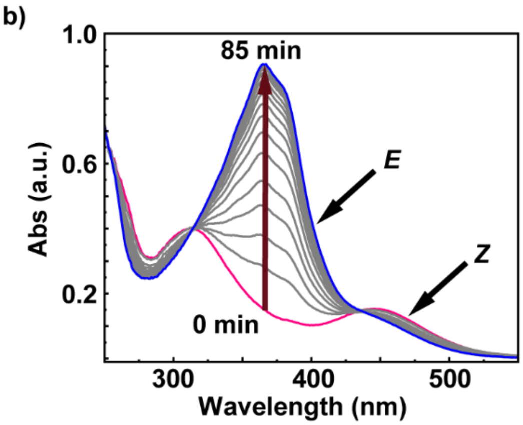

Figure S30: UV–vis absorption spectra showing progression of the thermal *Z*→*E* isomerization of rotaxane **4** (30 µM) over time at 40 °C in acetonitrile.

S4.6. Photoswitching reversibility in lipid bilayers monitored by UV–vis spectroscopy

The photoswitching of rotaxane **4** in EYPC/Chol 8:2 LUVs was measured following the same procedure described in section **S1.2.**

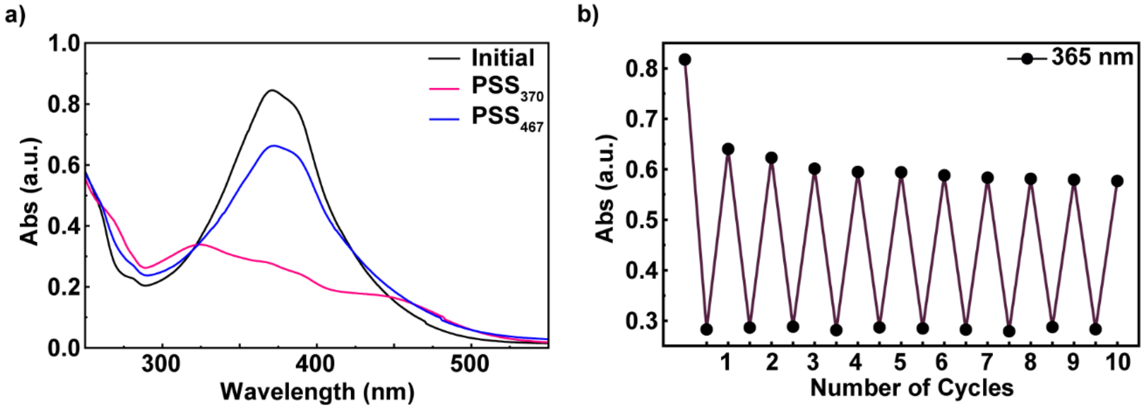

Figure S31: Reversible photoswitching of rotaxane **4** in EYPC/Chol 8:2 LUVs. a) UV–vis spectral changes of rotaxane **4** after one cycle of photoisomerization and b) absorbance changes at 365 nm over ten consecutive photoisomerization cycles All experiments were performed at 25 °C using LUVs suspended in HEPES buffer (10 mM, pH 7.2), and the rotaxane (10 mol % with respect to the total lipid concentration) was added dissolved in DMSO.

S4.7. Time-dependent release of sulforhodamine B from LUVs without light irradiation

To evaluate the individual effects of the *E*- and *Z*-configurations of rotaxane **4** on the permeability of EYPC/Chol 8:2 LUVs, the percentage of sulforhodamine B released at 5-minute intervals over a total duration of 70 minutes was measured following the same procedure described in section **S2.2.**

Table S47. Maximum fluorescence emission of sulforhodamine B at different time intervals upon addition of **4-***E* to EYPC 8:2 LUVs.

Event	Trials (T)			Release %			Mean	Standard Deviation
	T1	T2	T3	T1	T2	T3		
LUVs only	32761.32	32751.03	33101.67	0	0	0	0	0
+**4**-*E*	86633.86	85652.09	79177.66	23.30873	22.40722	20.96705	22.22766	1.18112
After 5 min	89493.76	96214.07	93628.63	24.5461	26.88094	27.54301	26.32335	1.57434
After 10 min	105514.6	105088.8	96223.63	31.47774	30.64	28.72387	30.28054	1.41169
After 15 min	121826.4	118064.2	106244.9	38.53526	36.13597	33.2841	35.98511	2.62883
After 20 min	133355.8	129983.7	114898.4	43.52365	41.18468	37.2219	40.64341	3.18555
After 25 min	143905.7	141288.1	123710.5	48.08822	45.97286	41.23188	45.09765	3.51096
After 30 min	150539	147886.1	130520.7	50.95819	48.7676	44.33087	48.01888	3.3765
After 35 min	155262.2	153107	138282.5	53.00177	50.97897	47.86293	50.61456	2.58873
After 40 min	158183.2	156201.2	143844.7	54.26558	52.28958	50.39401	52.31639	1.93592
After 45 min	160453.4	158065.2	147752.8	55.24782	53.0791	52.17243	53.49978	1.58026
After 50 min	162673.1	161913.4	150972.1	56.20819	54.7091	53.63737	54.85155	1.29132
After 55 min	162679.3	163738.4	153764.5	56.21089	55.48212	54.90803	55.53368	0.65296
After 60 min	165126.2	165411.2	155813.1	57.26958	56.19064	55.84029	56.4335	0.74495
After 65 min	169482	172294.1	155847.9	59.15417	59.10602	55.8561	58.03876	1.89039
After 70 min	170428.1	175521	156040.5	59.56351	60.47285	55.94373	58.66003	2.39592
+ Triton-X	263887.4	268840.4	252856	100	100	100	100	8.20E-15

Table S48. Maximum fluorescence emission of sulforhodamine B at different time intervals upon addition of **4**-*Z* to EYPC 8:2 LUVs.

Event	Trials (T)			Release %			Mean	Standard Deviation
	T1	T2	T3	T1	T2	T3		
LUVs only	32879.27	33456.11	33217.65	0	0	0	0	0
+**4**-*Z*	74171.41	71970.09	54697.7	19.52728	18.90681	10.5324	16.32216	5.02367
After 5 min	95094.96	90361.69	79450.97	29.42214	27.9354	22.66976	26.67577	3.54805
After 10 min	97020.13	94689.23	98892.98	30.33256	30.05982	32.20284	30.86507	1.16654
After 15 min	98012.52	96791.65	99553.95	30.80187	31.09192	32.52694	31.47357	0.9237
After 20 min	98829.5	98218.77	99968.61	31.18822	31.7925	32.73026	31.90366	0.777
After 25 min	99998.78	97836.34	100013.2	31.74118	31.60476	32.75213	32.03269	0.62677
After 30 min	100006.4	100670.8	100253.1	31.74476	32.99623	32.86973	32.53691	0.68893
After 35 min	101073.2	100522.7	100940.2	32.24929	32.92353	33.20667	32.79317	0.49182
After 40 min	101721.9	99634.09	100253.1	32.55604	32.48729	32.86973	32.63769	0.20388
After 45 min	103111.4	101392.5	102315.4	33.21318	33.35051	33.88099	33.48156	0.35267
After 50 min	103181.8	103640.4	101966.7	33.24644	34.45404	33.70999	33.80349	0.60921
After 55 min	103183.5	100344.3	103176.1	33.24725	32.83594	34.30302	33.46207	0.75676
After 60 min	105481	88513.49	103295	34.33374	27.0281	34.3613	31.90772	4.22589
After 65 min	105833.2	92053.19	103660.4	34.50029	28.76577	34.54047	32.60218	3.32249
After 70 min	106230.4	93050.23	103720.4	34.68816	29.25523	34.56989	32.83776	3.10313
+ Triton-X	244338	237160.3	237160.3	100	100	100	100	0.00E+00

S4.8. Release of sulforhodamine B from LUVs with light irradiation

To evaluate the effect of photoisomerization of rotaxane **4** on the permeability of EYPC/Chol 8:2 LUVs, the percentage of sulforhodamine B released after five cycles of light irradiation was assessed at wavelengths of 370 nm and 467 nm following the same procedure described in section **S2.3.**

Table S49. Maximum fluorescence emission of sulforhodamine B released from EYPC: Chol 8:2 LUVs over five irradiation cycles after the addition of **4**-*E* to the LUVs.

Event	Trials (T)			Release %			Mean	Standard Deviation
	T1			T2				
	T1	T2	T3	T1	T2	T3		
LUVs	34476.84	35575.81	35303.23	0	0	0	0	0
+**4**-*E*	104295.9	108433	93942.38	34.38677	34.84422	32.52105	33.91735	1.23067
After 5 min	111767.4	121446.5	109038.6	38.06656	41.06798	40.89335	40.0093	1.68472
After 1st irr. @370 nm	114600.6	123548.5	112581.1	39.46197	42.07328	42.85804	41.46443	1.77802
After 5 min	116692.2	128854.9	114538.5	40.4921	44.61107	43.94361	43.01559	2.21074
After 1st irr. @467 nm	117105	128719.4	115336.6	40.69539	44.54627	44.38623	43.20929	2.17858
After 5 min	126089.1	139133.9	121266.9	45.12021	49.52706	47.67512	47.4408	2.21275
After 2nd irr. @370 nm	129375.2	138637.4	120902.2	46.73862	49.28961	47.47286	47.8337	1.31322
After 5 min	129944.5	140965.7	123095.3	47.01901	50.40312	48.68914	48.70375	1.6921
After 2nd irr. @467 nm	133738.4	141228.4	123195.2	48.88755	50.52873	48.74459	49.38696	0.99138
After 5 min	144248.8	147281.2	130572	54.06405	53.42352	52.83569	53.44109	0.61437
After 3rd irr. @370 nm	145036	146679.6	131449.3	54.45175	53.13582	53.32224	53.6366	0.71206
After 5 min	146078.4	145092.8	132069.5	54.96517	52.37692	53.6662	53.66943	1.29413
After 3rd irr. @ 467 nm	150346.3	151371.8	134690.9	57.06719	55.37986	55.12003	55.85569	1.0572
After 5 min	155696.2	155613.2	139904.9	59.70206	57.40835	58.01172	58.37404	1.18901
After 4th irr. @ 370 nm	155413.2	156162.3	137956.9	59.56269	57.67093	56.93132	58.05498	1.35707
After 5 min	155860.6	156792.6	139671.3	59.78302	57.97237	57.88215	58.54585	1.07237
After 4th irr. @ 467 nm	159537.3	159242.5	142719.5	61.59382	59.14407	59.57269	60.10353	1.3083
After 5 min	161123.7	160430.7	146932.7	62.37517	59.71232	61.90928	61.33226	1.42211
After 5th irr. @ 370 nm	163286.1	159656.3	146552.6	63.44019	59.34197	61.69847	61.49354	2.05678
After 5 min	160317.2	161010.9	148155.3	61.97794	59.98978	62.58731	61.51835	1.35839
After 5th irr. @ 467 nm	163698.7	164058.7	151592.8	63.64341	61.44741	64.49376	63.19486	1.57193
After 5 min	170470.8	165507.5	151792	66.97871	62.14031	64.60426	64.57443	2.41934
+Triton X	237517.3	244669.8	215614.6	100	100	100	100	7.11E-15

S5. References

1. Conthagamage, U. N. K.; Rajeshwar T, R.; van der Ham, S.; Akhtar, N.; Davis, M. L.; Jayawardana, S. G.; Lopez, L.; Vutukuri, H. R.; Smith, J. C.; Smith, M. D.; García-López, V. *Commun. Chem.* **2024**, *7*, 1–11.
